# A Novel Mechanism of Cannabidiol in Suppressing Hepatocellular Carcinoma by Inducing GSDME Dependent Pyroptosis

**DOI:** 10.3389/fcell.2021.697832

**Published:** 2021-07-19

**Authors:** Fugen Shangguan, Hongfei Zhou, Nengfang Ma, Shanshan Wu, Huimin Huang, Guihua Jin, Shijia Wu, Weilong Hong, Weiwei Zhuang, Hongping Xia, Linhua Lan

**Affiliations:** ^1^Key Laboratory of Diagnosis and Treatment of Severe Hepato-Pancreatic Diseases of Zhejiang Province, The First Affiliated Hospital of Wenzhou Medical University, Wenzhou, China; ^2^School of Life and Environmental Science, Wenzhou University, Wenzhou, China; ^3^Medical Research Center, The First Affliated Hospital of Wenzhou Medical University, Wenzhou, China; ^4^Department of Pathology in the School of Basic Medical Sciences, The Affiliated Sir Run Run Hospital, State Key Laboratory of Reproductive Medicine, Key Laboratory of Antibody Technique of National Health Commission, Nanjing Medical University, Nanjing, China

**Keywords:** cannabidiol, hepatocellular carcinoma, pyroptosis, integrative stress response, aerobic glycolysis

## Abstract

Cannabidiol (CBD), a phytochemical derived from *Cannabis sativa* L., has been demonstrated to exhibit promising anti-tumor properties in multiple cancer types. However, the effects of CBD on hepatocellular carcinoma (HCC) cells remain unknown. We have shown that CBD effectively suppresses HCC cell growth *in vivo* and *in vitro*, and induced HCC cell pyroptosis in a caspase-3/GSDME-dependent manner. We further demonstrated that accumulation of integrative stress response (ISR) and mitochondrial stress may contribute to the initiation of pyroptotic signaling by CBD. Simultaneously, CBD can repress aerobic glycolysis through modulation of the ATF4–IGFBP1–Akt axis, due to the depletion of ATP and crucial intermediate metabolites. Collectively, these observations indicate that CBD could be considered as a potential compound for HCC therapy.

## Introduction

Hepatocellular carcinoma (HCC) is an extremely malignant cancer, accounting for almost 95% of primary liver cancer cases. Data from the Global Cancer Statistics showed 841,000 new cases of liver cancer and 781,000 deaths were reported worldwide, accounting for 4.7 and 8.2% of all cancer cases and deaths, respectively (Bray et al., 2018). Even though many patients have gained effective diagnoses and therapy at an early stage, the recurrence rate is still high and the 5-year survival rate is only 3% (Siegel et al., 2019). Thus, in spite of the elucidation of the pathogenesis of HCC, the prognosis is still poor. Therefore, it is an urgent need to find early diagnostic markers and precise treatment strategies to improve the prognosis and quality of life for patients.

Cannabidiol (CBD), a phytochemical derived from *Cannabis sativa* L., has been shown to have anti-tumor activity and to be a potential compound for tumor therapy. Moreover, CBD has non-psychoactive effect, which is an advantage for clinical applications (Kis et al., 2019). Data has shown that CBD is mainly distributed in the lungs, heart, liver, and brain after absorption and metabolized in the liver (Zendulka et al., 2016). Multiple studies have shown that CBD and other cannabinoids display promising anti-tumor properties in a variety of tumor cells by inducing cell growth arrest, metastasis inhibition, and cell apoptosis (Kis et al., 2019). In lung cancer, CBD can depress cell invasion and tumor growth (Ramer et al., 2010, 2013). Indeed, comparison with cisplatin treatment has shown that CBD exposure can repress melanoma tumor growth to a similar extent (Blázquez et al., 2006; Armstrong et al., 2015; Simmerman et al., 2018). In breast cancer, CBD can trigger cell apoptosis by promoting interactions with PPARγ, mTOR, and cyclin D1 (Sultan et al., 2018; Jeong et al., 2019c). Furthermore, the increased activities of the antioxidant enzymes SOD and GPX in CT26 colon cancer cells, accompanied by growth inhibition, may indicate that CBD operates *via* a novel mechanism (Honarman et al., 2019). Interestingly, another study observed that CBD can reduce the growth of PC3 prostate cancer cells by regulating exosome release (Kosgodage et al., 2018). CBD has also been reported to prevent invasion and mediate cell apoptosis *via* stimulation of serotonin and vanilloid receptors in SH SY5Y and IMR-32 brain cancer cell lines (Alharris et al., 2019). Meanwhile, previous study has demonstrated that cannabinoids derived from Cannabis Sativa can modulate the TRP channels to perform physical function (Muller et al., 2019). Consistently, CBD, a kind of cannabinoids, has been verified to regulate cell fate through modulating TRP family. Ligresti et al. (2006) have shown that CBD may induce breast cancer cell apoptosis by modulating TRPV1 and/or CB2. Moreover, CBD can improve the chemotherapeutic sensitivity in TRPV2 over-expressed endometrial cancer cells (Oliviero et al., 2020). Additionally, CBD can also increase proliferation, migration, tubulogenesis, and integrity of human brain endothelial cells through TRPV2 activation (Luo et al., 2019). Meanwhile, Hassan et al. (2014) have demonstrated that CBD treatment increased the expression of TRPV2 and TRPV1 proteins and caused a translocation of TRPV2 to the cell membrane in BV-2 cell. All these observations indicate that CBD may be a promising drug for cancer therapy. While it has been reported in the literature that CBD has anti-tumor activity in many cancer cell lines, it is largely unknown whether this is the case in HCC cells. Furthermore, while CBD induced apoptosis has been shown, it is unknown whether it could also trigger pyroptosis. Pyroptosis has been shown to be a kind of programmed necrosis mediated by the gasdermin family including GSDME and GSDMD (Shi et al., 2017). GSDMD can be activated by caspase-1/4/5/11, and GSDME is regulated by caspase-3, which mediates the induction of pyroptosis. This form of cell death is characterized by cell swelling and bubbles from the plasma membrane (Shi et al., 2015; Ding et al., 2016; Liu et al., 2016; Rogers et al., 2017; Wang et al., 2017; Robinson et al., 2019). Both GSDMD-N and GSDME-N oligomerize and form large pores on the plasma membrane to induce plasma membrane disruption, leading to the release of cellular contents, including pro-inflammatory mediators, and lactate dehydrogenase (LDH) (Zhang et al., 2018; Hu et al., 2020). Because of the relatively stable enzyme activity of LDH, the LDH release level was often used as an indicator to detect gasdermin family dependent pyroptosis. Previous studies have shown that the expression of the gasdermin family proteins may execute pyroptosis, which can be induced by some chemotherapy drugs. In response to chemotherapy drug exposure, high expression levels of GSDME resulted in pyroptosis, whereas low levels of GSDME resulted in apoptosis (Stephen and Edward, 2017), indicating that the progression of pyroptosis may be faster than apoptosis in cancer cells (Shi et al., 2015). Although GSDME can regulate the conversion between apoptosis and pyroptosis, the molecular mechanism remains unknown.

Herein, our preliminary data showed that CBD induced caspase-3/GSDME mediated pyroptosis in HCC cells. Additionally, our results demonstrated that CBD induced integrative stress response (ISR)-dependent ATF4 activation, which contributed to GSDME-dependent pyroptosis. This supports a novel viewpoint to reveal the mechanism for CBD induced cell death, and we aimed to generate a new therapeutic strategy to evaluate the sensitivity and thereby modulate the effects of CBD therapy.

## Materials and Methods

### Cell Lines and Cell Culture

The HepG2, HUH7, HCCLM3, MHCC97H, and HEK293T cell lines were obtained from the Cell Bank of the Chinese Academy of Sciences (Shanghai, China). HepG2, HUH7, HCCLM3, MHCC97H, and HEK293T cells were cultured in DMEM medium supplemented with 10% fetal bovine serum and antibiotics. The cells were incubated at 37°C in a humidified incubator with 5% CO_2_. When the cultures reach approximately 50–70% confluence, the cells were treated with various concentrations of drugs. Dimethyl sulfoxide (DMSO) was used as vehicle control. All cell lines were mycoplasma free and authenticated by the Cell Bank of the Chinese Academy of Sciences.

### Reagents and Antibodies

Horseradish peroxidase (HRP)-conjugated anti-rabbit, anti-mouse immunoglobulin G, penicillin–streptomycin solution, Bradford protein assay kit, Cell counting kit-8 (CCK-8 kit), mitochondria membrane potential assay kit (JC-1), and LDH Release Assay Kit were obtained from Beyotime (Shanghai, China). Annexin V–PE/7AAD apoptosis detection kit was purchased from MULTI SCIENCES. Giemsa and crystal violet were purchased from Solarbio Bioscience and Technology (Shanghai, China). Trypan blue was obtained from Life Technologies (Carlsbad, CA, United States). BCA protein assay kit and Pierce ECL western blotting substrate were obtained from Thermo Fisher Scientific (MA, United States). Intact cellular oxygen consumption rate (OCR) and extracellular acidification rate (ECAR) assay kits were purchased from Seahorse Bioscience Company (North Billerica, MA, United States). Glucose was obtained from Sigma (St. Louis, MO, United States). L-glutamine was bought from Sangon Biotech (Shanghai, China). Puromycin was obtained from Amresco (WA, United States). Protease (Complete Mini) and phosphatase (PhosphoSTOP) inhibitor cocktail tablets were purchased from Roche Applied Science (Indianapolis, IN, United States). 2nbdg, EVAD, and ISRIB were gained from Selleck chemistry (TX, United States). Antibody information is shown in Supplementary Table 1.

### Cytotoxic and Anti-Proliferation Assays

To determine the inhibitory concentration (IC_50_) values of compounds, HepG2, HUH7, HCCLM3, and MHCC97H cells were seeded in 96-well plates at a density of 1 × 10^4^ cells/well and incubated overnight at 37°C with 5% CO_2_. Cells were then treated with different concentrations (0, 2.5, 5, 10, 20, 40, 80, or 160 μM) of CBD or DMSO (vehicle control) for 24 h, followed by CBD incubation with CCK-8 for another 2 h at 37°C. The absorbance at 450 nm was measured using a Varioskan Flash microplate reader (Thermo Scientific, Waltham, MA, United States). Each assay was performed in triplicate, and data was derived from at least three independent experiments.

The effects of CBD on the proliferation of HepG2, HUH7, and MHCC97H cell lines were evaluated using RTCA (Real Time CelI AnaIysis). RTCA instruments use gold biosensors embedded in the bottom of specialized microplate wells (Agilent E-Plates) to non-invasively monitor cell status including cell number. RTCA instruments monitored cell growth by detecting instantaneous impedance through the gold biosensors. The biosensors detect cellular impedance as cells adhere to and proliferate on the E-Plates, providing an extremely sensitive readout of cell number in real time. The impedance caused by adherent cells is reported using a unitless parameter called cell index (CI), where: CI = [(Impedance at n) − (Impedance without cells)]/(Nominal impedance constant). The larger CI value means the more cell number. Briefly, HepG2, HUH7, and MHCC97H cells were seeded into E-plate test plate at a density of 1 × 10^4^ cells/well and incubated overnight at 37°C with 5% CO_2_. After overnight detection, cells were treated with gradient concentrations (0, 20, and 40 μM) of CBD and the real-time dynamic detection was continued for 4 days to obtain the proliferation curve.

### Colony Formation Assay

Both HepG2, HUH7, and MHCC97H cells were counted and a total of 1,000 cells per well were seeded evenly into six-well plates and incubated at 37°C for 7–10 days in a humidified incubator with 5% CO_2_. After treatment with gradient concentrations (0, 20, and 40 μM) of CBD for another 2–4 days, cells were washed with pre-warmed PBS three times, fixed with 4% PFA, and stained with Giemsa solution for 15 min. Colonies were counted by two independent investigators.

### Xenograft Tumor Assay

Female athymic nude mice were purchased from Shanghai Laboratory Animal Center, CAS (Shanghai, China), and housed in a specific pathogen-free (SPF) environment. For *in vivo* tumorigenesis analysis, nude mice at the age of 5 weeks were injected subcutaneously in the left flanks with 1 × 10^7^ of HepG2 cells in 0.1 ml serum-free PBS and then mixed with 0.1 ml matrigel matrix. When the tumor volume has reached approximately 200 mm^3^, the mice were randomly sorted into two groups (*n* = 4/each group). The mice were gavaged with CBD suspension (40 mg/kg), once a day, for two consecutive weeks. At the same time, the control group was injected with the same volume of castor oil. The percentages of growth inhibition were defined as the ratio of tumor weight to that in the vehicle control. Tumor dimensions were determined using calipers, and the tumor volume (mm^3^) was calculated using the following formula: volume = length × (width)^2^/2. The mice were sacrificed, and the tumors were harvested and weighted. All animal studies were performed with a protocol approved by the Institutional Animal Care and Use Committee of Wenzhou Medical University.

### FACS Analysis for Cell Pyroptosis

For pyroptosis analysis, the HepG2, HUH7, and MHCC97H cells were treated with gradient concentrations of CBD (0, 20, and 40 μM) for 24 h, and cells were then collected and incubated with Annexin V-PE/7AAD in the dark at room temperature for 20 min, according to the manufacturer’s protocol. Thereafter, cell samples were analyzed immediately using a BD Accuri^TM^ C6 flow cytometer (BD, Franklin Lakes, NJ, United States).

### LDH Release Assay

To determine the LDH release caused by CBD, HepG2, HUH7, and MHCC97H cells were seeded in 96-well plates at a density of 2 × 10^4^ cells/well and incubated overnight at 37°C with 5% CO2. Cells were then treated with different concentrations (0, 20, and 40 μM) of CBD or DMSO (vehicle control) for 24 h. After reaching the predetermined time, the medium was collected and then centrifuged at 400 *g* for 5 min. Next, we detected the LDH release according to the manufacturer’s instructions.

### Western Blot Analysis

The ovarian cancer samples were washed three times with ice-cold PBS and homogenized using a homogenizer (Kinematica AG, Luzern, Switzerland) in 1.5 ml tissue RIPA lysis buffer (50 mM Tris–HCl, pH 7.4, 1.0% Triton X-100, 1% sodium deoxycholate, 0.1% SDS, and 150 mM NaCl) supplemented with protease inhibitor cocktail tablet, NaF (1 mM), and Na_3_VO_4_ (1 mM). Tissue homogenates were cleared by centrifugation at 13,000 rpm for 25 min at 4°C, and the supernatants were collected in clean microcentrifuge tubes on ice. A similar procedure was used to prepare cell extracts from cells. Briefly, HepG2, HUH7, and MHCC97H cells were washed with ice-cold PBS and lysed in RIPA lysis buffer supplemented with protease and phosphatase inhibitors on ice for 20 min, followed by centrifugation at 13,000 rpm for 30 min at 4°C, and the supernatants were collected. Protein concentrations of the tissue homogenates or cell extracts were determined using the Pierce BCA protein assay kit. Tissue or cell extracts equivalent to 20 μg total protein were resolved in 10% SDS-PAGE gels followed by electrophoretic transfer onto PVDF membrane (0.22 μM, Bio-Rad, Hercules, CA, United States) in Tris–glycine buffer. Blots were blocked at room temperature for 1.5 h in 5% non-fat milk in Tris-buffered saline (TBS)–Tween (TBS-T) on a shaker and then incubated with the primary antibodies in 5% non-fat milk TBS-T overnight at 4°C. The membrane was washed in TBS-T for at least 3 × 10 min and then incubated with horseradish peroxidase (HRP)-conjugated anti-rabbit or anti-mouse immunoglobulin G at room temperature for 1 h with gentle shaking. Immunoreactive proteins were detected by ECL reagent according to the manufacturer’s protocol (Biyetime biotechnology).

### RNA Interfere

Plasmids including pPLK/GFP + Puro-GSDME and pPLK/GFP + Puro-IGFBP1 shRNA were purchased from PPL (Public Protein/Plasmids Library). HEK293T cells were used for lentivirus package. HepG2 and MHCC97H cells were infected with packaged lentivirus, and successfully transfected cells were selected by puromycin (2 μg/ml). Western blot was performed to confirm the efficiency of GSDME and IGFBP1 depletion.

### RNA-Sequence Analysis

RNA-seq data was generated by Novogene. The CBD (40 μM) exposed HepG2 and MHCC97H cells were collected after 24 h, and then cells were washed by PBS for three times and then lysed by Trizol at 4°C for 10 min. Next, samples were sent to Novogene on dry ice. A total amount of 3 μg RNA per sample was used as input material for the RNA sample preparations. Sequencing libraries were generated using NEBNext Ultra^TM^ RNA Library Prep it for Illumina (NEB, United States) following the manufacturer’s recommendations, and index codes were added to attribute sequences to each sample. The clustering of the index-coded samples was performed on a cBot Cluster Generation System using TruSeq PE Cluster Kit v3-cBot-HS (Illumia) according to the manufacturer’s instructions. After cluster generation, the library preparations were sequenced on an Illumina platform and 125 bp/150 bp paired-end reads were generated. In the low-input protocol, RNA was purified using the RNeasy micro kit (Qiagen) to obtain 1–10 ng per pool. First-strand cDNA synthesis and amplification were performed using SMARTer Ultra-Low-Input RNA amplification kit v3 (Clontech). Single-end libraries were prepared using NEBNext Ultra DNA library prep for Illumina and sequenced on a NextSeq 500 instrument, yielding ∼11 million reads (range 8–13 million) of 75 bp in length. Low-quality ends and adaptor sequences were trimmed from the Illumina reads with FastX 0.0.13 and cutadapt 1.7.1. Using FastX and ShortRead 1.16.3, we subsequently filtered out short reads (length < 35 bp), poly(A) reads (where > 90% of the bases are adenine), ambiguous reads (containing Ns), and low-quality reads (where > 50% of the bases have quality < Q25). Heatmaps were generated using the R package pheatmap (version 1.0.8). The input values were log transformed FPKM or fold-change. Heatmaps were centered and scaled in the row direction. Euclidean distance and complete method were used for hierarchical clustering.

### XFe96 Extracellular Flux Analyzer Experiments

The intact cellular oxygen consumption rate (OCR) and the extracellular acidification rate (ECAR) in compound treated HepG2, HUH7, and MHCC97H cells were measured using a Seahorse XFe-96 Extracellular Flux Analyzer (Seahorse Bioscience, North Billerica, MA, United States) as described previously. Briefly, 80 μl single-cell suspensions of HepG2, HUH7, and MHCC97H cells were seeded in XFe-96 cell culture microplates (Seahorse Bioscience) at a cellular density of 25,000, 20,000 and 20,000 cells, respectively. The next day, the HepG2, HUH7, and MHCC97H cells were treated with gradient concentrations of CBD (0, 20, and 40 μM) for 4 h. For OCR determinations, cells were incubated in base assay medium (according to manufacturer’s instructions) supplemented with 2 mM glutamine, 10 mM glucose, and 1 mM pyruvate for 1 h, prior to the measurements using the XF Cell Mito Stress Kit (Seahorse Bioscience). The final concentrations of oligomycin, FCCP, and rotenone were 0.1 μM. For glycolytic metabolism measurements, cells were incubated in basal media prior to injections using the Glycolytic Test kit (Seahorse Bioscience). Results were obtained from three independent experiments, each with eight replicates of each group of cells. At the end of each assay, a BCA protein assay kit was used to determine and normalize the protein concentrations, according to the manufacturer’s instructions.

### FACS Analysis for Mitochondrial Membrane Potential

For mitochondria membrane potential analysis, the HepG2, HUH7, and MHCC97H cells were treated with gradient concentrations (0, 20, and 40 μM) of CBD for 24 h, and cells were then collected and incubated with JC-1 according to the manufacturer’s instructions in the dark at 37°C for 20 min. Thereafter, cell samples were analyzed immediately using a BD Accuri^TM^ C6 flow cytometer (BD, Franklin Lakes, NJ, United States).

### FACS Analysis for Glucose Uptake by 2-NBDG

For glucose uptake analysis, the HepG2 and MHCC97H cells were treated with gradient concentrations (0, 20, and 40 μM) of CBD for 24 h, and then cells were collected and incubated with 2-NBDG according to the manufacturer’s instructions in the dark at 37°C for 20 min. Thereafter, cell samples were analyzed immediately using a BD AccuriTM C6 flow cytometer (BD, Franklin Lakes, NJ, United States).

### Statistical Analysis

All statistical analyses were performed with the SPSS 16.0 statistical software package (SPSS Standard version 16.0, SPSS Inc., Chicago, IL, United States). Data are shown as the mean ± SD from at least three independent experiments. Groups of two were analyzed with two-tailed Student’s *t*-test, groups greater than two with a single variable were compared using one-way ANOVA analysis with Tukey post hoc test, *p < 0.05, **p < 0.01, ***p < 0.001, the n.s. represent no significance and *p* < 0.05 was considered statistically significant.

## Results

### CBD Inhibits HCC Cell Growth *in vivo* and *in vitro*

**FIGURE 1:**
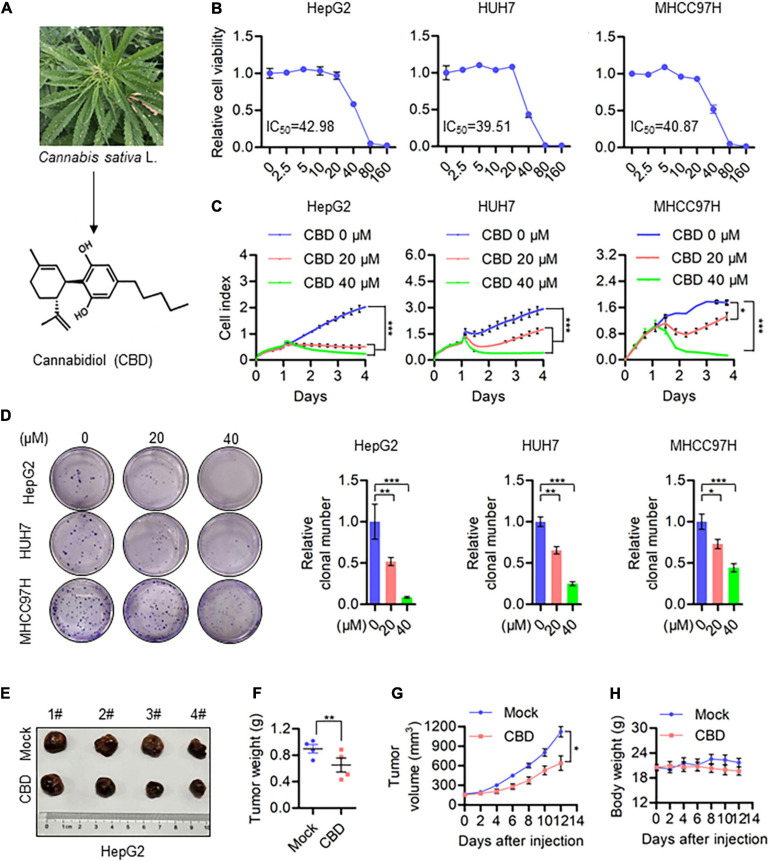
CBD inhibits HCC cell growth *in vivo* and *in vitro*. **(A)** Chemical structural formula of CBD. **(B)** These HCC cell lines HepG2, HUH7, and MHCC97H were treated with a gradient concentration of CBD for 24 h. Relative cell viability was analyzed by CCK-8 assay. **(C)** Cell proliferation of CBD treated HepG2, HUH7, and MHCC97H cells was determined by RTCA (Real Time CelI AnaIysis) according to the manufacturer’s instructions. **(D)** Representative images of colony formation assay were measured after HepG2, HUH7, and MHCC97H colony initiation being treated to different concentration of CBD for 2 days. The graphs represent the mean ± SD. **(E)** Representative dissected tumors of physiological mock group and CBD group derived from HepG2 cells were shown after the mice were sacrificed. **(F)** Changes in tumor weight of mice treated with castor oil or CBD. Data are presented as mean ± SEM (*n* = 4). **(G)** Tumor volume in each mouse was monitored every 2 days. Data are presented as mean ± SEM (*n* = 4). **(H)** Body weight measurements of mice treated with castor oil and CBD. Data are presented as mean ± SEM (*n* = 4). The meaning of ‘*‘ is statistically significant. *p < 0.05, **p < 0.01, ***p < 0.001.

To evaluate the anti-cancer activity of CBD which derived from Cannabis sativa L in HCC (Figure 1A), we investigated the effect of CBD on cell viability in a selection of HCC cells (HepG2, HUH7, MHCC97H, and HCCLM3). It was shown that cell viability decreased in a dose-dependent manner (Figure 1B and Supplementary Figure 1A). The IC_50_ was 40 μM in HepG2, HUH7, and MHCC97H cells, which was lower than the 53.8 μM determined for HCCLM3 cells. To further confirm the tumor-suppressive activity of CBD, cell proliferation and colony formation assays were performed. It was found that the number of cells was significantly reduced upon CBD exposure (Figures 1C,D). Furthermore, similar anti-tumor effects were observed for CBD in a nude mouse xenograft model, where tumor growth was inhibited (Figure 1E). In addition, it was found that tumor volume and weight were significantly reduced after CBD administration (Figures 1F,G). Importantly, treatment with CBD was well tolerated without notable body weight loss, which highlights the promising anti-cancer activity of CBD in the treatment of HCC (Figure 1H). These data indicate that CBD could suppress HCC cell growth.

### CBD Induces Pyroptosis in HCC Cells

**FIGURE 2:**
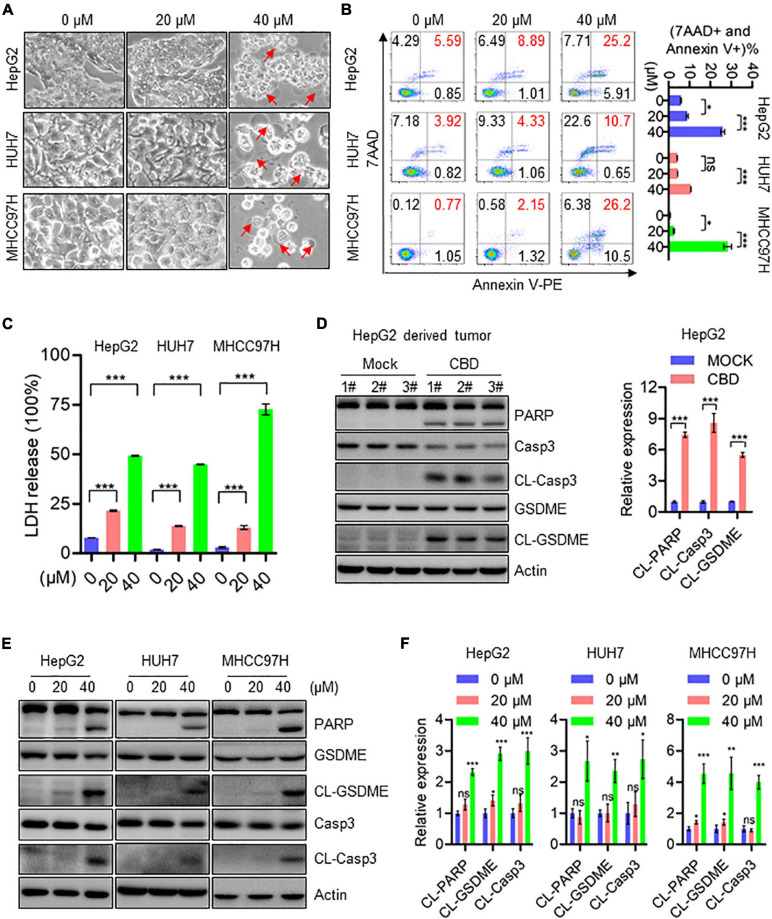
CBD induces cell pyroptosis in HCC cells. **(A)** Representative morphological changes of HepG2, HUH7, and MHCC97H cells in response to different concentrations of CBD. **(B)** Flow cytometry analysis of cell apoptosis after the HepG2, HUH7, and MHCC97H cells treated with different concentration of CBD for 24 h. Cells were collected and stained with Annexin V-PE/7AAD. Data are presented as mean ± SD. **(C)** LDH release assays were performed on CBD exposed HepG2, HUH7, and MHCC97H cells for 24 h. **(D)** Western blot analysis of PARP, Caspase-3, CL-Caspase-3, GSDME, and CL-GSDME in CBD treated tumor tissues derived from HepG2 cells. Actin was used as a loading control. The graphs represent the mean ± SEM. **(E,F)** Western blot analysis of PARP, Caspase-3, CL-Caspase-3, GSDME, and CL-GSDME in CBD treated HepG2, HUH7, and MHCC97H cells after 24 h, and actin was used as a loading control. The graphs represent the mean ± SD. The meaning of ‘*‘ is statistically significant. *p < 0.05, **p < 0.01, ***p < 0.001, n.s. represents no significance.

Subsequently, the effects of CBD on programmed cell death (PCD) in HCCs were determined. Interestingly, pyroptosis rather than apoptosis was seen in HepG2, HUH7, and MHCC97H cells, and the underlying mechanism of CBD induced pyroptosis has not been reported. Balloon-like bubbles (characteristic of pyroptotic cell morphology) were also noted in CBD treated HepG2, HUH7, and MHCC97H cells, which were distinct from classic apoptotic blebbing (Figure 2A). Moreover, flow cytometry analysis showed that CBD-induced cell death manifested as Annexin V and 7AAD double-positive cells (Figure 2B). It was also found that LDH release increased in CBD treated HepG2, HUH7, and MHCC97H cells, which indicated that the structural integrity of the cell membrane had been damaged and the contents leaked (Figure 2C). Consistently, increased cleavage of caspase 3, PARP, and GSDME was detected in CBD treated HCC cells and tumor sections (Figures 2D–F). Meanwhile, we also found that GSDMD was not significantly cleaved in response to CBD treatment (Supplementary Figure 2E). These observations suggest that CBD could induce pyroptosis in HCC cells.

### Caspase3/GSDME Axis Is Indispensable for CBD-Induced Pyroptosis

**FIGURE 3:**
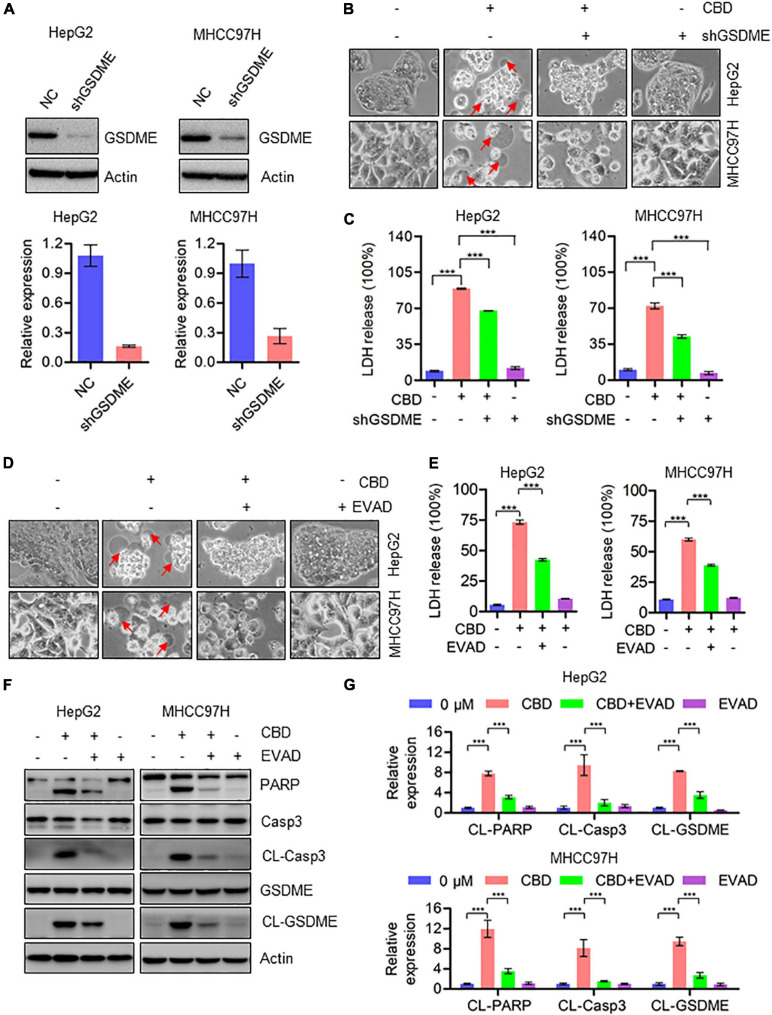
Activation of caspase3/GSDME axis is essential for CBD-induced pyroptosis. **(A)** Western blot was performed to detect the efficiency of GSDME knockdown in HepG2 and MHCC97H cells. Actin was used as a loading control. The graphs represent the mean ± SD. **(B)** Representative morphological changes in control and GSDME knockdown HepG2 and MHCC97H cells after CBD exposure. **(C)** LDH release assays were performed on control and GSDME knockdown HepG2 and MHCC97H cells after CBD administration. **(D)** Representative morphological changes in CBD-treated HepG2 and MHCC97H cells with or without EVAD. **(E)** LDH release assays were performed on CBD-exposed HepG2 and MHCC97H cells in the presence or absence of EVAD. **(F,G)** Western blot analysis of PARP, Caspase-3, CL-Caspase-3, GSDME, and CL-GSDME in CBD (40 μM) treated HepG2 and MHCC97H cells with or without EVAD after 24 h, and actin was used as a loading control. The graphs represent the mean ± SD. The meaning of ‘*‘ is statistically significant. ***p < 0.001.

Since CBD could trigger the cleavage of GSDME, we speculated whether cell pyroptosis depends on a GSDME-mediated cascade. To address this, GSDME was depleted in HepG2 and MHHC97H cells (Figure 3A). CBD treatment of control and GSDME knockdown cell lines showed that depletion of GSDME could significantly inhibit the appearance of the morphological characteristics of pyroptosis (Figure 3B). Consistently, GSDME downregulation can also reduce the high release of LDH induced by CBD in HepG2 and MHHC97H cells (Figure 3C). In addition, we observed that there was a reduction of plasma membrane bubbles (Figure 3D) and LDH release (Figure 3E) after co-application of CBD and caspase3 inhibitors E-VAD, compared to CBD treatment alone in HepG2 and MHHC97H cells. Finally, it was observed that the activation of caspase 3, PARP, and GSDME was reduced after EVAD treatment in HepG2 and MHHC97H cells (Figures 3F,G). These observations collectively confirmed that CBD-induced pyroptosis in HCC cells was dependent on activation of the caspase3/GSDME pathway.

### CBD Triggers Mitochondrial Dysfunction by Suppressing the Transcription of Mitochondrial Component Molecules

**FIGURE 4:**
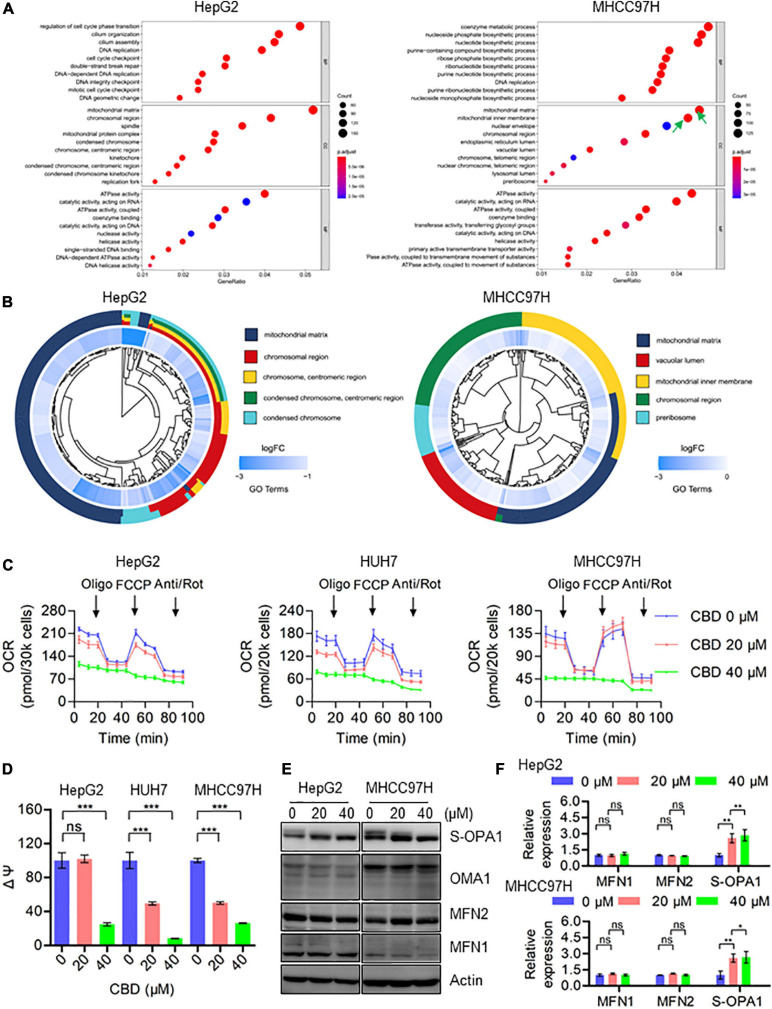
CBD triggers mitochondria dysfunction by suppressing the transcription of mitochondrial component proteins. **(A)** The enriched GO terms in the biological process (BP), cellular component (CC), and molecular function (MF) in HepG2 and MHCC97H cells after CBD treatment for 24 h. **(B)** We selected the significantly changed genes of cellular component (CC) to conduct the functional analysis and found that the **DEGs (differentially expressed genes)** were significantly enriched in mitochondria-related items in HepG2 and MHCC97H cells after CBD treatment for 24 h. **(C)** The intact cellular oxygen consumption rate (OCR) of CBD treated HepG2, HUH7, and MHCC97H cells in the indicated conditions was measured in real time using the Seahorse XF96 Extracellular Flux Analyzer. Basal OCR was measured at three time points, followed by sequential injection of the ATP synthase inhibitor oligomycin (1 μM), the uncoupler FCCP (1 μM), the complex I inhibitor rotenone (1 μM), and the complex III inhibitor antimycin A (1 μM). **(D)** Mitochondrial membrane potential was detected by JC-1 staining in CBD-treated HepG2, HUH7, and MHCC97H cells. **(E,F)** Western blot analysis of OPA1, MFN1, MFN2, and OMA1 in CBD treated HepG2 and MHCC97H cells after 24 h. Actin was used as a loading control. The graphs represent the mean ± SD. The meaning of ‘*‘ is statistically significant. *p < 0.05, **p < 0.01, ***p < 0.001, n.s. represents no significance.

To investigate the molecular mechanisms underlying CBD induced cell pyroptosis, RNA sequencing was performed on HepG2 and MHCC97H cells, which have been treated with CBD for 24 h. A GO enrichment analysis was conducted by cluster Profiler package and a GO dot plot drawn. A circle diagram of GO was further obtained using the GO plot package. Interestingly, the results confirmed that the differentially expressed genes were significantly enriched in the GO cellular component (CC) of the mitochondrial matrix, mitochondrial inner membrane, and mitochondrial protein complex (Figure 2A) in both cell lines exposed to CBD. Furthermore, the five most significant cellular component (CC) terms related to the differentially expressed genes of CBD-treated HepG2 and MHCC97H cells were depicted (Figure 2B). The circle diagram showed that CBD mainly impacts on the terms of mitochondrial matrix and chromosomal region, and the mitochondrial-related genes occupied half of the ratio. Consistent with these results, it was also observed that there was a decreased transcription of mitochondrial respiratory chain complex subunits (Supplementary Figure 3A), indicating that the mitochondrial component proteins may play roles in CBD regulation. Subsequently, the overall mitochondrial oxygen consumption rate (OCR) was measured using a Seahorse XF96 flux, a measurement of mitochondrial OXPHOS. OCR was dramatically decreased after CBD treated in HCC cells (HepG2, HUH7, and MHCC97H) (Figure 4C), accompanied by a significant reduction of OCR indices as basal respiration, ATP production, and maximal respiration (Supplementary Figure 3B). Based on mitochondrial bioenergetics, mitochondrial membrane depolarization and loss of membrane potential was observed (Figure 4D). Moreover, we verified that OPA1, a crucial mitochondrial dynamics associated protein, was cleaved in response to CBD exposure. However, there was no effect on other dynamics associated proteins such as MFN1 and MFN2 (Figures 4E,F). In summary, CBD could trigger mitochondrial stress and dysfunction by restraining the transcription of mitochondrial component proteins.

### Activation of ATF4–CHOP Axis Triggers GSDME-Mediated Pyroptosis in Response to CBD

**FIGURE 5:**
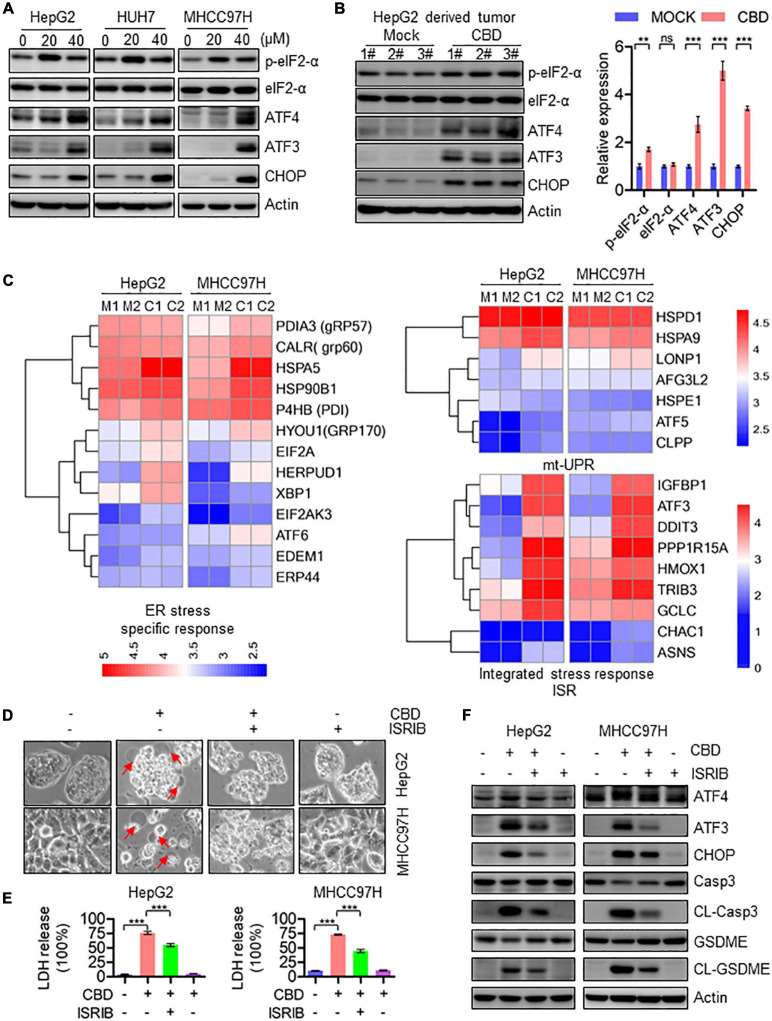
Activation of ATF4/CHOP pathway triggers the GSDME-mediated pyroptosis in response to CBD treatment. **(A)** Western blot analysis of p-eIF2-α, eIF2-α, ATF4, ATF3, and CHOP in CBD treated HepG2, HUH7, and MHCC97H cells after 24 h. Actin was used as a loading control. **(B)** Western blot analysis of p-eIF2-α, eIF2-α, ATF4, ATF3, and CHOP in CBD administrated tumor tissues derived from HepG2 cells. Actin was used as a loading control. The graphs represent the mean ± SEM. **(C)** Heat map analysis from RNA-sequence data in CBD exposed HepG2 and MHCC97H cells for 24 h. **(D)** Representative morphological changes in CBD-treated HepG2 and MHCC97H cells in the presence or absence of ISRIB. **(E)** LDH release was performed on CBD-exposed HepG2 and MHCC97H cells with or without ISRIB. **(F)** Caspase-3, CL-Caspase-3, GSDME, CL-GSDME, ATF4, ATF3, and CHOP were detected by Western blotting in CBD (40 μM) administrated HepG2 and MHCC97H cells in the presence or absence of ISRIB after 24 h. **p < 0.01, ***p < 0.001, n.s. represents no significance.

To further elucidate the correlation between mitochondrial dysfunction and cell pyroptosis, RNA-sequence data was analyzed. It was found that the pro-apoptotic factors of the BCL-2 family such as BAX, BAK, and BAD, which are upstream of caspase-3, were dramatically induced in CBD-treated HCC cells, indicating that CBD could activate caspase-3 dependent factors (Supplementary Figure 4A). We further detected the ATF4/CHOP axis, which has been proven to be an upstream transcription factor of the BCL-2 family (Mariano et al., 1996; Tsukano et al., 2010; Lurlaro and Muñoz-Pinedo, 2016). It was observed that p-eIF2-α, ATF4, ATF3, and CHOP were significantly induced both *in vivo* and *in vitro* after CBD administration (Figures 5A,B and Supplementary Figures 4B,C). Furthermore, to confirm the upstream regulator of the ATF4/CHOP pathway, the changes among ER stress transcripts, mt-UPR and ISR, were analyzed from the RNA-sequence data after CBD exposure. The data showed that the ER stress-related genes were slightly increased, while no significant changes in mt-UPR-related genes were seen. However, an appreciable induction of ISR targeted genes was seen after CBD administration (Figure 5C), suggesting that CBD could cause the activation of ATF4 in an ISR-dependent pathway. In order to further confirm the mechanism, CBD was added along with ISRIB (an ISR inhibitor) (Sidrauski et al., 2015). This resulted in the downregulation of pyroptotic morphologies and LDH release triggered by CBD with or without ISRIB in HepG2 and MHCC97H cells (Figures 5D,E). Moreover, similar results were observed by Western blotting data derived from HepG2 and MHCC97H cells exposed to CBD in the presence or absence of ISRIB (Figure 5F and Supplementary Figure 4D). These observations demonstrated that CBD exposure could lead to HCC cell pyroptosis as a result of inducing ISR-dependent ATF4 activation.

### CBD, Which Inhibits Cell Glycolysis Through Repressing AKT–GSK3β Axis, Is Dependent on ATF4 Activation

**FIGURE 6:**
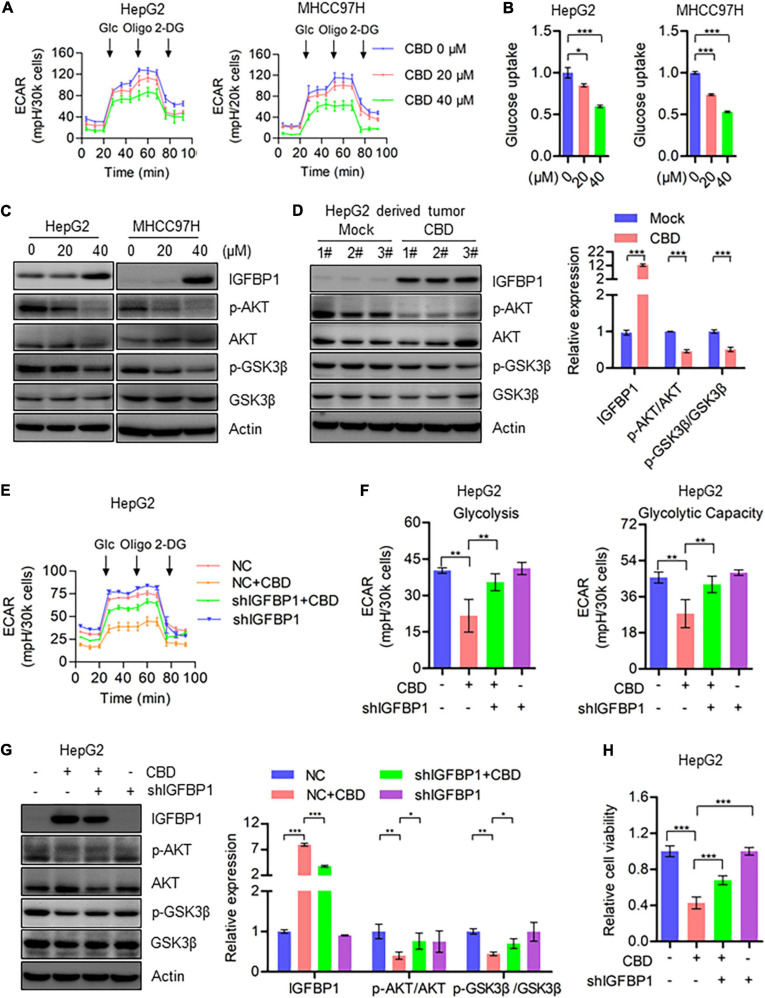
CBD inhibits cell glycolysis through repressing AKT/GSK3β axis. **(A)** Extracellular acidification rate (ECAR) of CBD treated HepG2 and MHCC97H cells in the indicated conditions was measured in real time using the Seahorse XF96 Extracellular Flux Analyzer. Basal ECAR was measured at three time points, followed by sequential injection of glucose (10 mM), oligomycin (1 μM), and 2-DG (100 mM). Data are presented as mean ± SD. **(B)** Glucose uptake was detected by 2-NBDG staining in CBD-treated HepG2, HUH7, and MHCC97H cells. **(C)** Western blot analysis of IGFBP1, p-AKT, AKT, p-GSK3β, and GSK3β in CBD treated HepG2 and MHCC97H cells after 24 h. Actin was used as a loading control. **(D)** Western blot analysis of IGFBP1, p-AKT, AKT, p-GSK3β, and GSK3β in CBD administrated tumor tissues derived from HepG2 cells. Actin was used as a loading control. The graphs represent the mean ± SEM. **(E,F)** Extracellular acidification rate (ECAR) was measured in control and IGFBP1 knockdown HepG2 and MHCC97H cells after CBD exposure. **(G)** Western blotting analysis of IGFBP1, p-AKT, AKT, p-GSK3β, and GSK3β in control and IGFBP1 knockdown HepG2 cells after CBD (40 μM) administration for 24 h. **(H)** Cell viability was determined in control and IGFBP1 depletion HepG2 cells in the presence of CBD for 24 h. The meaning of ‘*‘ is statistically significant. *p < 0.05, **p < 0.01, ***p < 0.001.

Based on the heat map data, it was indicated that IGFBP1 was a downstream target of ATF4, activated by ISR (Figure 5C). Therefore, IGFBP1 expression was assessed after CBD treatment with or without ISRIB. The Western blot data showed that IGFBP1 was downregulated when CBD and ISRIB were added together, compared to CBD treatment alone (Supplementary Figure 5A). IGFBP1 has been shown to be a metabolic regulator under stress conditions in some cancer types (Inagaki et al., 2008; Seferovic et al., 2009), and enhanced glycolytic capacity is considered to be a hallmark of cancer. It was therefore considered that CBD could regulate glycolysis as a means to control cell survival under stress. Interestingly, it was observed that CBD could depress the overall aerobic glycolytic rate in HepG2 and MHCC97H cells (Figure 6A). Conversely, the basal glycolysis, glycolytic capacity, and glycolytic reverse were decreased in CBD-treated HepG2 and MHCC97H cells (Supplementary Figure 5B). Consistent with the data that showed that CBD suppressed ECAR in HCC cells, it was observed that glucose absorption in CBD-treated HepG2 and MHCC97H cells was inhibited (Figure 6B and Supplementary Figure 5C). Moreover, it was shown that CBD can repress the AKT/GSK3β axis accompanied by the induction of IGFBP1 *in vivo* and *in vitro* (Figures 6C,D). Consequently, it was suspected that CBD could depress cell glycolysis in the IGFBP1/AKT/GSK3β axis. Therefore, IGFBP1 stable knockdown HepG2 cells were constructed. It was found that the down-regulation of the overall aerobic glycolytic rate, basal glycolysis, and glycolytic capacity caused by CBD can be rescued by IGFBP1 depletion (Figures 6E,F). A similar result was seen by immune-blotting, where depletion of IGFBP1 can rescue the depression of AKT/GSK3β axis triggered by CBD (Figure 6G). Furthermore, it was found that IGFBP1-knockdown cells were less sensitive to CBD compared to the control cells (Figure 6H). These results revealed that CBD could inhibit glycolysis *via* an ISR-dependent ATF4/IGFBP1/AKT/GSK3β axis.

## Discussion

In this study, we have shown that CBD can effectively suppress HCC cell growth both *in vitro* and *in vivo*, which was similar to the anti-tumor activity of CBD observed in other cancer types (Hu et al., 2020). Importantly, it was shown that CBD triggers ISR-dependent ATF4 activation, which contributes to the induction of its target genes CHOP and IGFBP1. It was confirmed that induction of CHOP regulated the BCL-2 family to trigger caspase-3/GSDME dependent pyroptosis as well as depression of glycolysis by restraint of the AKT–GSK3β–IGFBP1 axis, suggesting a potential role of CBD in modulating cancer cell PCD and metabolism.

Previous studies have demonstrated that CBD treatment could effectively induce cell apoptosis in tumor cells (Sultan et al., 2018; Alharris et al., 2019; Jeong et al., 2019c; Kis et al., 2019), but whether CBD could induce pyroptosis in tumor cells remains unreported. One type of programmed cell death (PCD), pyroptosis, may provide a possible beneficial effect on tumor cell therapy (Shi et al., 2015; Wang et al., 2017). Moreover, pyroptosis is thought to be a kind of programmed necrosis mediated by gasdermin family proteins (Muller et al., 2019). Many chemotherapy drugs can induce pyroptosis through GSDME cleavage, one of the gasdermin family members (Rogers et al., 2017; Wang et al., 2017), while bacteria or LPS (Lipopolysaccharide) induces pyroptosis through GSDMD cleavage (He et al., 2015; Kayagaki et al., 2015; Shi et al., 2015). Based on the IC50 data, we treated the HCC cells with CBD (40 μM) in a time course to determine the initial time the cell death occurred. The data showed that CBD initial induced cell death at 18 h and significantly induced cell death at 24 h (Supplementary Figures 2F,G); therefore, we chose 24 h as the treatment time. In the present study, it was observed that GSDME was activated by CBD in HepG2, HUH7, and MHCC97H cells rather than GSDMD (Figure 2E and Supplementary Figure 2E). Meanwhile, it was observed that CBD induced LDH release and pyroptotic morphologies in these cells, indicated by cell swelling and accompanied by large bubbles blown from the plasma membrane (Figures 2A,C). However, this phenomenon was not observed in HCCLM3 cells after CBD treatment (Supplementary Figures 2A,B). It was also found that HCCLM3 was less sensitive to CBD treatment than the other three HCC cells (Supplementary Figure 2C). It is possible that this may be related to GSDME expression (Yang et al., 2020). Therefore, the basal expression of GSDME in LO2, HepG2, HUH7, HCCLM3, and MHCC97H cells was determined. Results in Supplementary Figure 2D showed that the expression of GSDME was significantly lower than in the other cells, indicating that the level of GSDME protein is positively correlated with the cell sensitivity to CBD exposure induced pyroptosis. Since it was indicated that CBD could induce pyroptosis in cells expressing high levels of GSDME, it was suspected that GSDME may be crucial for CBD induced pyroptosis. Therefore, GSDME stable knockdown cells were generated in HepG2 and MHCC97H cells (Figure 3A). Interestingly, it was observed that the LDH release and pyroptotic morphologies induced by CBD were weakened in GSDME-depleted HepG2 and MHCC97H cells (Figures 3B,C), suggesting that the CBD triggered cell pyroptosis was GSDME dependent. However, cleavage of caspase-3 and PARP is not affected in GSDME knockdown cells in responding to CBD treatment in HepG2 and MHCC97H cells (Supplementary Figure 6A). Consistently, flow cytometry data showed that depletion of GSDME do not rescue the cell death caused by CBD, but GSDME knockdown cells increase the proportion of Annexin V-PE single-positive cells and reduce the percentage of double-positive cells (Supplementary Figure 6B). These data indicated that the deficiency of GSDME may switch CBD-triggered cell death from pyroptosis to apoptosis (Zhang et al., 2020). Previous studies have demonstrated that GSDME-dependent pyroptosis can be activated by pro-apoptotic caspases, such as caspase-3^25^. Consistent with this finding, caspase-3 was also activated after CBD exposure (Figure 2E). Therefore, HepG2 and MHCC97H cells were exposed to CBD with or without the caspase-3 inhibitor EVAD. It was indeed found that EVAD could eliminate the cell pyroptosis triggered by CBD (Figures 3D–G). Combined with our previous findings, it can be concluded that CBD can induce pyroptosis *via* the caspase-3/GSDME pathway.

Meanwhile, recent research has shown that chemotherapy-induced pyroptosis is mediated by the BAK/BAX–caspase-3–GSDME pathway (Zhang et al., 2018), indicating that the pro-apoptotic factors of the BCL-2 family may be involved in cell pyroptosis. Moreover, Zhang et al. (2020) have demonstrated that mitochondrial dysfunction triggered caspase3–GSDME pathway activation can eventually lead to pyroptosis in response to Miltirone in HCC cells. There may therefore be a similar mechanism in CBD-induced pyroptosis. The RNA-sequence data showed that Bad, Bak, and Bax may be upstream of caspase-3/GSDME induced pyroptosis (Supplementary Figure 4A). Previous studies have indicated that ATF4/CHOP may be the crucial upstream transcription factor to regulate the BCL-2 family protein expression (Hu et al., 2019). It was shown here that the ATF4/ATF3/CHOP pathway was significantly induced both *in vivo* and *in vitro* after CBD treatment (Figures 5A,B and Supplementary Figure 4C). In addition, ATF4/CHOP has been reported to be triggered by ER stress (Han et al., 2013; Chen et al., 2014), mt-UPR (Horibe and Hoogenraad, 2007), and ISR (Harding et al., 2003; Guan et al., 2017; Quirós et al., 2017) and even in an mTOR-dependent way (Ben-Sahra et al., 2016). To confirm the upstream regulator of the ATF4/CHOP pathway, we analyzed the changes after CBD administration among ER stress, mt-UPR, and ISR from RNA-sequence data. Our data showed a significant induction in ISR target genes rather than ER stress and mt-UPR (Figure 5C). We further demonstrated the relationship between ATF4 and the ISR program by using ISRIB, an ISR inhibitor. Our data showed that the activation of the ATF4/CHOP axis and index of pyroptosis mediated by CBD were eliminated after ISRIB exposure (Figures 5D–F and Supplementary Figure 4D), suggesting that CBD-triggered cell pyroptosis may be mediated by ISR-dependent ATF4 activation. Meanwhile, previous research has demonstrated that CBD would trigger the gating of constitutively expressed TRPV channels to elicit influx of Ca^2+15^. In turn, sustained Ca^2+^ influx could induce ER dysfunction and consequent ISR. We found the changes about TRPV1, TRPV2, and TRPV4 were not consistent in HepG2 and MHCC97H cells, but the TRPV3 was induced significantly in these two cells. All the results indicated that TRPV3, rather than TRPV1 and TRPV2, may be a potential factor upstream in mediating activation of the ISR by CBD. Interestingly, TRPV3 channel was found to be presented in the mitochondrial region, indicating that TRPV3 may be involved in maintaining homeostasis of mitochondrial Ca^2+^ influx (Majhi et al., 2020). Furthermore, mitochondria are the major source of cellular ATP and crucial metabolites, so targeting mitochondrial OXPHOS is considered as a promising therapeutic strategy for cancer. Previous works have identified that CBD can lead to mitochondria stress (Jeong et al., 2019a,b), but the downstream targets of mitochondrial stress remain unknown. Interestingly, many drugs can cause mitochondrial stress and dysfunction, contributing to ISR-dependent ATF4 activation (Quirós et al., 2017; Fessler et al., 2020; Guo et al., 2020). Data herein showed that CBD can inhibit the transcription of mitochondrial component proteins, which may trigger an imbalance of mitochondrial homeostasis (Figures 4A,B). Moreover, the elimination of mitochondrial respiration, loss of membrane potential, and dynamic imbalance can also lead to mitochondrial stress and dysfunction contributing to ISR activation (Figures 4C–F). Additionally, the mitochondrial dysfunction was initiated after 4 h of CBD (40 μM) exposure, which was earlier than the ISR-induced pyroptosis (Figure 4C and Supplementary Figures 2F,G). Further work is needed to verify the potential mechanism of mitochondrial stress induced activation of ISR.

It is well known that the enhancement of glycolytic capacity is known as the “Warburg effect” and is considered to be a critical hallmark of cancer (Hanahan and Weinberg, 2011; Birsoy et al., 2014; Liberti and Locasale, 2016). Thus, multiple drugs targeting tumor glycolysis may be a potential therapeutic strategy. Our results demonstrated that the extracellular acidification rate (ECAR) was repressed by CBD in HCC cells (Figure 6A and Supplementary Figure 5B). Moreover, similar data was gained from glucose uptake assays in HCC cells (Figure 6B and Supplementary Figure 5C). However, the upstream components of glycolysis repression in CBD treated HCC cells remain unknown. Analysis of RNA-sequence data indicated that IGFBP1 was significantly induced by CBD administration (Figure 5C), which has been considered to be a glycolytic regulator *via* modulation of the PI3K/AKT or FAK/AKT pathway (Wheatcroft and Kearney, 2009; Man et al., 2018). Interestingly, it was observed that the inhibition of AKT/GSK3β was accompanied by IGFBP1 induction after CBD exposure (Figures 6C,D). Therefore, it was suspected that CBD can regulate cell glycolysis through the IGFBP1/AKT axis. To further understand the relationship between the IGFBP1 and cell glycolysis capacity, IGFBP1 stable knockdown HepG2 cells were constructed. The restrained glycolysis and the inhibited AKT/GSK3β axis were rescued by IGFBP1 depletion in HCC cells (Figures 6E–G), indicating that CBD can regulate glycolysis *via* modulation of the IGFBP1/AKT/GSK3β axis. Additionally, our data showed that IGFBP1 and ATF4 were both induced as the ISR-target gene in CBD-treated HCC cells (Figure 5C), consistent with data from the literature that the ISR-dependent ATF4 induction may be upstream of IGFBP1 (Marchand et al., 2006; Magne et al., 2011). Therefore, we hypothesized that CBD could depress glycolysis by inhibition of the AKT/GSK3β axis in an ATF4/IGFBP1 dependent way. We further detected the changes of IGFBP1 in CBD-treated cells in the presence or absence of ISRIB, an ATF4 inhibitor. It was found that the induction of IGFBP1 caused by CBD can be depressed after ISRIB treatment (Supplementary Figure 5A), indicating that IGFBP1 can be regulated by ATF4 in an ISR-dependent pathway.

**FIGURE 7:**
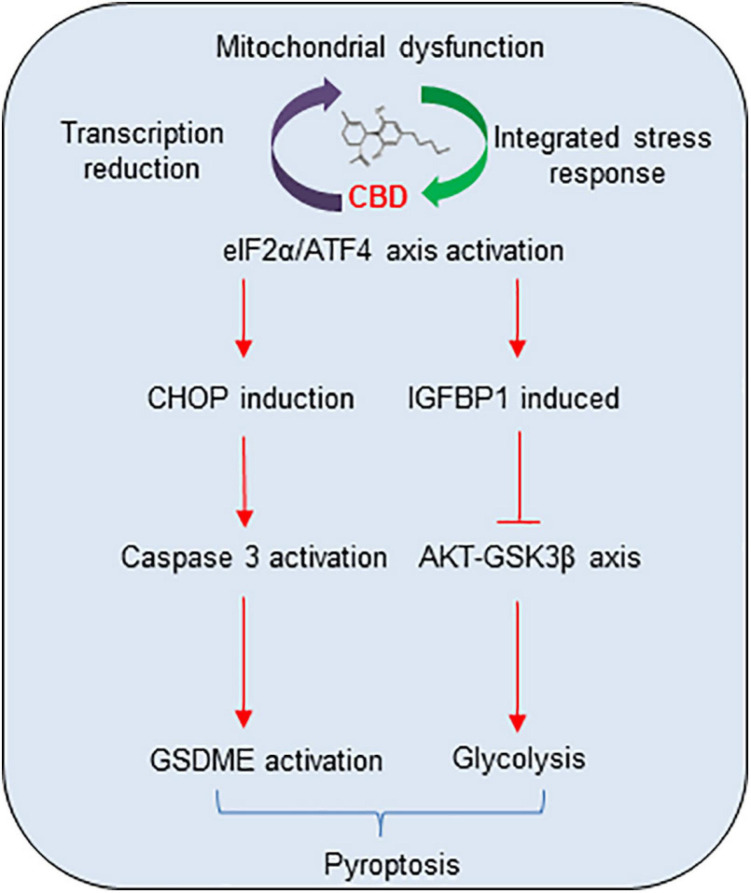
The proposal mechanistic model of CBD depresses HCC through modulating ISR-dependent pyroptosis and IGFBP1/AKT axis mediated glycolysis inhibition. CBD triggers accumulation of integrative stress response and mitochondrial stress, which subsequently induces ATF4 and its target genes CHOP and IGFBP1 activation. Then, increased CHOP promotes pro-apoptosis factor of BCL-2 family to trigger caspase-3/GSDME dependent pyroptosis and activates IGFBP1 to depress glycolysis by restraining the AKT/GSK3β axis.

In summary, a mechanistic model of CBD anti-tumor activity in HCC cell pyroptosis and growth was demonstrated (Figure 7). The cycle of accumulated mitochondria stress and ISR facilitates the ATF4/CHOP dependent pyroptosis and ATF4/IGFBP1 mediated glycolysis inhibition, which coordinates to suppress HCC growth. All the observations described herein reveal a novel mechanism of the anti-tumor activity of CBD in HCC cells, suggesting that CBD could be considered as a promising compound for HCC therapy.

## Data Availability Statement

The RNA-sequence data is available in the GEO database at https://www.ncbi.nlm.nih.gov/geo/query/acc.cgi?acc=GSE179661 under accession GSE179661.

## Ethics Statement

The animal study was reviewed and approved by Wenzhou Medical University.

## Author Contributions

LL, HX, and FS conceived the study. FS, HZ, NM, SW (7th author), WH, and WZ performed laboratory work. FS, SW (4th author), HH, and GJ analyzed the data. FS, LL, and HX wrote the manuscript. All authors read and approved the final manuscript.

## Conflict of Interest

The authors declare that the research was conducted in the absence of any commercial or financial relationships that could be construed as a potential conflict of interest.
