# Supplementary material for: A Novel Mechanism of Cannabidiol in Suppressing Hepatocellular Carcinoma by Inducing GSDME Dependent Pyroptosis
Source: Front Cell Dev Biol. 2021 Jul 19;9:697832. doi: 10.3389/fcell.2021.697832 (PMC8327166; doi:10.3389/fcell.2021.697832)
Supplement: Supplementary file 1 [file Table_1.pdf]

Supplemental table 1

Information list of antibodies used in this study.

Name	Cat No.	species	Company
GSDME	ab215191	Rabbit	Abcam
ATF3	A1852	Rabbit	ABclonal
β-Actin	ACO26	Rabbit	ABclonal
Cleaved- Caspase-3	A11021	Rabbit	ABclonal
ISR1	WH147926	Rabbit	ABclonal
OPA1	612607	Mouse	BD sciences
AKT	#9272	Rabbit	Cell Signaling Technology
Caspase-3	#9662	Rabbit	Cell Signaling Technology
eIF2α	#5324	Rabbit	Cell Signaling Technology
GSK3β	#12456	Rabbit	Cell Signaling Technology
p-AKT	#4060	Rabbit	Cell Signaling Technology
PARP	#9542	Rabbit	Cell Signaling Technology
p-eIF2α	#9721	Rabbit	Cell Signaling Technology
p-GSK3β	#5558	Rabbit	Cell Signaling Technology
ATF4	10835-1-AP	Rabbit	Protein technology Group
CHOP	15204-1-AP	Rabbit	Protein technology Group
IGFBP1	13981-1-AP	Rabbit	Protein technology Group
MFN1	13798-1-AP	Rabbit	Protein technology Group
MFN2	12186-1-AP	Rabbit	Protein technology Group
OMA1	SC-515788	Mouse	SantaCruz Biotechnology
