# Supplementary material for: A Novel Mechanism of Cannabidiol in Suppressing Hepatocellular Carcinoma by Inducing GSDME Dependent Pyroptosis
Source: Front Cell Dev Biol. 2021 Jul 19;9:697832. doi: 10.3389/fcell.2021.697832 (PMC8327166; doi:10.3389/fcell.2021.697832)
Supplement: Supplementary file 2 [file Data_Sheet_1.pdf]

Supplementary materials

A novel mechanism of Cannabidiol in suppressing hepatocellular carcinoma by inducing GSDME dependent pyroptosis

Fugen Shangguan[a,†], Hongfei Zhou[a, †], Nengfang Ma [b, †], Shanshan Wu[c], Huimin Huang[a], Guihua Jin[a], Shijia Wu[a], Weilong, Hong[a], Weiwei Zhuang[a], Hongping Xia[a, c,]*, Linhua Lan[a, *]

[a]Key Laboratory of Diagnosis and Treatment of Severe Hepato-Pancreatic Diseases of Zhejiang Province, the First Affiliated Hospital of Wenzhou Medical University, Wenzhou 325000, China.

[b]School of Life and Environmental Sciences, Wenzhou University, Wenzhou, China.

[c]Medical Research Center, The First Affliated Hospital of Wenzhou Medical University, Wenzhou, China

[d]Department of Pathology in the School of Basic Medical Sciences & The Affiliated Sir Run Run Hospital & State Key Laboratory of Reproductive Medicine & Key Laboratory of Antibody Technique of National Health Commission, Nanjing Medical University, Nanjing 211166, China.

[†]These authors contributed equally to this work.

***Corresponding to** **Dr. Linhua Lan**, E-mail address: paullee90@wmu.edu.cn; **Prof. Hongping Xia**, E-mail address: xiahongping@njmu.edu.cn.

Running title: Cannabidiol induces pyroptosis.

Materials and Methods

Cell lines and cell culture

The HepG2, HUH7, HCCLM3, and MHCC97H human hepatocellular carcinoma cell lines were obtained from the Cell Bank of the Chinese Academy of Sciences (Shanghai, China). HepG2, HUH7, HCCLM3, and MHCC97H cells were cultured in DMEM medium supplemented with 10% fetal bovine serum and antibiotics. The cells were incubated at 37°C in a humidified incubator with 5% CO_2. When the cultures reach approximately 50-70% confluence, the cells were treated with various concentrations of drugs. Dimethyl sulfoxide (DMSO) was used as vehicle control. All cell lines were mycoplasma free and authenticated by the Cell Bank of the Chinese Academy of Sciences.

Reagents and antibodies

Horseradish peroxidase (HRP)-conjugated anti-rabbit, Anti-mouse immunoglobulin G, Penicillin-Streptomycin Solution, Bradford protein assay kit, Cell counting kit-8 (CCK-8 kit), and Mitochondria membrane potential assay kit (JC-1) were obtained from Beyotime (Shanghai, China). BCA protein assay kit and Pierce ECL western blotting substrate were obtained from ThermoFisher Scientific (Massachusetts, USA). Intact cellular oxygen consumption rate (OCR) and extracellular acidification rate (ECAR) assay kits were purchased from Seahorse Bioscience Company (North Billerica, MA, USA). Glucose was obtained from Sigma (St. Louis, MO, USA). L-glutamine was bought from Sangon Biotech (Shanghai, China). Puromycin was obtained from Amresco (Washington, USA) Protease (Complete Mini) and phosphatase (PhosphoSTOP) inhibitor cocktail tablets were purchased from Roche Applied Science (Indianapolis, IN). 2nbdg, EVAD, and ISRIB were gained from Selleck chemistry

(Texas, USA). Detail information of antibodies were summarized in Table 1.

Cytotoxic assays

HCCLM3 cells were seeded in 96-well plates at a density of 1×10^4 cells/well and incubated overnight at 37 °C with 5% CO_2. Cells were then treated with different concentrations (0, 2.5, 5, 10, 20, 40, 80, or 160 μM) of CBD or DMSO (vehicle control) for 24 hr, followed by CBD incubating with CCK-8 for another 2 hr at 37°C. The absorbance at 450 nm was measured using a Varioskan Flash microplate reader (Thermo Scientific, Waltham, MA). Each assay was performed in triplicate and data was derived from at least three independent experiments.

To further determine whether HCCLM3 also showed lower sensibility than HepG2, HUH7, and MHCC97H cells, cells were treated with different concentrations (0, 0.625, 1.25, 2.5, 5, 10, 20, or 40 μM) of CBD or DMSO (vehicle control) for 48 hr, followed by CBD incubating with CCK-8 for another 2 hr at 37°C.

Western blot analysis

The ovarian cancer samples were washed three times with ice-cold PBS and homogenized using a homogenizer (Kinematica AG, Luzern, Switzerland) in 1.5 mL tissue RIPA lysis buffer (50 mM Tris-HCl, pH 7.4, 1.0% Triton X-100, 1% sodium deoxycholate, 0.1% SDS and 150 mM NaCl) supplemented with protease inhibitor cocktail tablet, NaF (1 mM) and Na_3VO_4 (1 mM). Tissue homogenates were cleared by centrifugation at 13,000 rpm for 25 min at 4°C, and the supernatants were collected in clean microcentrifuge tubes on ice. A similar procedure was used to prepare cell extracts from cells. Briefly, HepG2, HUH7, and MHCC97H cells were washed with ice-cold PBS and lysed in RIPA lysis buffer supplemented with protease and phosphatase inhibitors on ice for 20 min, followed by centrifugation at 13,000 rpm for 30 min at

4°C, and the supernatants were collected. Protein concentrations of the tissue homogenates or cell extracts were determined using the Pierce BCA protein assay kit. Tissue or cell extracts equivalent to 20 μg total protein were resolved in 10% SDS-PAGE gels followed by electrophoretic transfer onto PVDF membrane (0.22 μM, Bio-Rad, Hercules, CA) in Tris-glycine buffer. Blots were blocked at room temperature for 1.5 hr in 5% non-fat milk in Tris-buffered saline (TBS)-Tween (TBS-T) on a shaker and then incubated with the primary antibodies in 5% non-fat milk TBS-T overnight at 4°C. The membrane was washed in TBS-T for at least 3 × 10 min and then incubated with horseradish peroxidase (HRP)-conjugated anti-rabbit or anti-mouse immunoglobulin G at room temperature for 1 hr with gentle shaking. Immunoreactive proteins were detected by ECL reagent according to the manufacturer's protocol (Biyetime biotechnology).

FACS analysis for cell pyroptosis

For pyroptosis analysis, MHCC97H cells was treated with gradient concentrations of CBD (40 μM) for in a time course at 0, 12, 18, 24hr, cells were then collected and incubated with Annexin V-PE/ 7AAD in the dark at room temperature for 20 min, according to the manufacturer's protocol. Thereafter, cell samples were analyzed immediately using a BD AccuriTM C6 flow cytometer (BD, Franklin Lakes, NJ).

For pyroptosis analysis, Control and GADME-knockdown MHCC97H cells was treated with gradient concentrations of CBD (40 μM) for 24hr, cells were then collected and incubated with Annexin V-PE/ 7AAD in the dark at room temperature for 20 min, according to the manufacturer's protocol. Thereafter, cell samples were analyzed immediately using a BD AccuriTM C6 flow cytometer (BD, Franklin Lakes, NJ).

RNA-sequence analyze

RNA-seq data was generated by Novogene. The CBD exposed HepG2 and MHCC97H cells were collected after 24hr and then cells were washed by PBS for 3 times and then lysed by Trizol at 4℃ for 10 minutes . Next, samples were sent to Novogene on dry ice. A total amount of 3 μg RNA per sample was used as input material for the RNA sample preparations. Sequencing libraries were generated using NEBNext UltraTM RNA Library Prep it for Illumina (NEB, USA) following manufacturer's recommendations, index codes were added to attribute sequences to each sample. The clustering of the index-coded samples was performed on a cBot Cluster Generation System using TruSeq PE Cluster Kit v3-cBot-HS (Illumia) according to the manufacturer's instructions. After cluster generation, the library preparations were sequenced on an Illumina platform and 125 bp/150 bp paired-end reads were generated. In the low-input protocol, RNA was purified using the RNeasy micro kit (Qiagen) to obtain 1–10 ng per pool. First-strand cDNA synthesis and amplification were performed using SMARTer Ultra-Low-Input RNA amplification kit v3 (Clontech). Single-end libraries were prepared using NEBNext Ultra DNA library prep for Illumina and sequenced on a NextSeq 500 instrument, yielding ~11 million reads (range 8–13 million) of 75 bp in length. Low-quality ends and adaptor sequences were trimmed from the Illumina reads with FastX 0.0.13 and cutadapt 1.7.1. Using FastX and ShortRead 1.16.3, we subsequently filtered out short reads (length <35 bp), poly(A) reads (where >90% of the bases are adenine), ambiguous reads (containing Ns) and low-quality reads (where >50% of the bases have quality <Q25). Heatmaps were generated using the R package pheatmap (version 1.0.8). The input values were log transformed FPKM or fold-change. Heatmaps were centered and scaled in the row direction. Euclidean distance

and complete method were used for Hierarchical Clustering.

XF Extracellular Flux Analyzer Experiments

The intact cellular oxygen consumption rate (OCR) and the extracellular acidification rate (ECAR) in compound treated HepG2, HUH7, and MHCC97H cells were measured using a Seahorse XF-96 Extracellular Flux Analyzer (Seahorse Bioscience, North Billerica, MA) as described previously. Briefly, 80 μL Single-cell suspensions of HepG2, HUH7, and MHCC97H cells were plated in XF96 cell culture microplates (Seahorse Bioscience) at a cellular density of 25,000, 20,000 and 20,000 cells respectively. The next day, the HepG2, HUH7, and MHCC97H cells were treated with gradient concentrations of CBD (0, 20, and 40 μM) for 4 hr. For OCR determinations, cells were incubated in base assay medium (according to manufacturer's instructions) supplemented with 2 mM glutamine, 10 mM glucose, and 1 mM pyruvate for 1 hr, prior to the measurements using the XF Cell Mito Stress Kit (Seahorse Bioscience). The final concentrations of Oligomycin, FCCP, and rotenone were 0.1 μM. For glycolytic metabolism measurements, cells were incubated in basal media prior to injections using the Glycolytic Test kit (Seahorse Bioscience). Results were obtained from three independent experiments; each with 8 replicates of each group of cells. At the end of each assay, a BCA protein assay kit was used to determine and normalize the protein concentrations, according to the manufacturer's instructions.

FACS analysis for glucose uptake by 2-NBDG

For glucose uptake analysis, the HepG2 and MHCC97H cells were treated with gradient concentrations of CBD (0, 20, and 40 μM) for 24 hr, cells were then collected and incubated with 2-NBDG according to the manufacturer's instructions in the dark at 37°C for 20 min.

Thereafter, cell samples were analyzed immediately using a BD AccuriTM C6 flow cytometer (BD, Franklin Lakes, NJ).

Statistical analysis

All statistical analyses were performed with the SPSS 16.0 statistical software package (SPSS Standard version 16.0, SPSS Inc., Chicago, IL). Data are shown as the mean ± SD from at least three independent experiments. Groups of 2 were analyzed with two-tailed students t-test, groups greater than 2 with a single variable were compared using one-way ANOVA analysis with Tukey post hoc test, $p < 0.05$ was considered statistically significant.

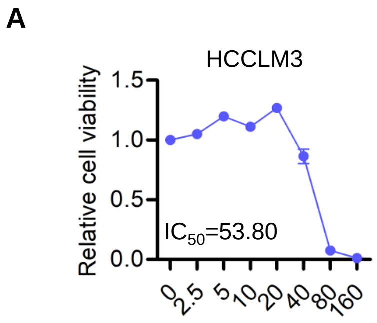

Figure S1. HCCLM3 cells showed lower sensibility than HepG2, HUH7, and MHCC97H cells. A. HCCLM3 cells were treated with a gradient concentration (0, 2.5, 5, 10, 20, 40, 80, or 160 μM) of CBD for 24 hr. Relative cell viability was analyzed by CCK-8 assay.

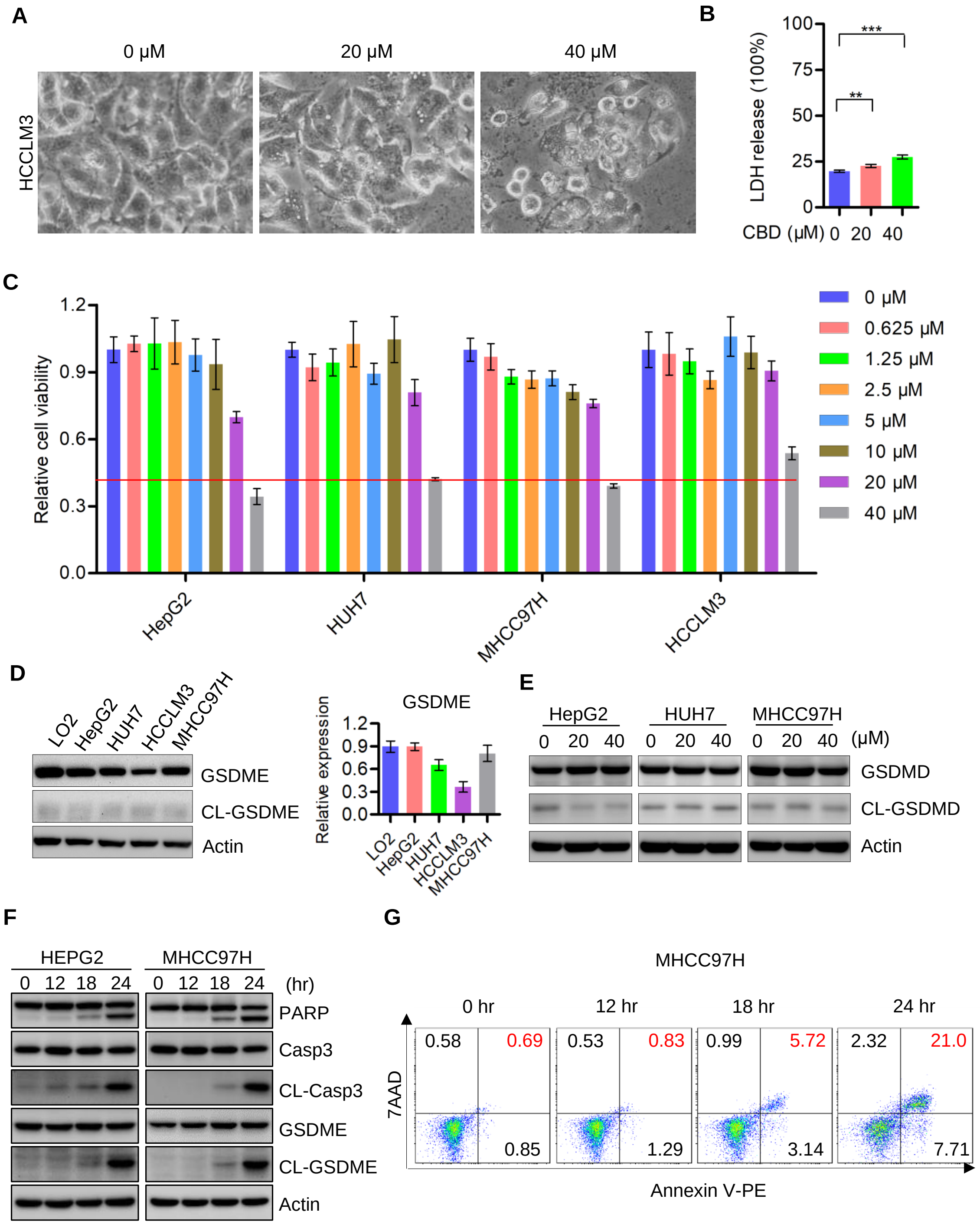

Figure S2. GSDME protein expression has a positive correlation with the cell sensitivity to CBD exposure in HCC cells. A. Morphological changes of HCCLM3 cells in response to different concentrations of CBD. **B.** LDH release assays were performed on CBD exposed HCCLM3 cells for 24hr. **C.** HepG2, HUH7, HCCLM3, and MHCC97H cells were treated with a

gradient concentration (0, 0.625, 1.25, 2.5, 5, 10, 20, or 40 μM) of CBD for 48 hr. Relative cell viability was analyzed by CCK-8 assay. **D.** Western blot analysis of GSDME in LO2, HepG2, HUH7, HCCLM3, and MHCC97H cells, and Actin was used as a loading control. The graphs represent the mean ± SD. **E.** Western blot analysis of GSDMD and CL-GSDMD in CBD exposed HepG2, HUH7, and MHCC97H cells, and Actin was used as a loading control. **F.** HEPG2 and MHCC97H cells were treated in a time course (0 12 18 24 hr) with CBD (40 μM),and then cells were collected and anlysied by Western blotting, and Actin was used as a loading control. **G.** Flow cytometry analysis of cell apoptosis after the MHCC97H cells treated with a time course (0 12 18 24 hr) of CBD. Cells were collected and stained with Annexin V-PE/7AAD.

A

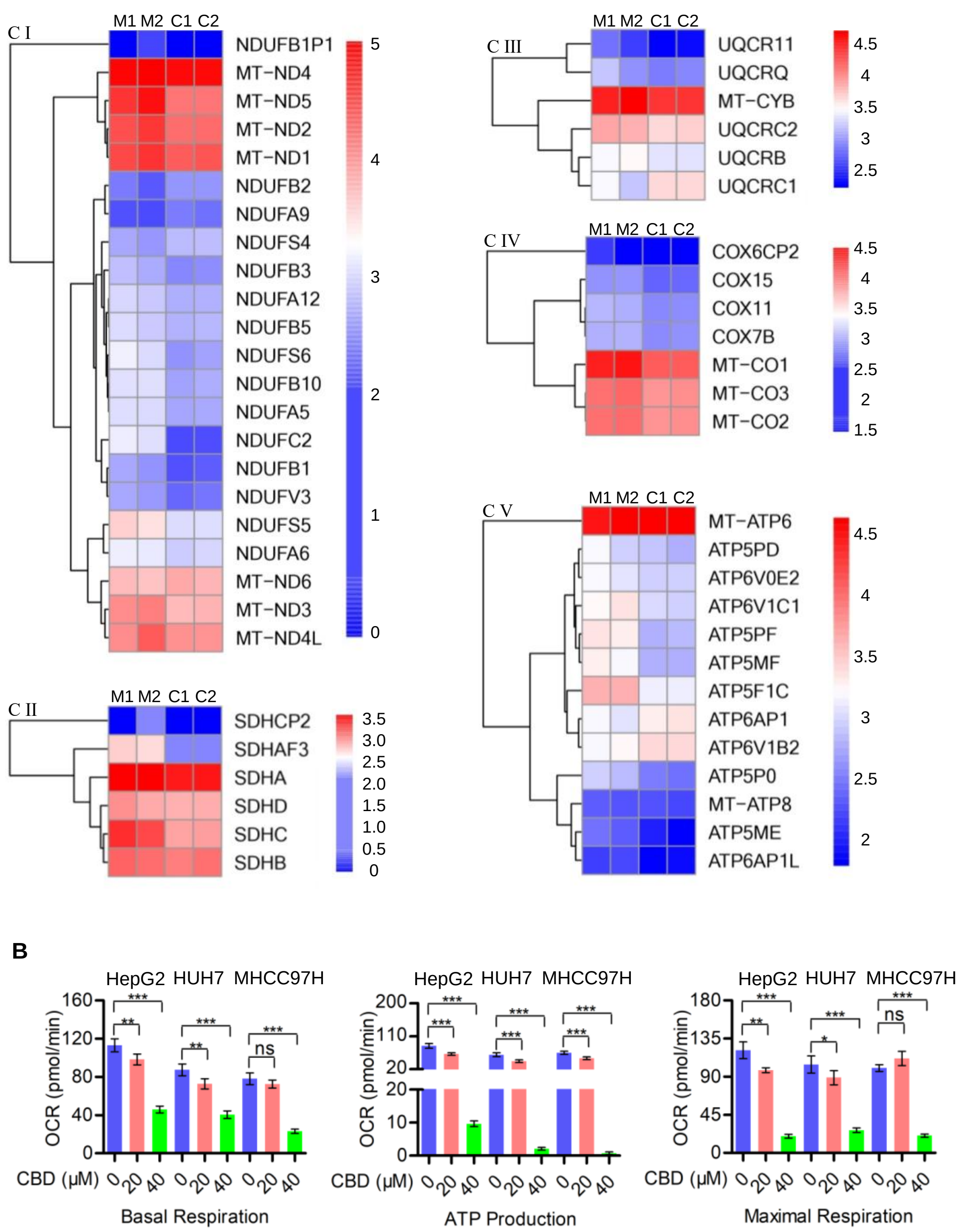

Figure S3. Mitochondria homeostasis was impaired by CBD exposing in HCC cells. A. Heat map analysis of the changes in mitochondrial respiratory chain proteins. **B.** OCR of CBD treated HepG2, HUH7, and MHCC97H cells in the indicated conditions were measured in real time using the Seahorse XF96 Extracellular Flux Analyzer. Data showed a significant reducing of OCR indexes as basal respiration, ATP production, and maximal respiration.

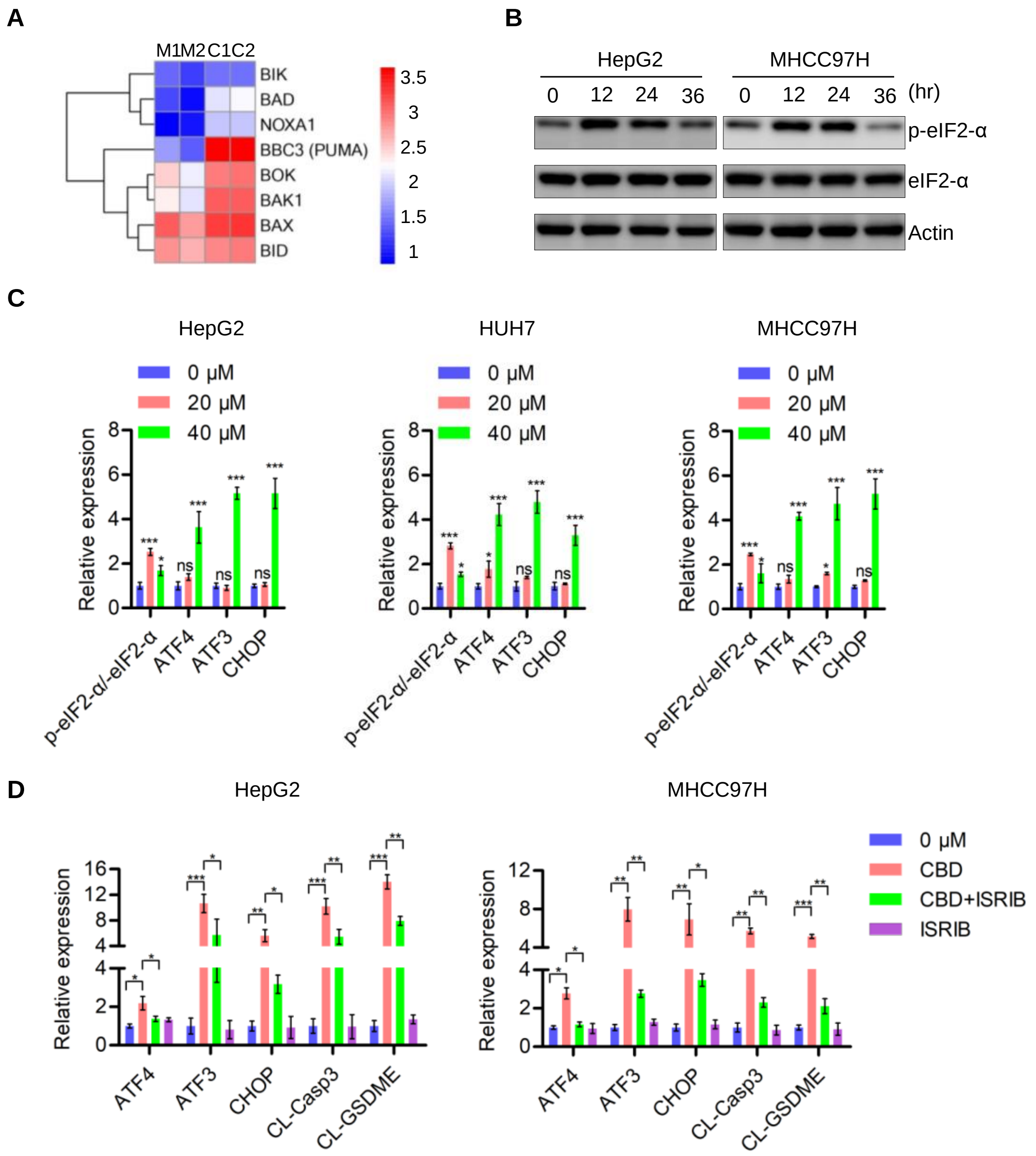

Figure S4. Activation of ATF4/CHOP pathway in ISR-dependent way triggers the GSDME-mediated pyroptosis in response to CBD treatment. A. Heat map analysis of the changes in pro-apoptotic proteins of BCL-2 family. **B**. Western blot analysis of p-eIF2-α and eIF2-α in CBD-treated HepG2, and MHCC97H cells in 12, 24, and 36 hours. Actin was used as a loading control. **C.** Protein quantitative analysis of ATF4, ATF3, and CHOP expression in CBD-treated HepG2, HUH7, and MHCC97H cells. **D.** Protein quantitative analysis of ATF4, ATF3, CHOP, CL-caspase-3, and CL-GSDME expression in CBD exposed HepG2 and MHCC97H cells with or without ISRIB.

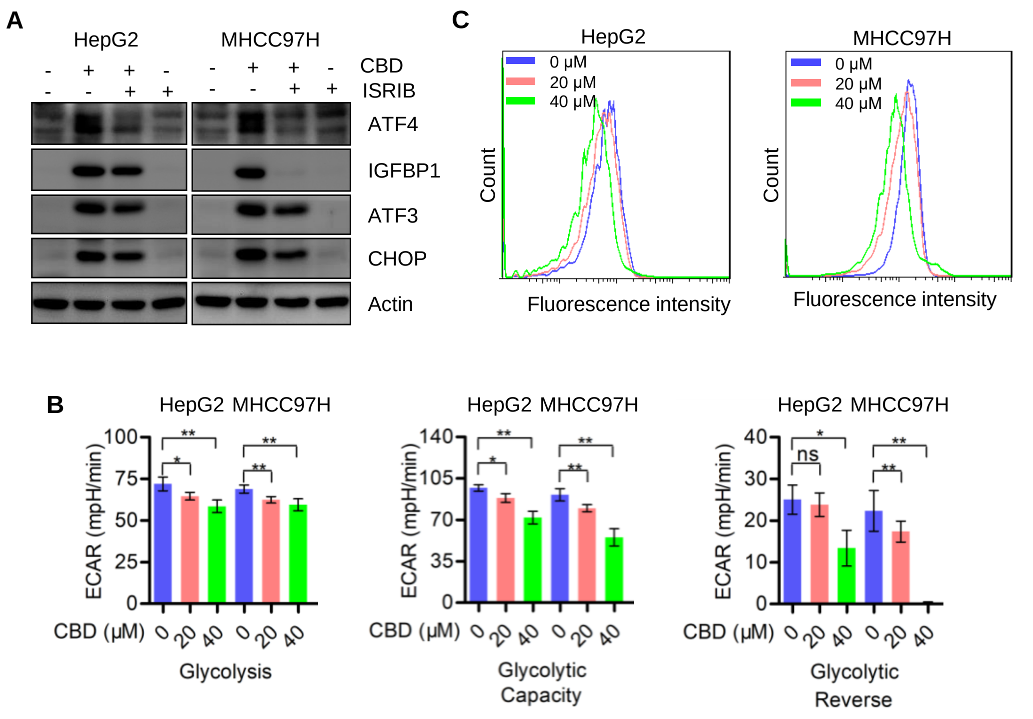

Figure S5. CBD triggers ATF4/IGFBP1 axis activation to depress cell glycolysis in HCC cells. A. Western blot analysis of ATF4, IGFBP1, ATF3, and CHOP in CBD-treated HepG2, and MHCC97H cells in the presence or absence of ISRIB, and Actin was used as a loading control. **B.** Extracellular acidification rate (ECAR) of CBD treated HepG2 and MHCC97H cells in the indicated conditions were measured in real time using the Seahorse XF96 Extracellular Flux Analyzer. Data showed that basal glycolysis, glycolytic capacity, and glycolytic reverse were increased in the CBD-treated HepG2 and MHCC97H cells remarkably. **C.** 2-NBDG was used to determine glucose absorption in CBD-treated HepG2 and MHCC97H cells

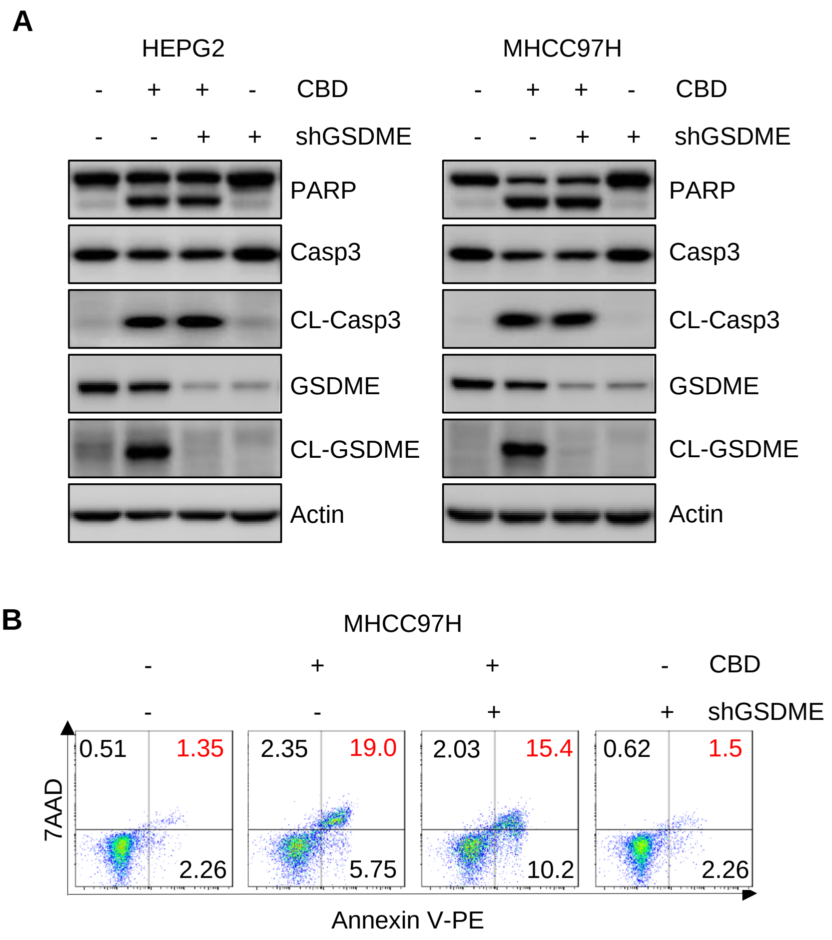

A. Control and GSDME-depletion HEPG2 and MHCC97H cells were treated with CBD (40 μM) for 24 hr,and then cells were collected and anlysied by Western blotting, and Actin was used as a loading control. **B.** Flow cytometry analysis of cell apoptosis after the Control and GSDME-depletion MHCC97H cells treated with CBD for 24 hr. Cells were collected and stained with Annexin V-PE/7AAD.
