# Supplementary material for: A Novel Mechanism of Cannabidiol in Suppressing Hepatocellular Carcinoma by Inducing GSDME Dependent Pyroptosis
Source: Front Cell Dev Biol. 2021 Jul 19;9:697832. doi: 10.3389/fcell.2021.697832 (PMC8327166; doi:10.3389/fcell.2021.697832)
Supplement: Supplementary file 3 [file Data_Sheet_1.zip › PDF-WB-RAW-DATA/WB-Figure 2D.pdf]

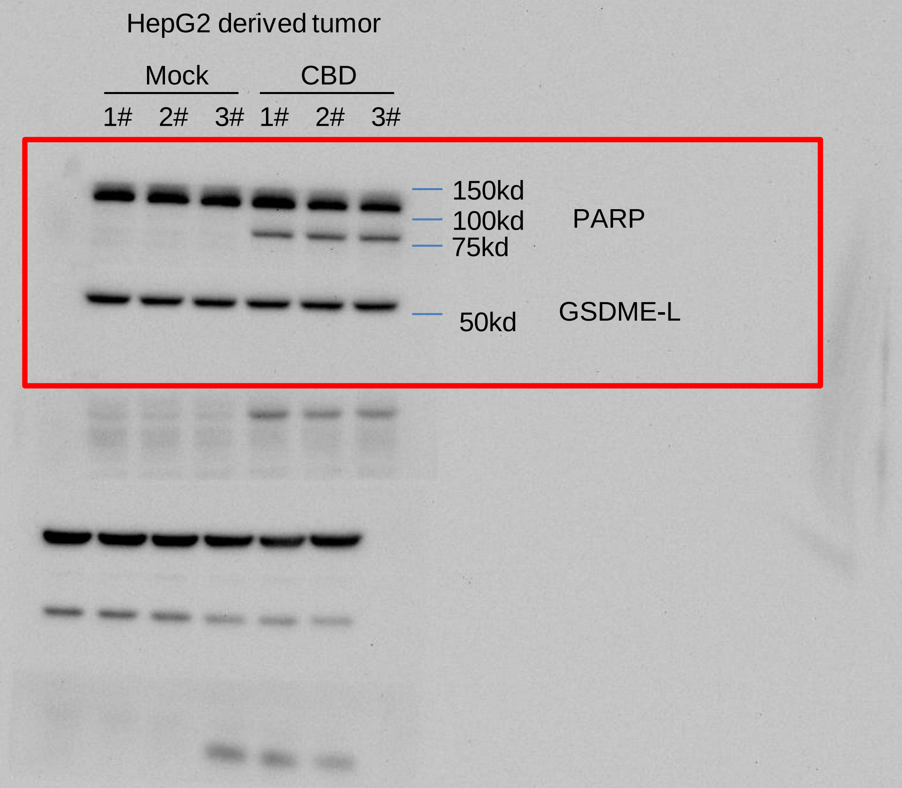
HepG2 derived tumor
Mock
CBD
1# 2# 3# 1# 2# 3#
150kd
100kd
75kd
PARP
50kd
GSDME-L

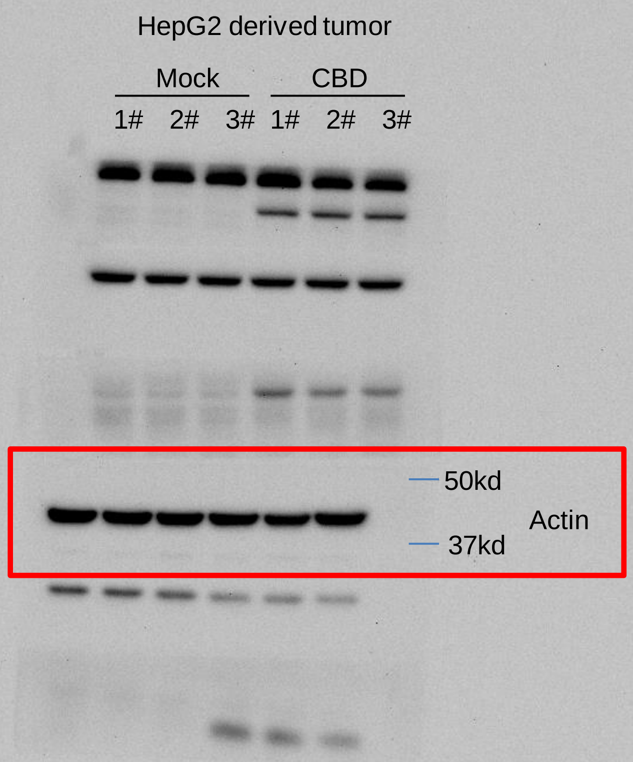
HepG2 derived tumor
Mock
CBD
1#
2#
3#
1#
2#
3#
50kd
Actin
37kd

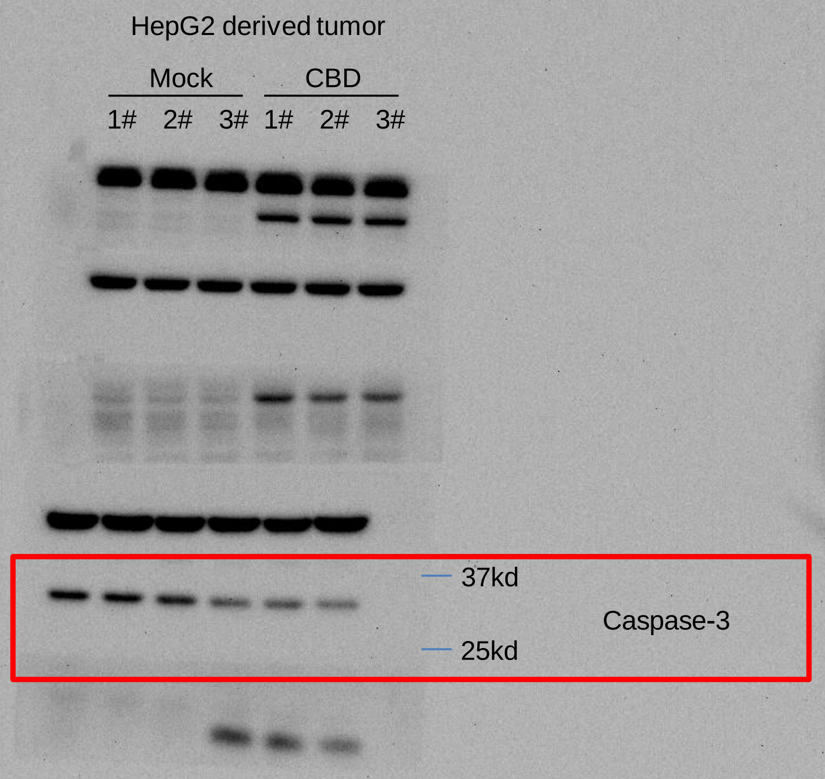
HepG2 derived tumor
Mock
CBD
1#
2#
3#
1#
2#
3#
37kd
Caspase-3
25kd

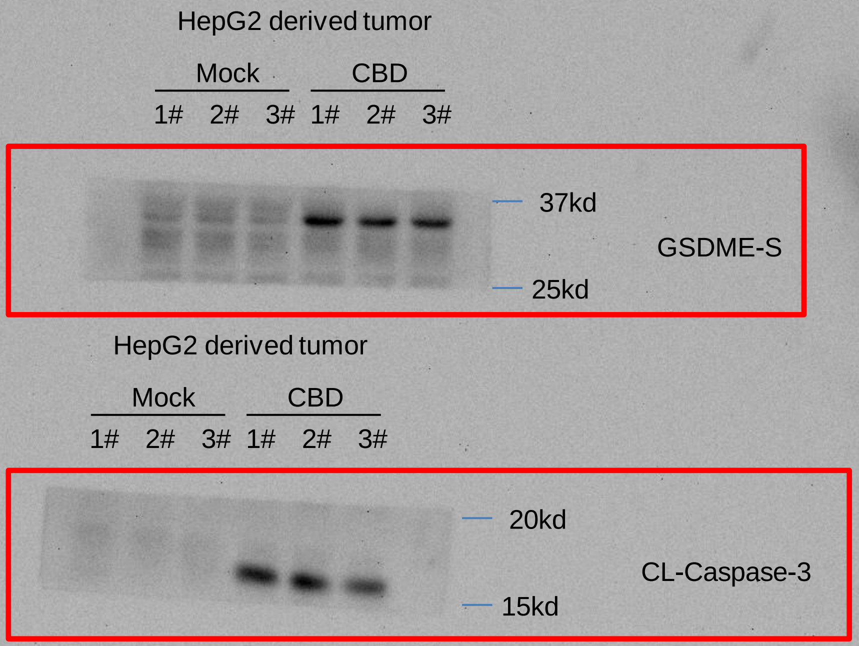
HepG2 derived tumor
Mock
CBD
1# 2# 3# 1# 2# 3#
37kd
GSDME-S
25kd
HepG2 derived tumor
Mock
CBD
1# 2# 3# 1# 2# 3#
20kd
CL-Caspase-3
15kd
