# Supplementary material for: A Novel Mechanism of Cannabidiol in Suppressing Hepatocellular Carcinoma by Inducing GSDME Dependent Pyroptosis
Source: Front Cell Dev Biol. 2021 Jul 19;9:697832. doi: 10.3389/fcell.2021.697832 (PMC8327166; doi:10.3389/fcell.2021.697832)
Supplement: Supplementary file 3 [file Data_Sheet_1.zip › PDF-WB-RAW-DATA/WB-Figure 3F-MHCC97H.pdf]

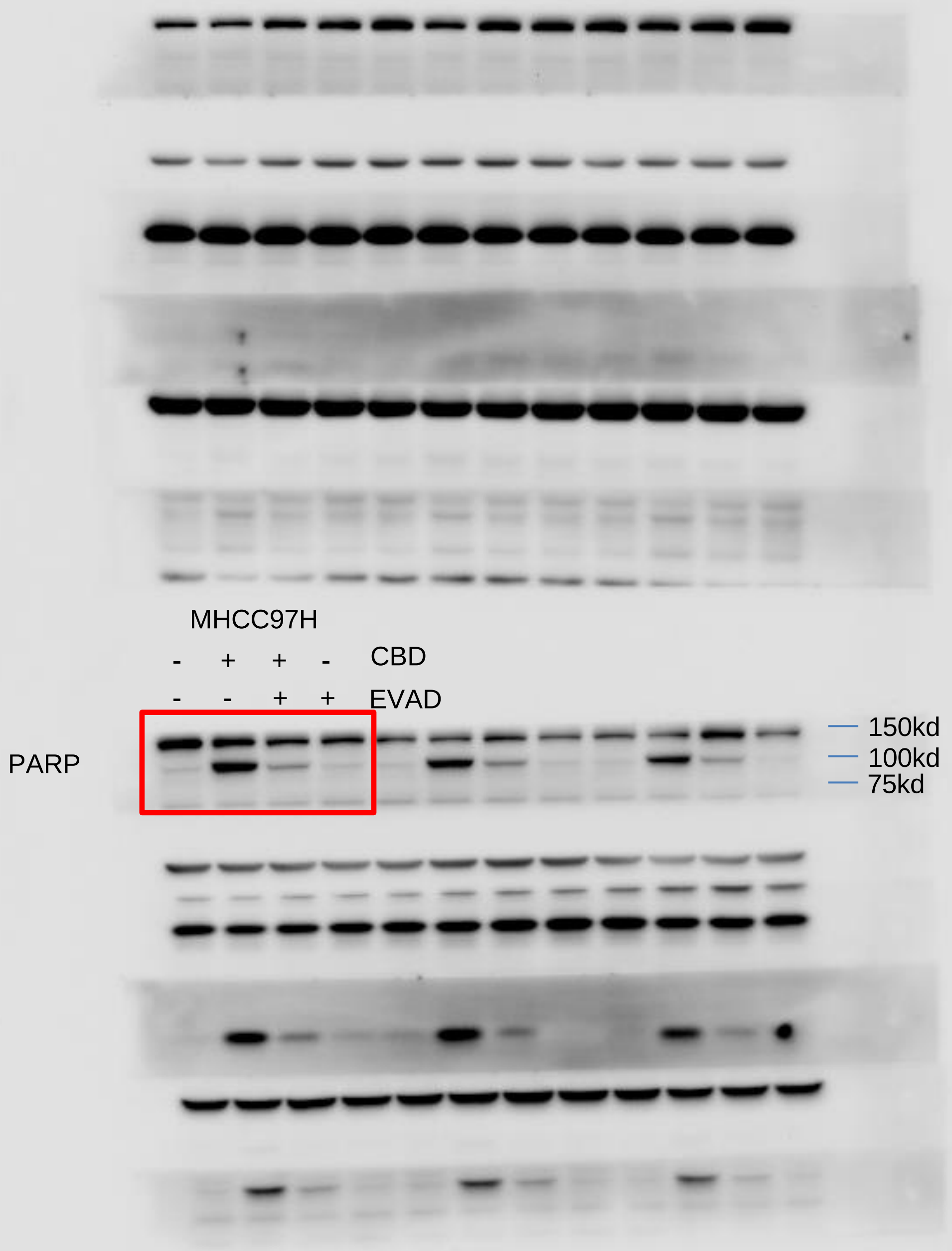
MHCC97H
- + + - CBD
- - + + EVAD
PARP
150kd
100kd
75kd

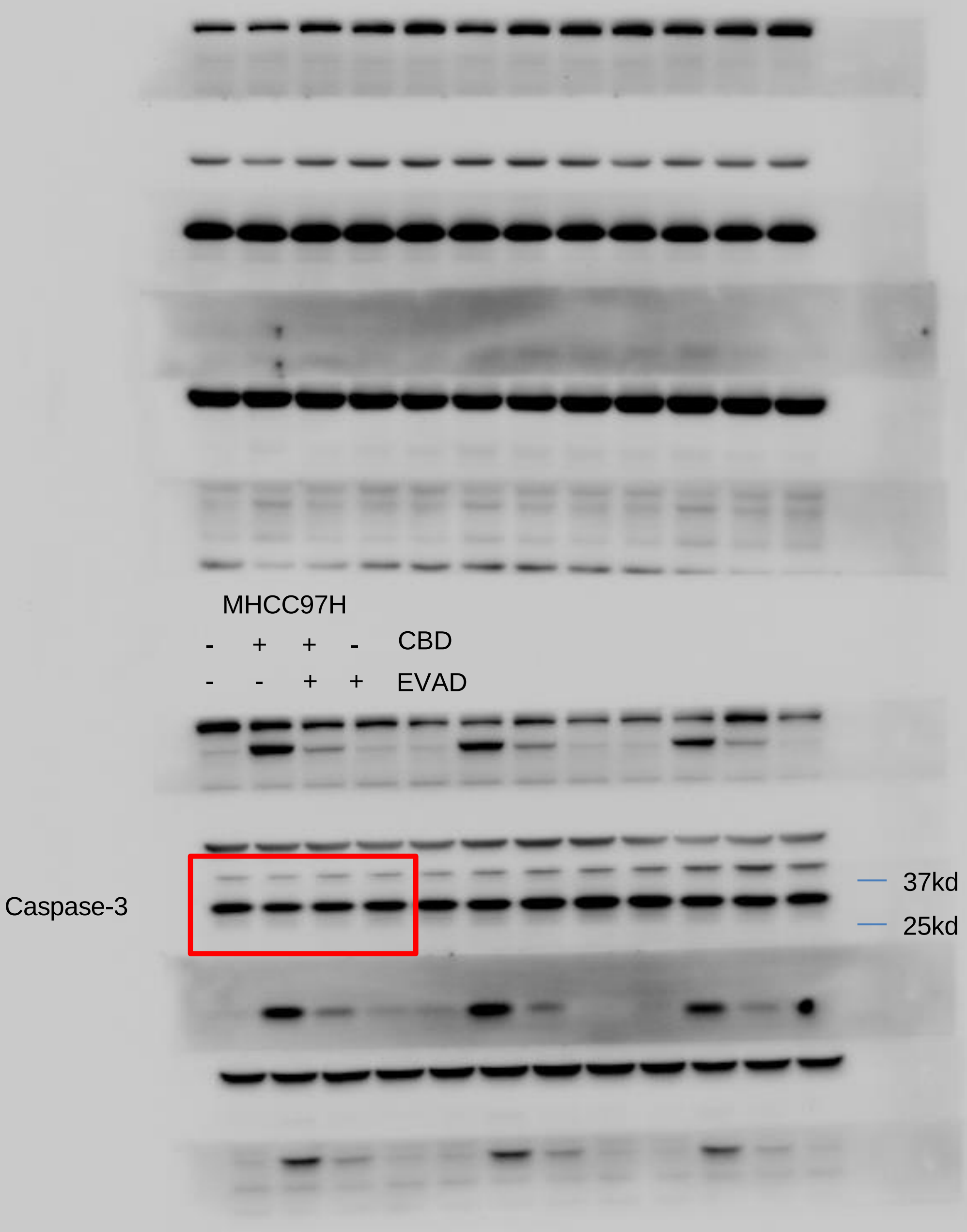
MHCC97H
- + + - CBD
- - + + EVAD
Caspase-3
37kd
25kd

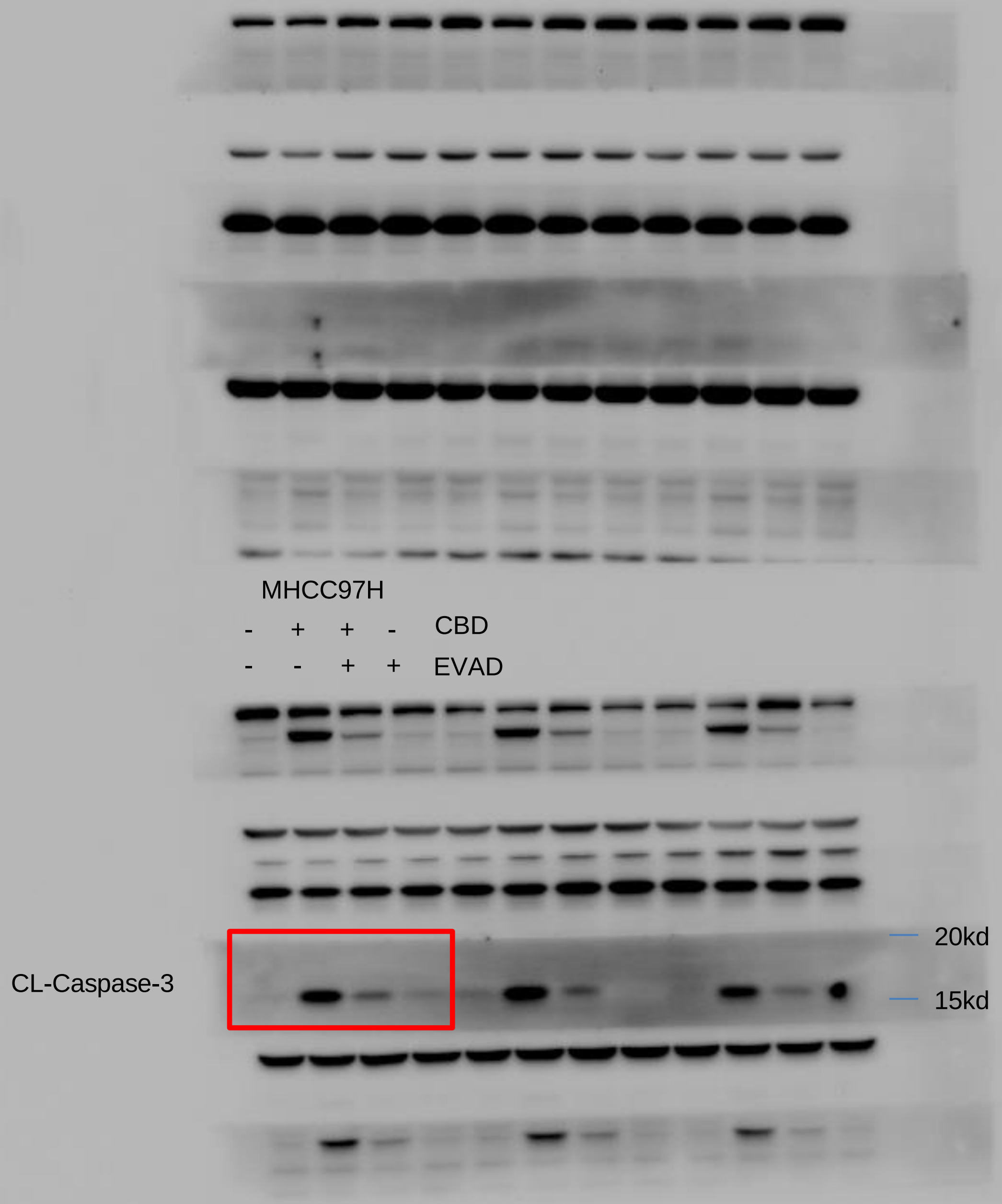
MHCC97H
- + + - CBD
- - + + EVAD
CL-Caspase-3
20kd
15kd

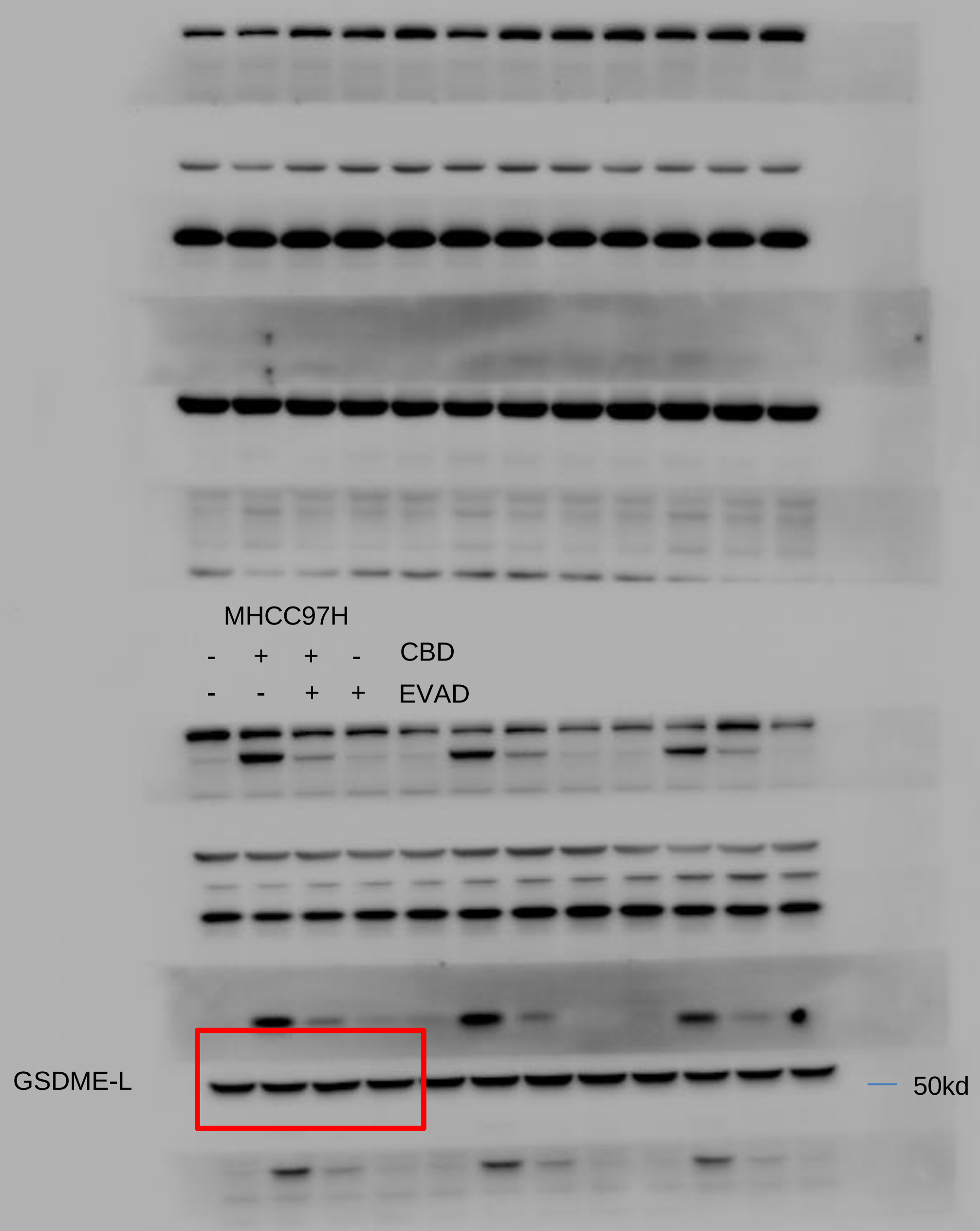
MHCC97H
- + + - CBD
- - + + EVAD
GSDME-L
50kd

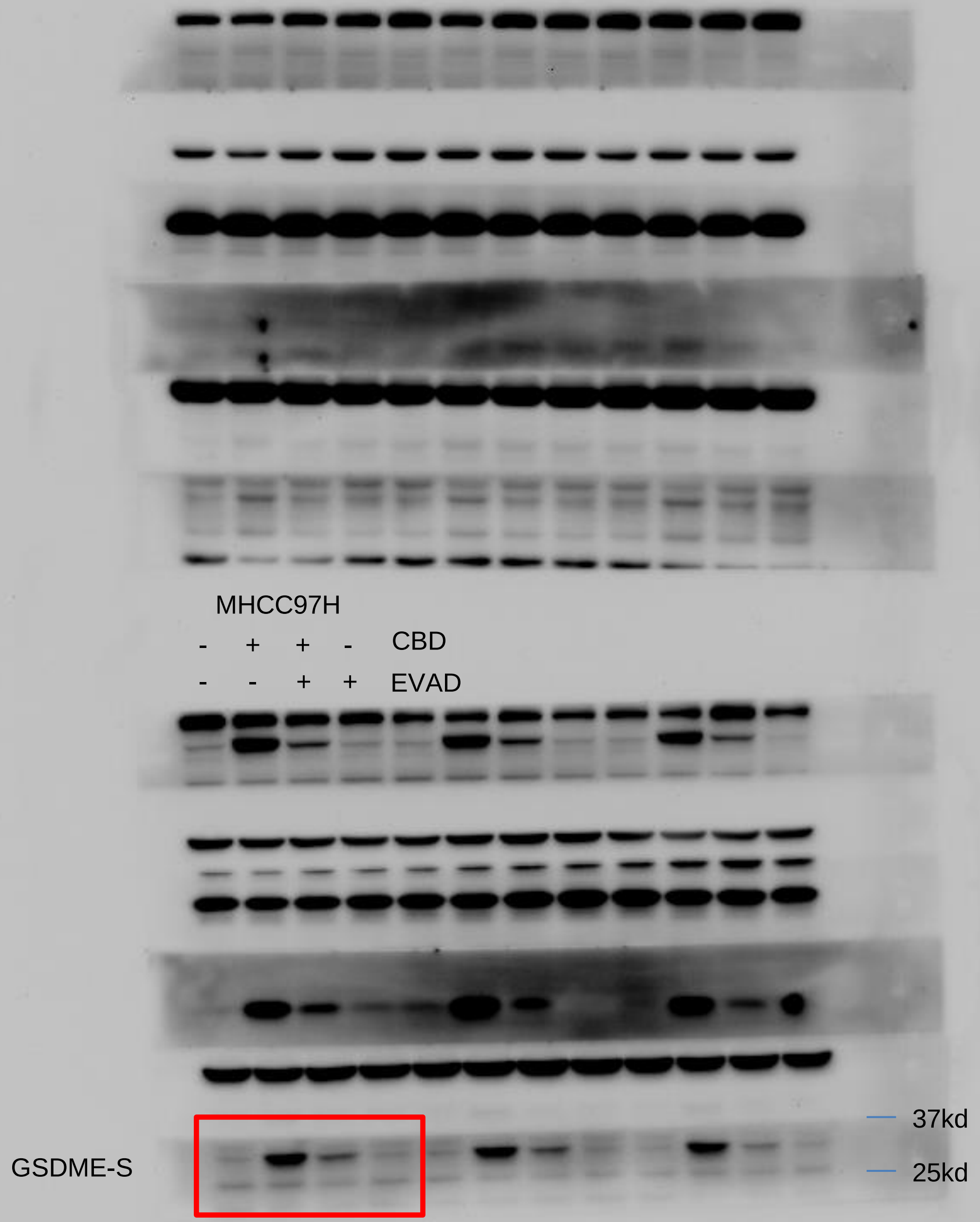
MHCC97H
- + + - CBD
- - + + EVAD
GSDME-S
37kd
25kd

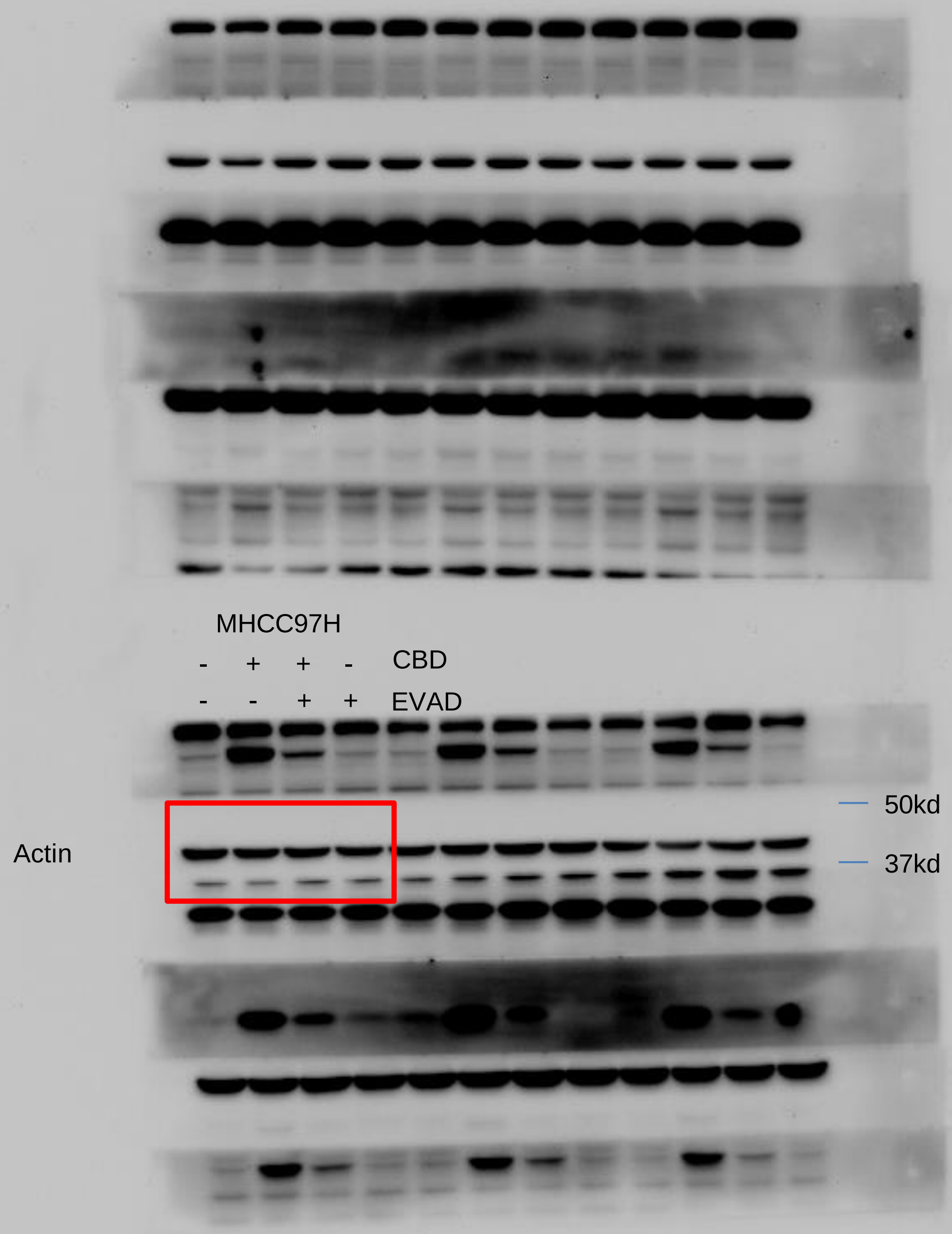

MHCC97H
- + + - CBD
- - + + EVAD
Actin
50kd
37kd
