# Supplementary figures and images for: A Novel Mechanism of Cannabidiol in Suppressing Hepatocellular Carcinoma by Inducing GSDME Dependent Pyroptosis
Source: Front Cell Dev Biol. 2021 Jul 19;9:697832. doi: 10.3389/fcell.2021.697832 (PMC8327166; doi:10.3389/fcell.2021.697832)

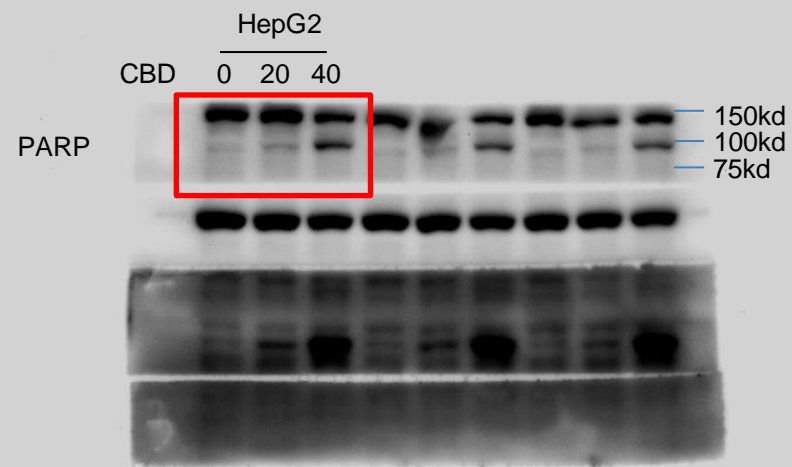

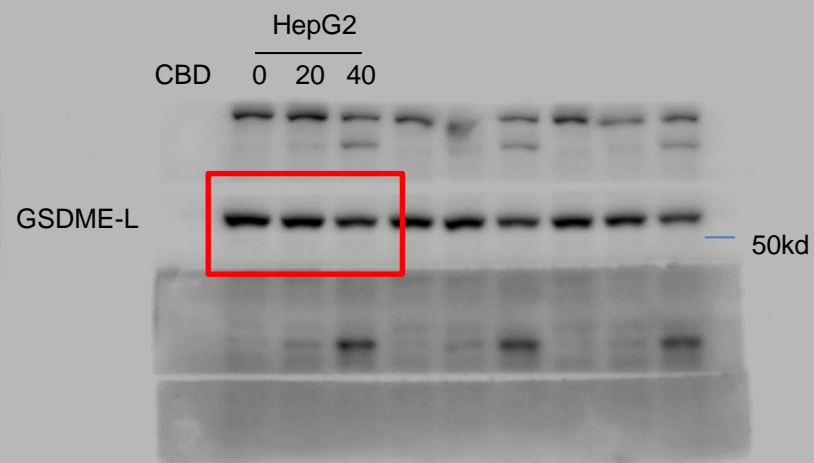

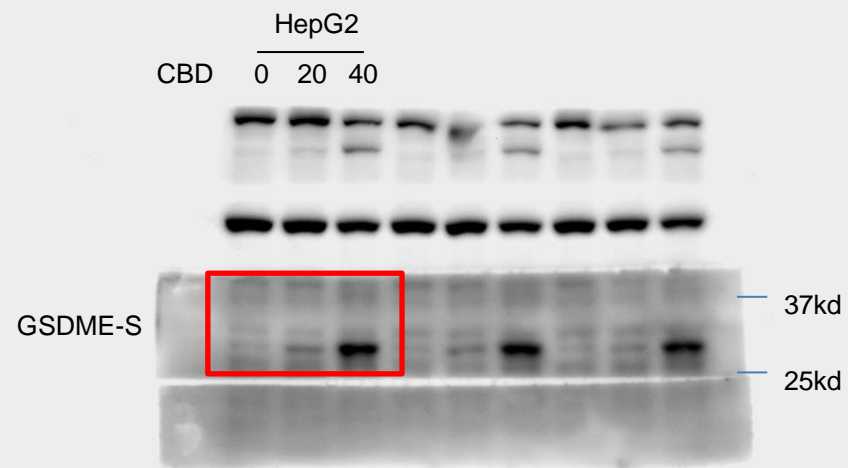

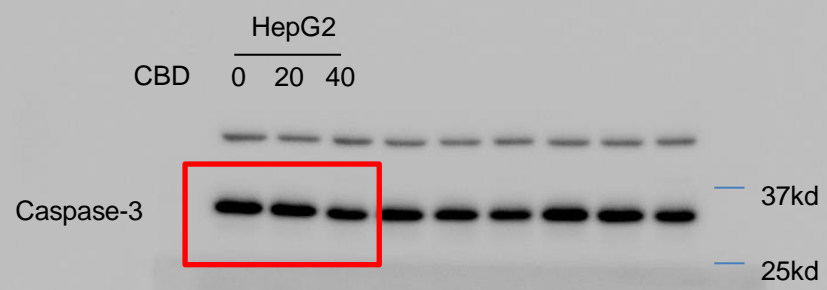

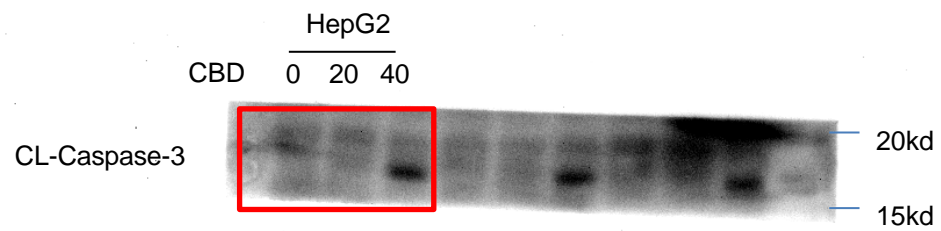

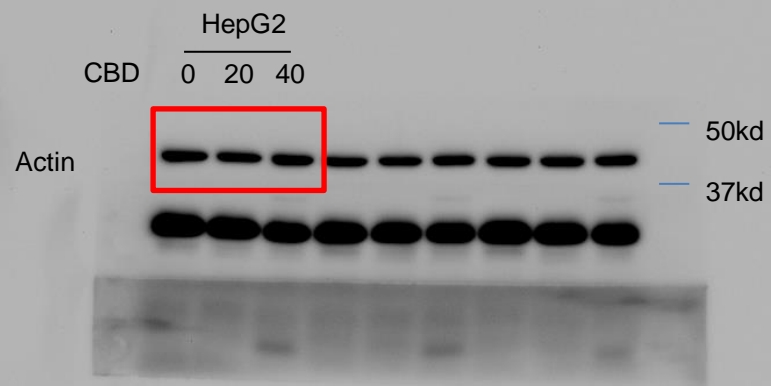

Supplement: Supplementary file 3 [file Data_Sheet_1.zip › PDF-WB-RAW-DATA/WB-Figure 2E-HepG2.pdf]

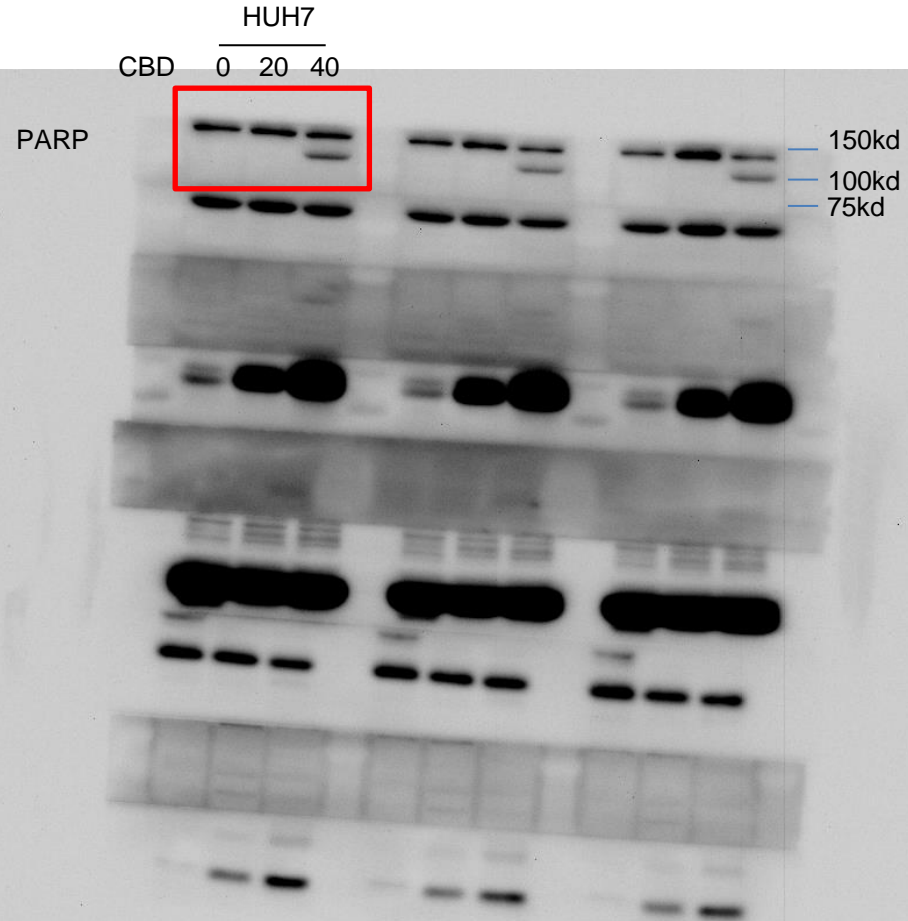

CBD      HUH7  
            0   20 40

GSDME-L

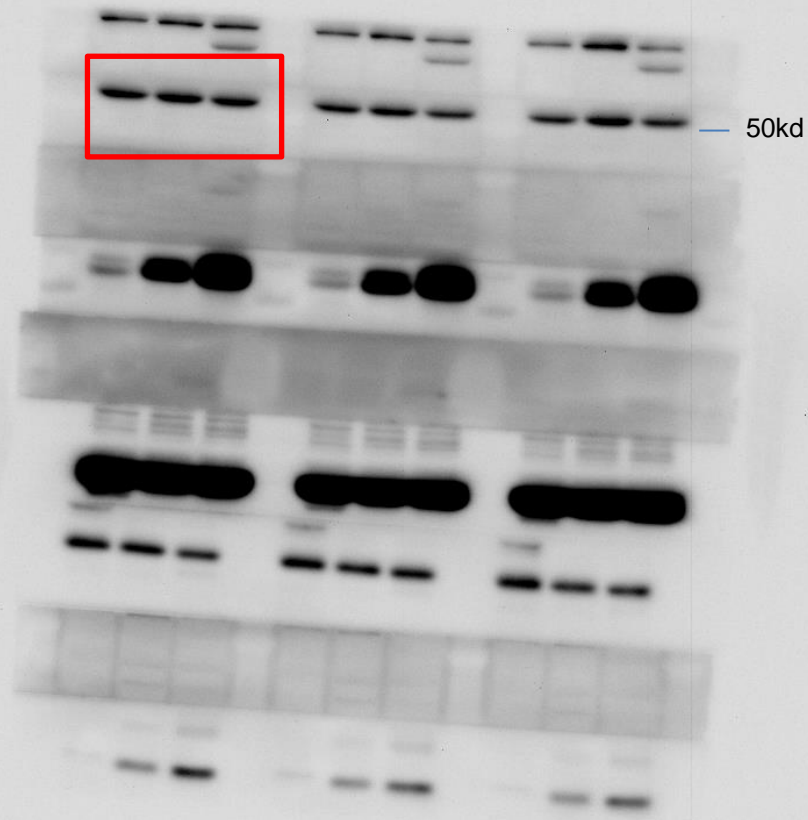

CBD      HUH7  
            0   20 40

GSDME-S

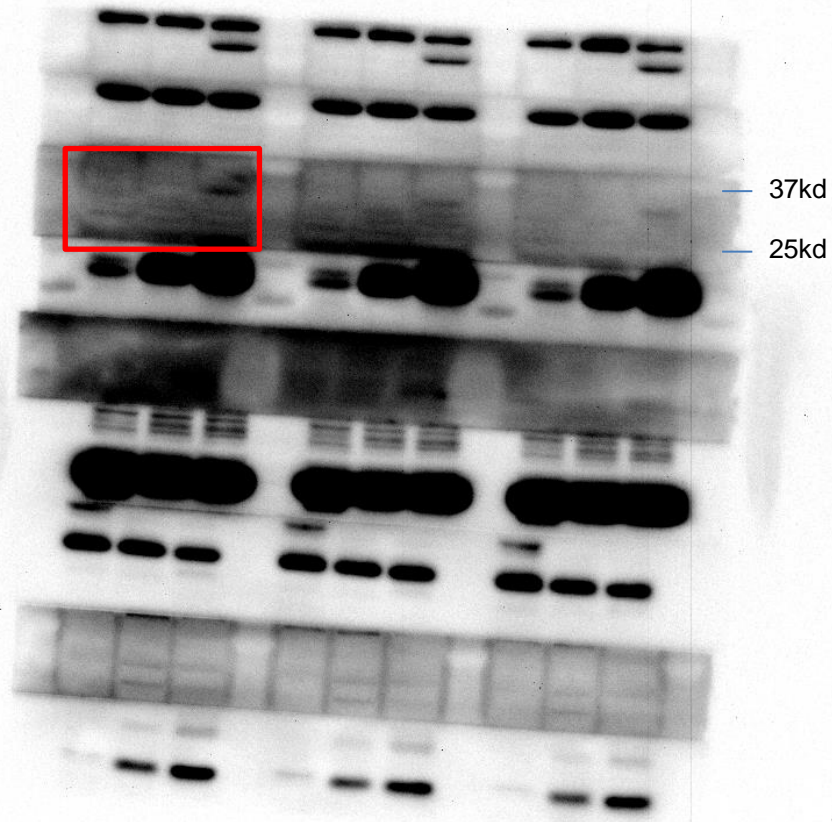

CBD      HUH7  
            0   20 40

Caspase-3

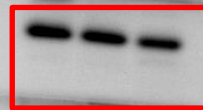

— 37kd

— 25kd

CBD           HUH7  
          0    20 40

CL-Caspase-3

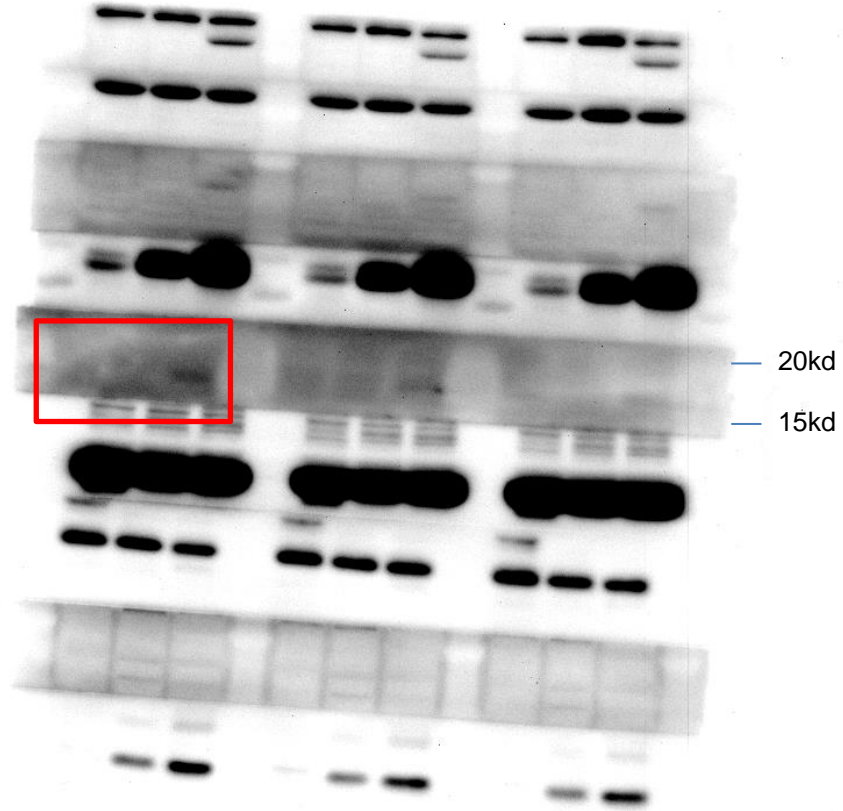

CBD        H7    
          0   20 40

Actin

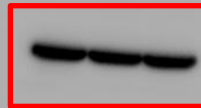

— 50kd

— 37kd

Supplement: Supplementary file 3 [file Data_Sheet_1.zip › PDF-WB-RAW-DATA/WB-Figure 2E-HUH7.pdf]

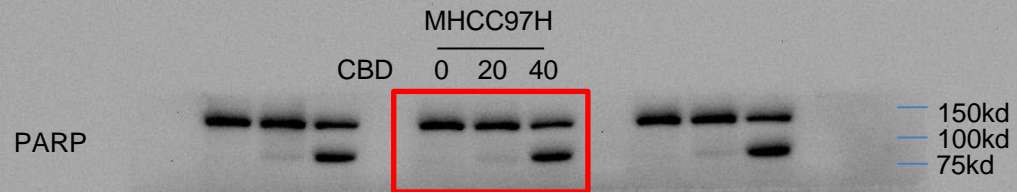

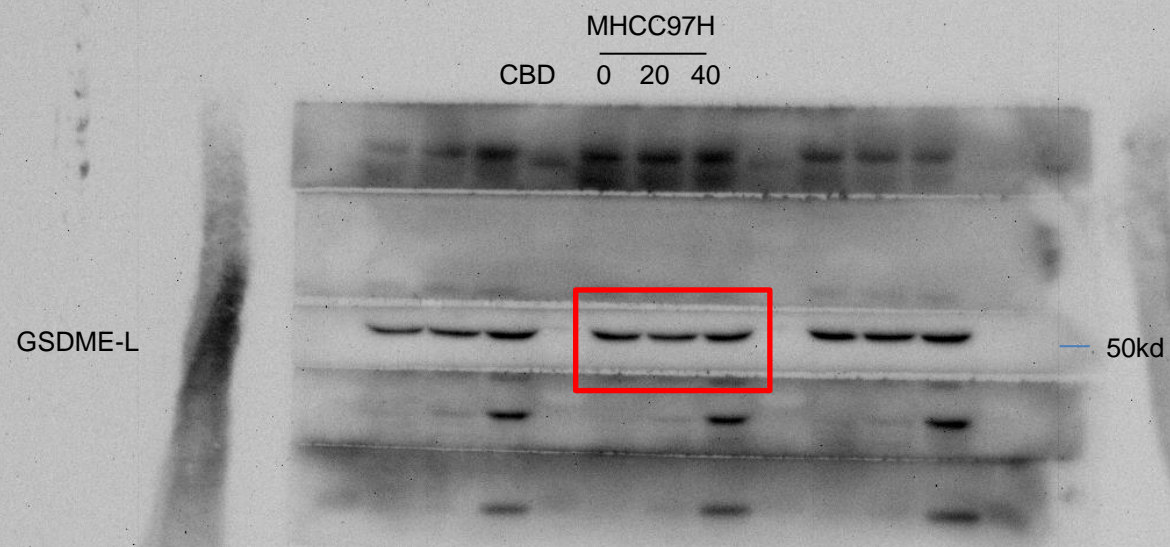

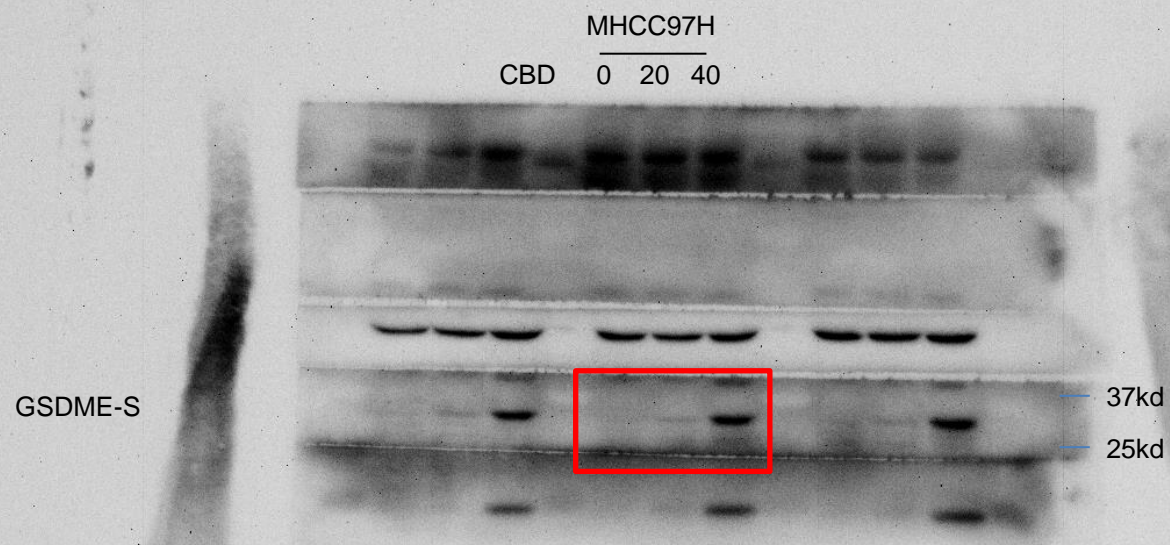

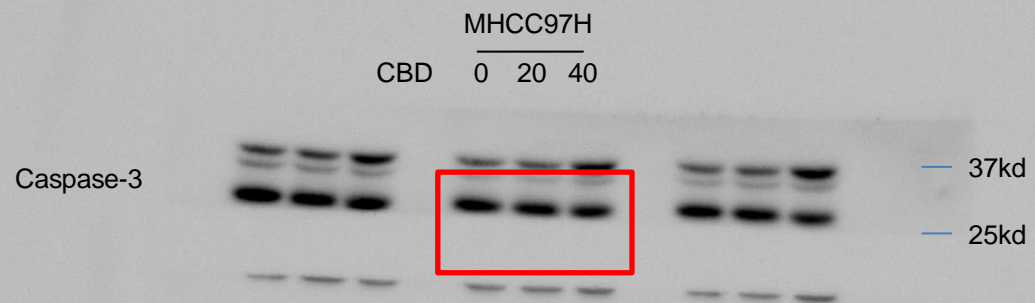

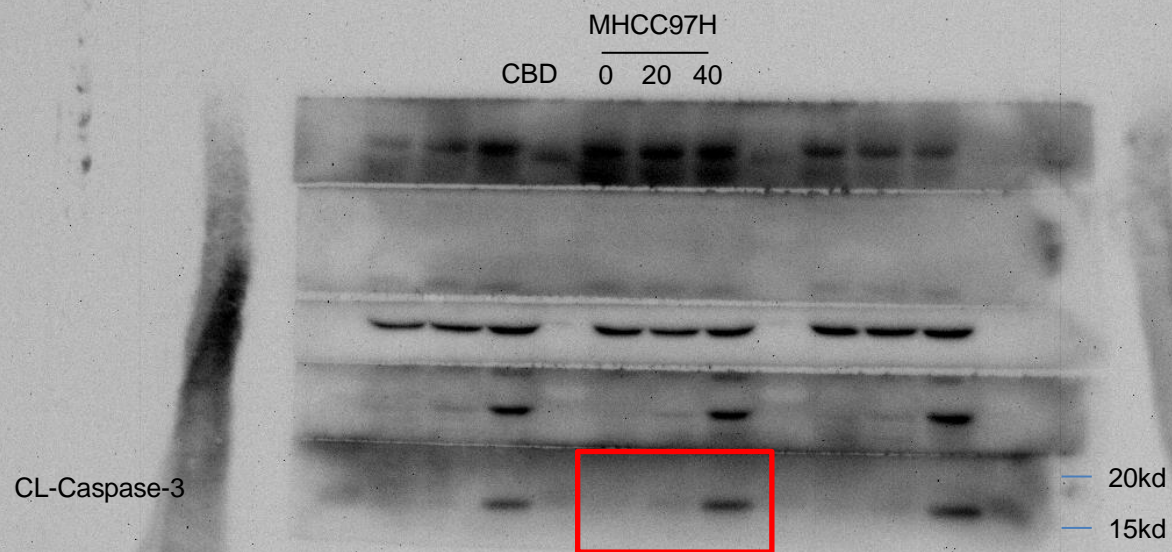

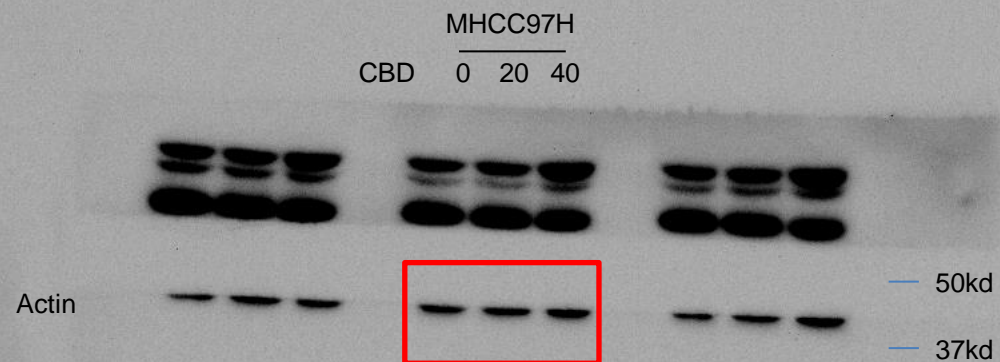

Supplement: Supplementary file 3 [file Data_Sheet_1.zip › PDF-WB-RAW-DATA/WB-Figure 2E-MHCC97H.pdf]

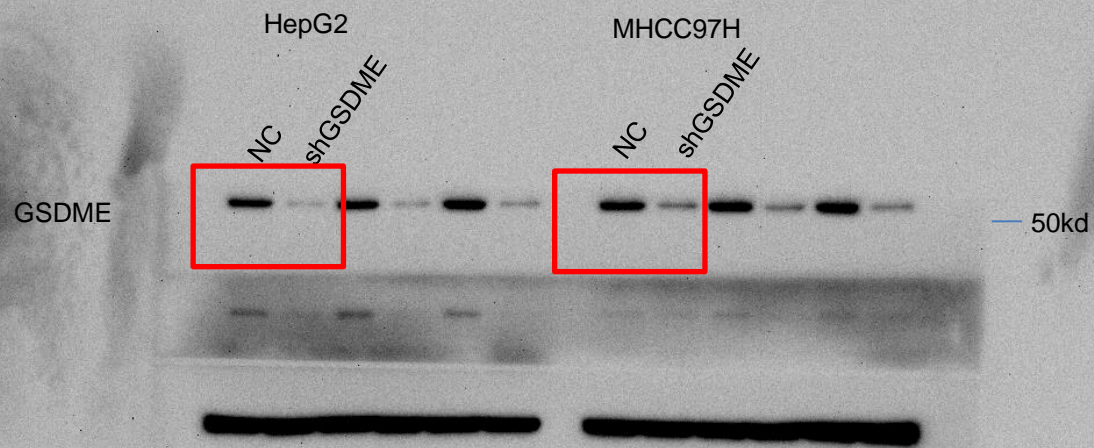

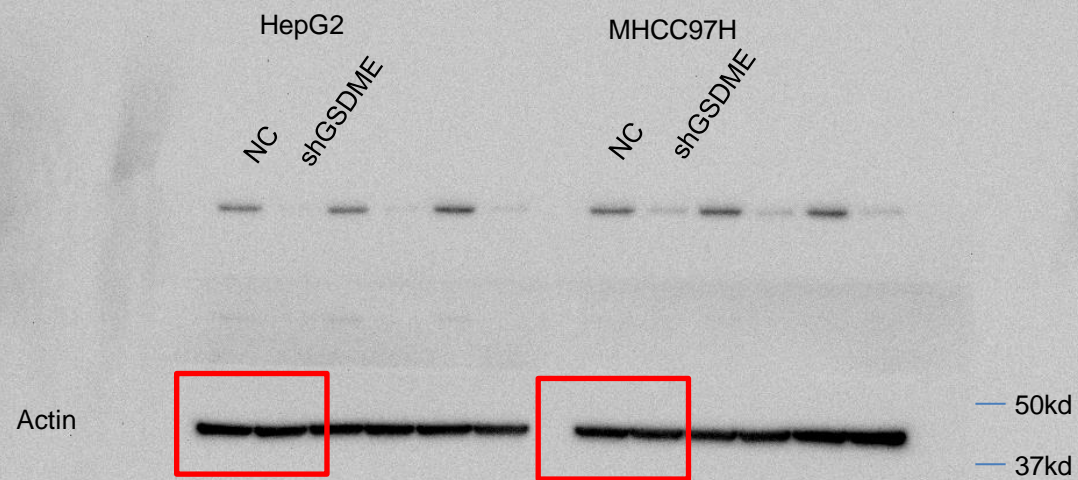

Supplement: Supplementary file 3 [file Data_Sheet_1.zip › PDF-WB-RAW-DATA/WB-Figure 3A.pdf]

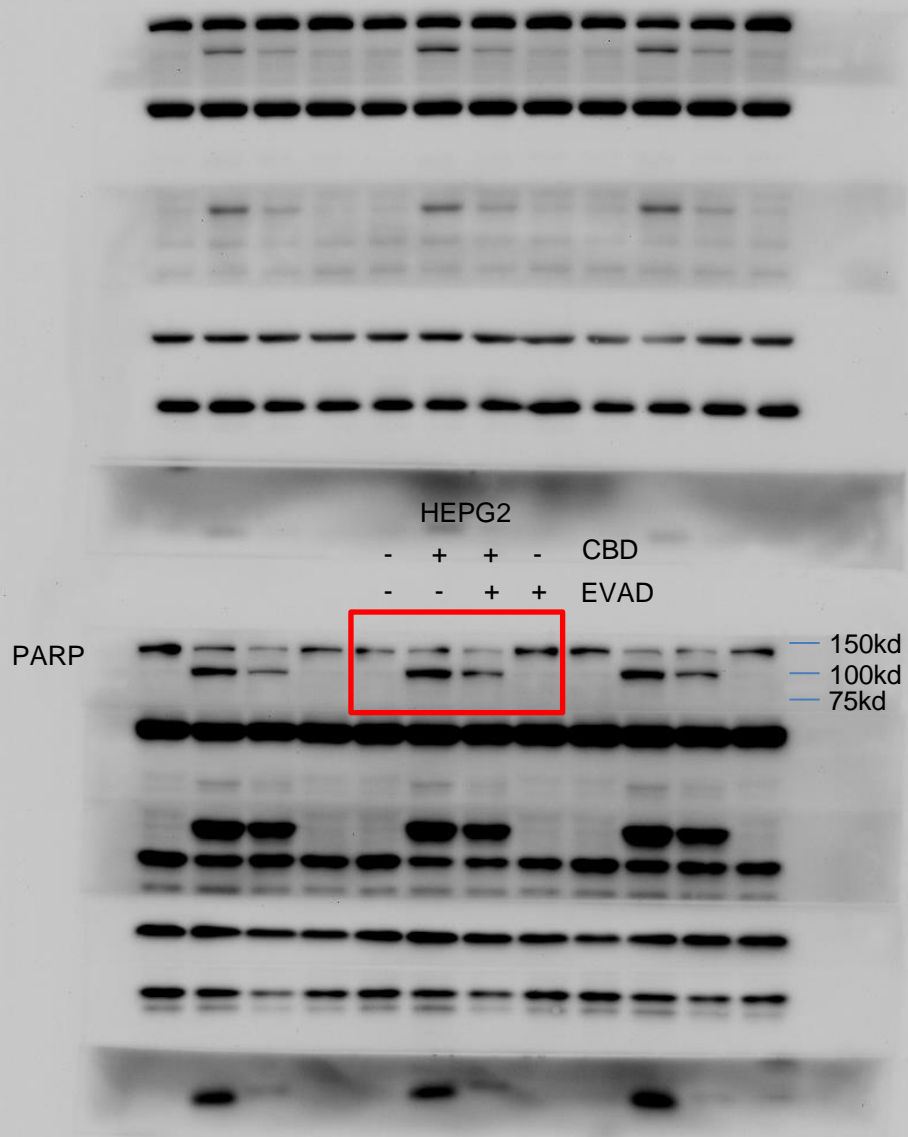

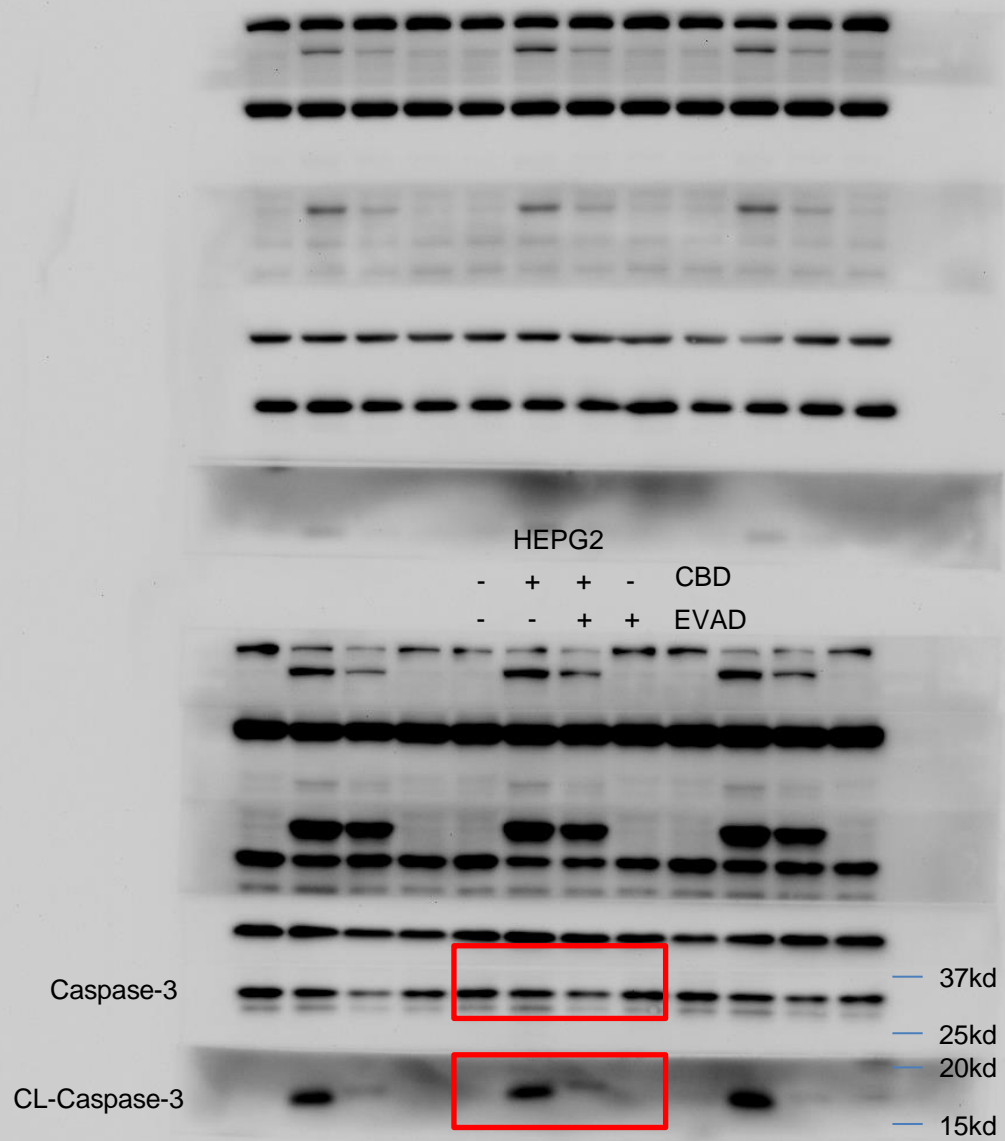

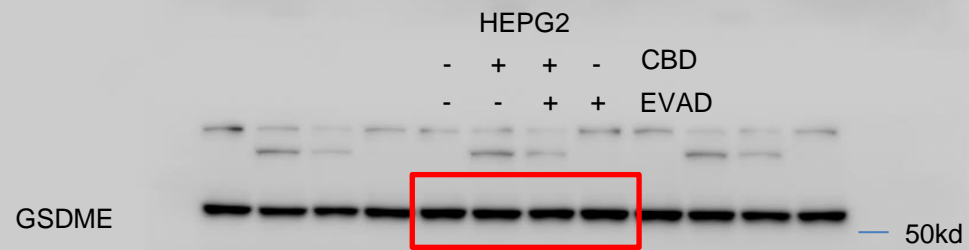

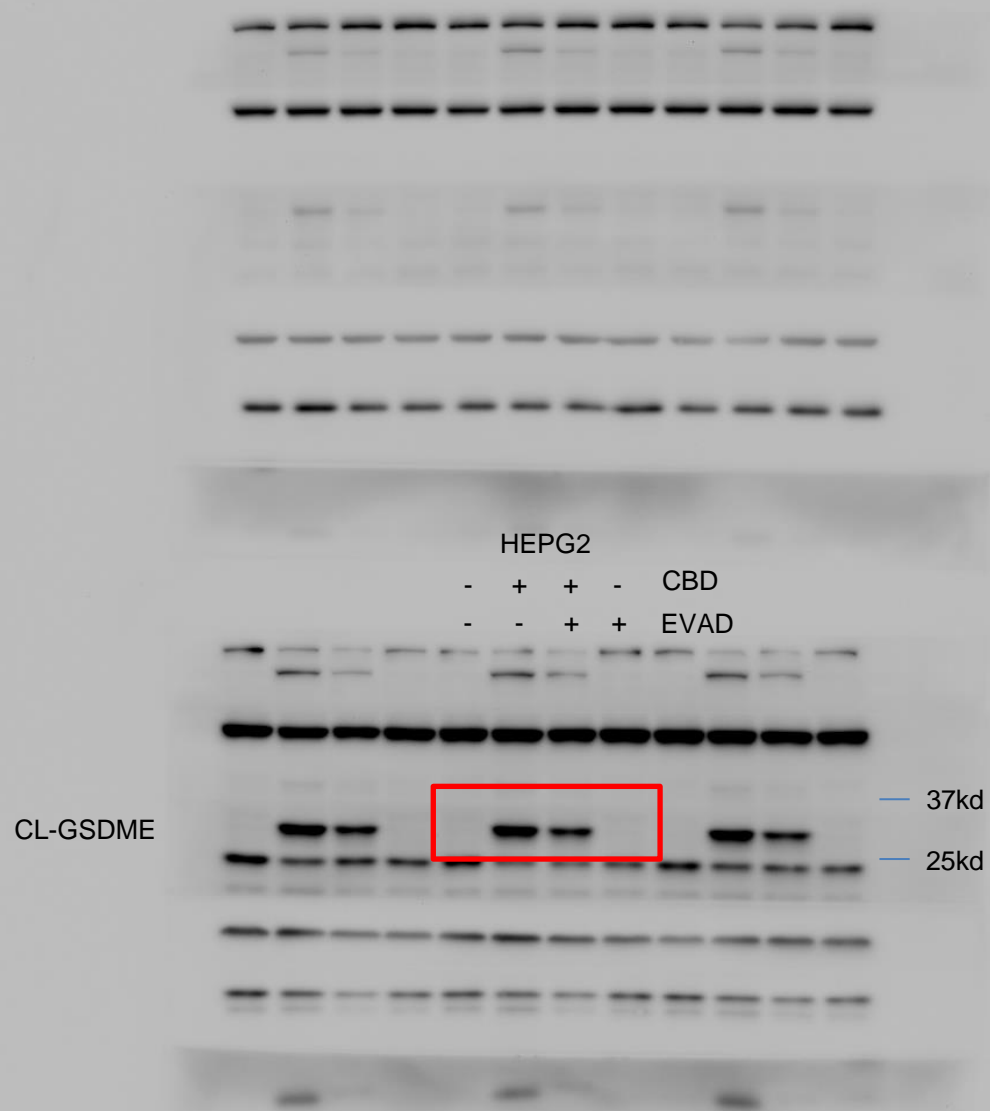

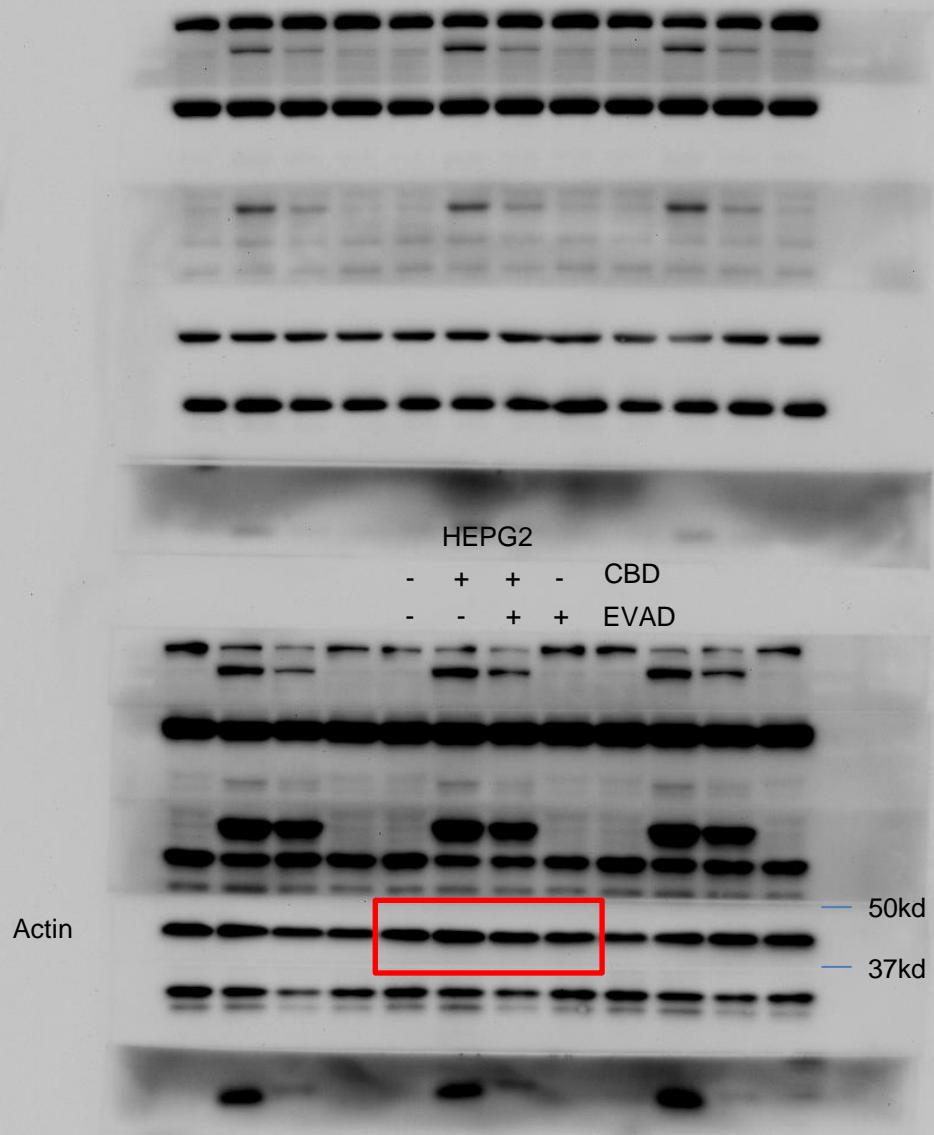

Supplement: Supplementary file 3 [file Data_Sheet_1.zip › PDF-WB-RAW-DATA/WB-Figure 3F-HEPG2.pdf]

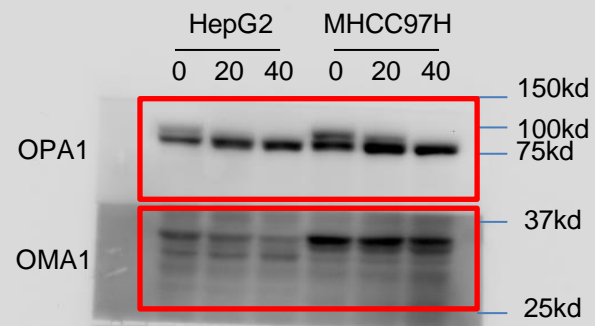

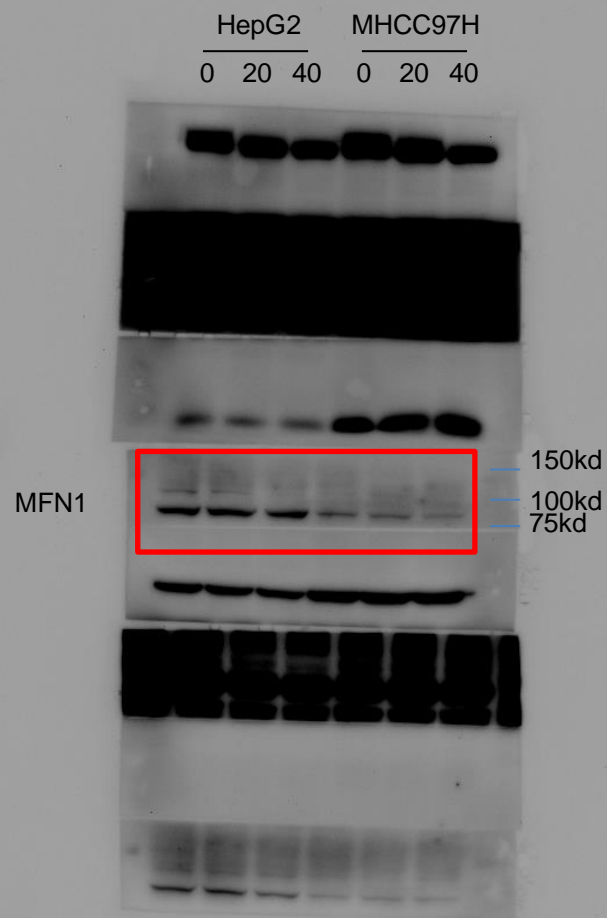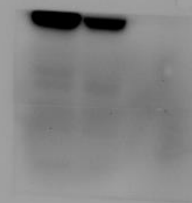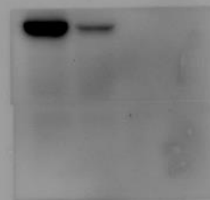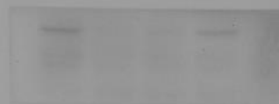

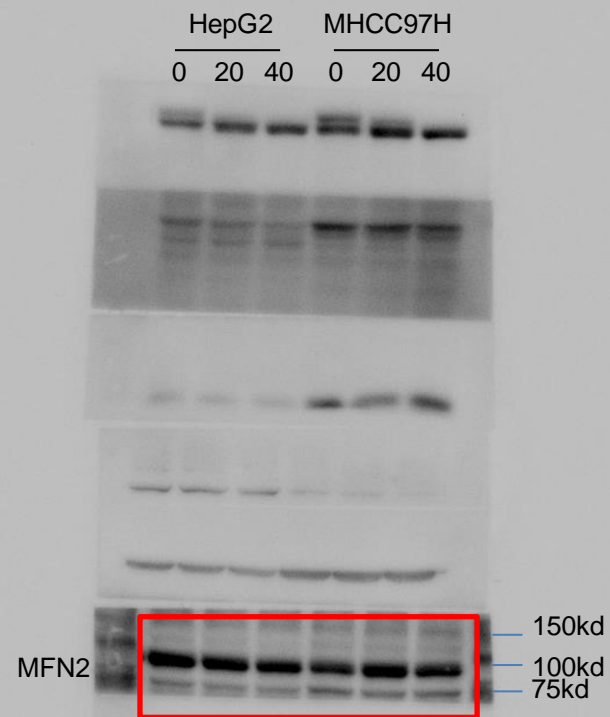

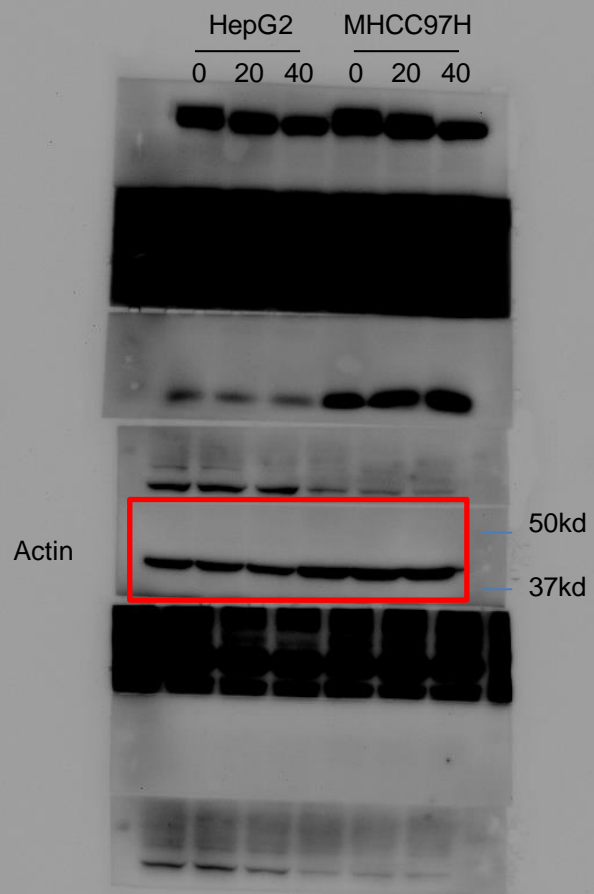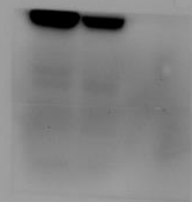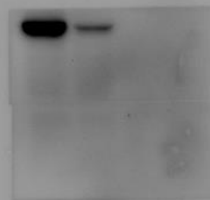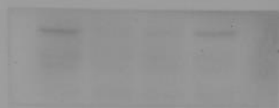

Supplement: Supplementary file 3 [file Data_Sheet_1.zip › PDF-WB-RAW-DATA/WB-Figure 4E-HEPG2 and MHCC97H.pdf]

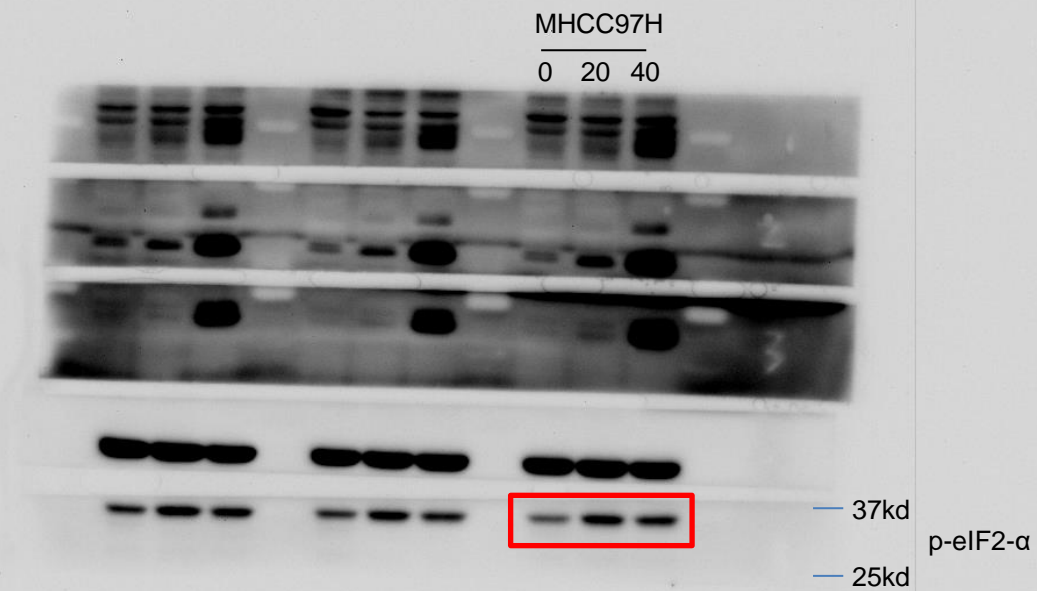

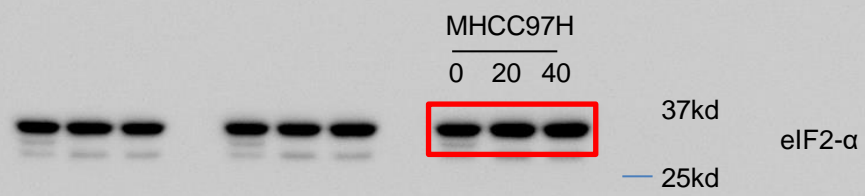

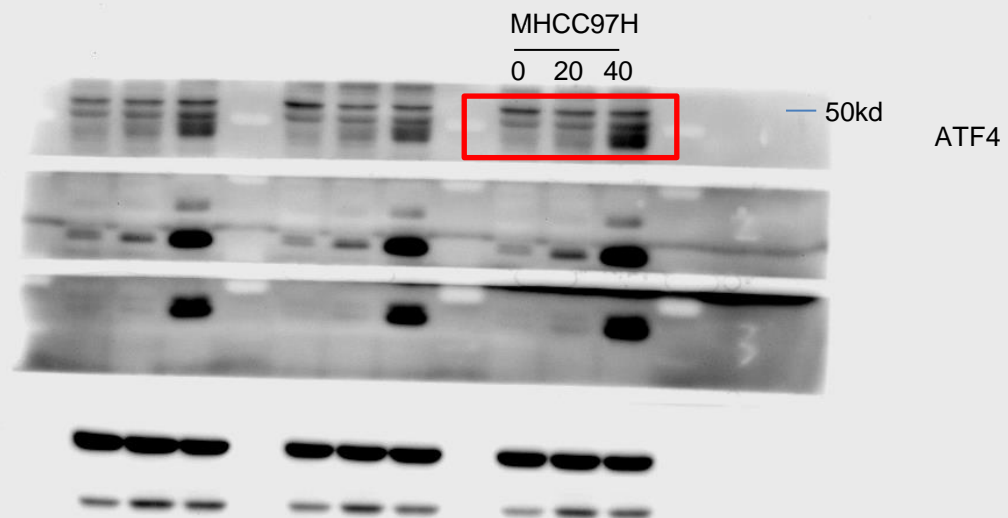

MHCC97H

0 20 40

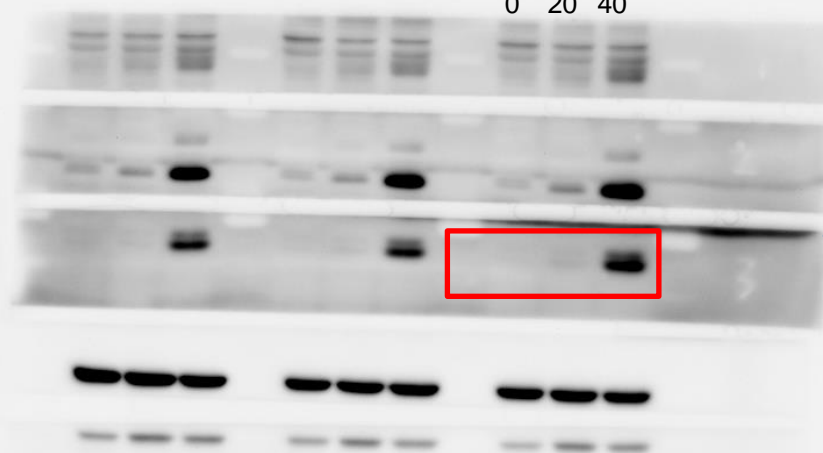

25kd

20kd

ATF3

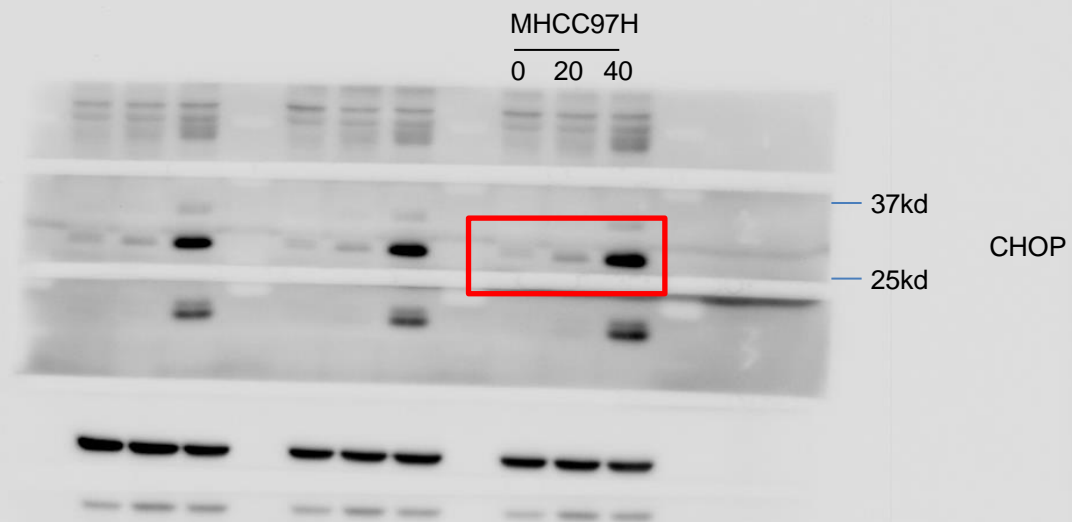

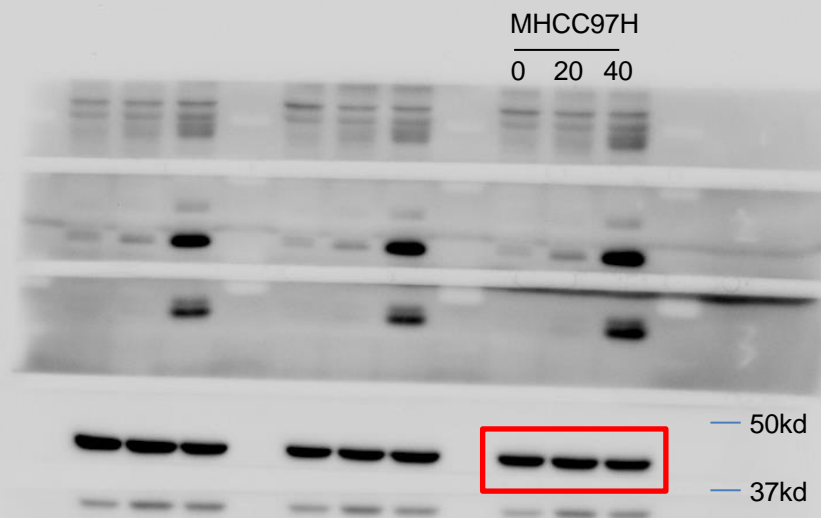

Supplement: Supplementary file 3 [file Data_Sheet_1.zip › PDF-WB-RAW-DATA/WB-Figure 5A- MHCC97H.pdf]

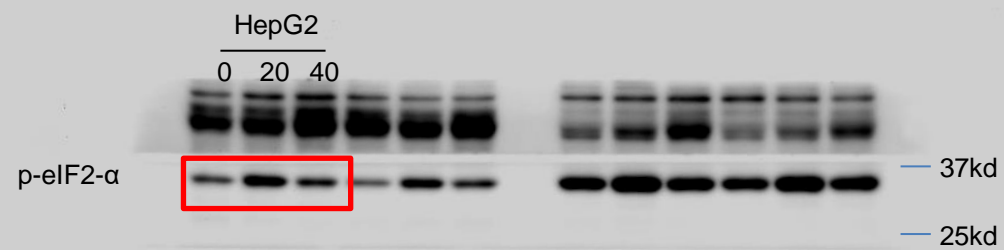

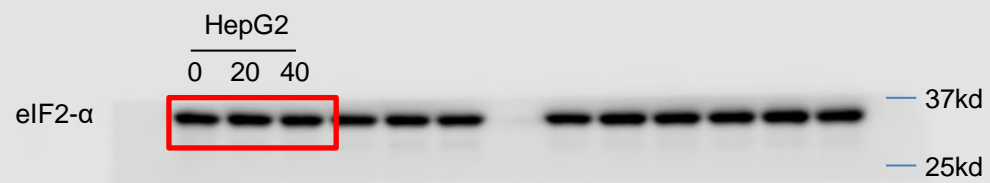

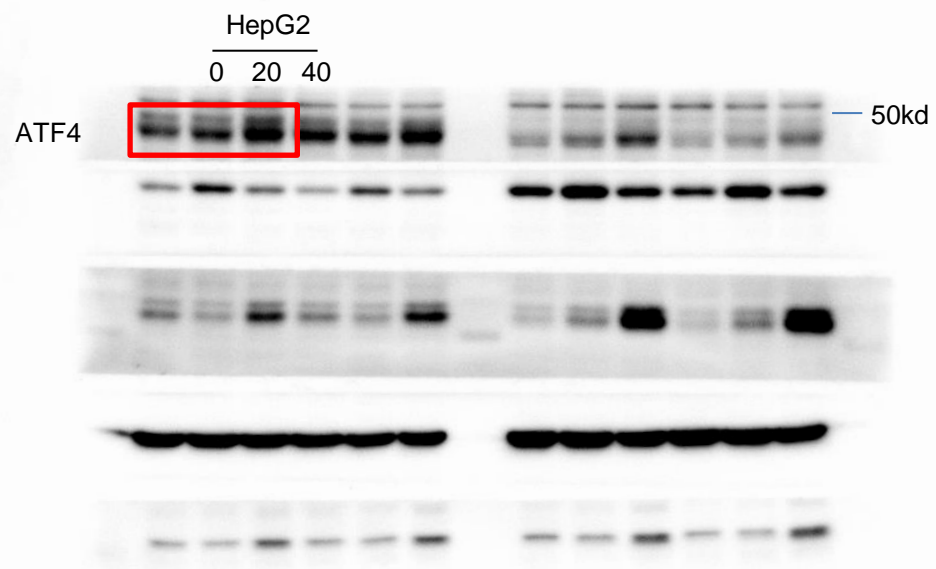

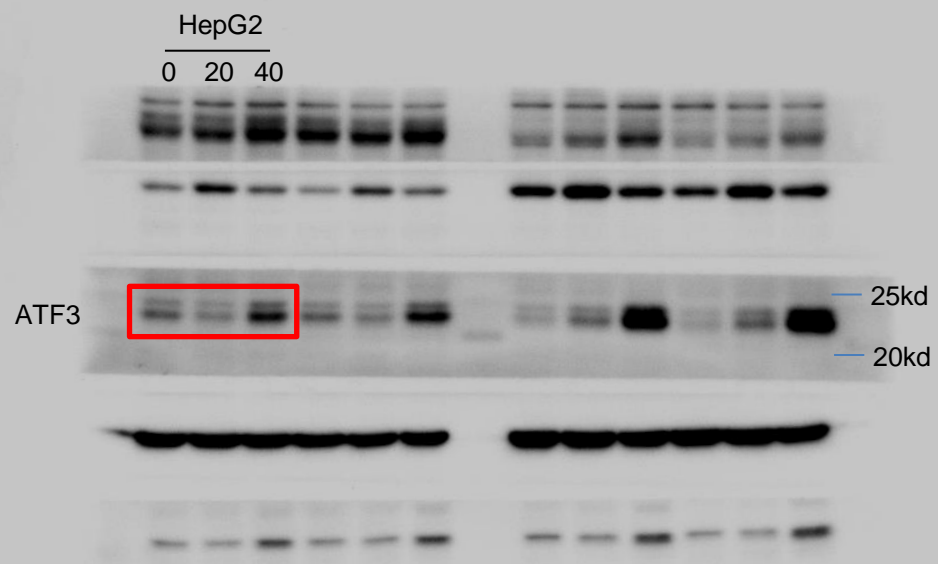

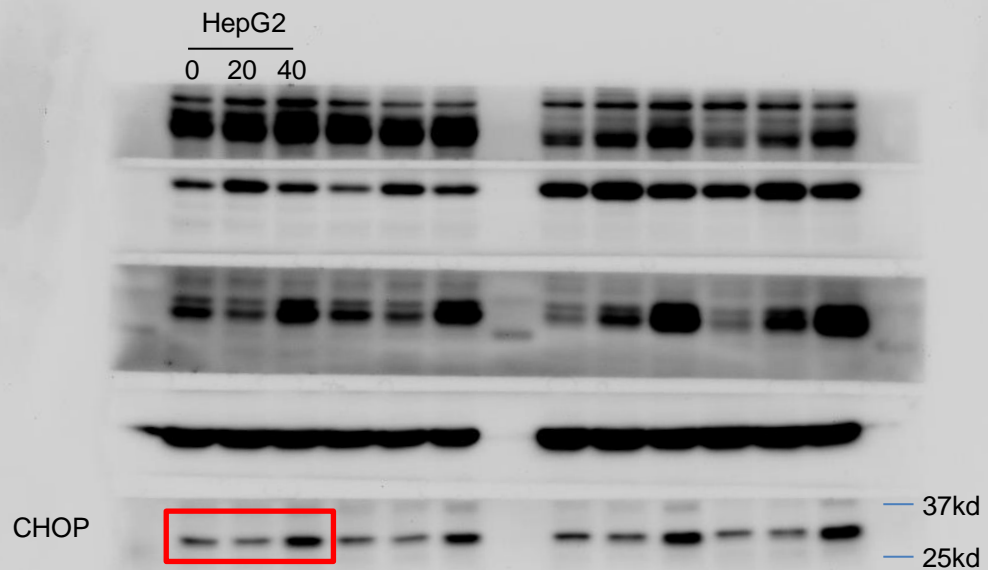

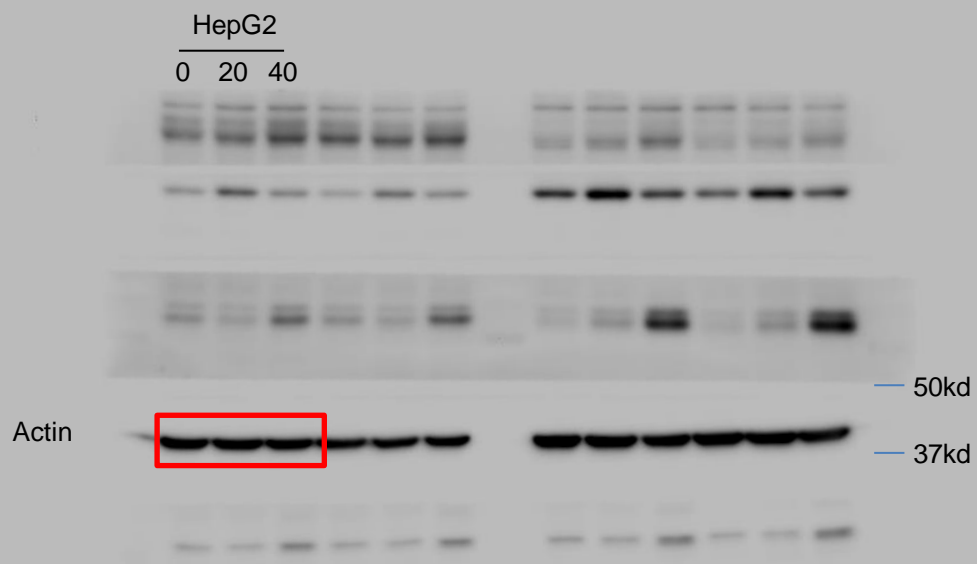

Supplement: Supplementary file 3 [file Data_Sheet_1.zip › PDF-WB-RAW-DATA/WB-Figure 5A-HEPG2.pdf]

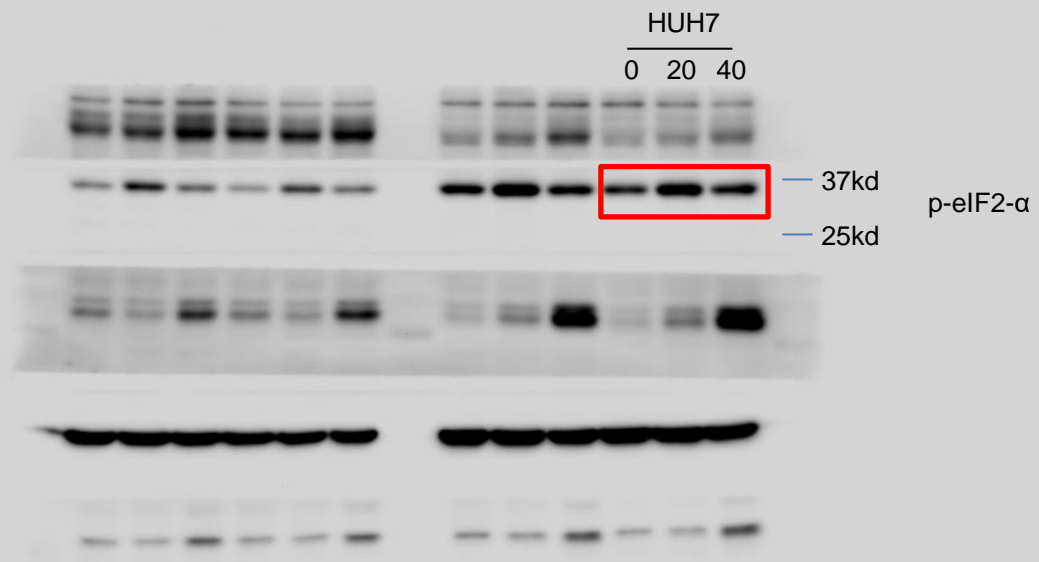

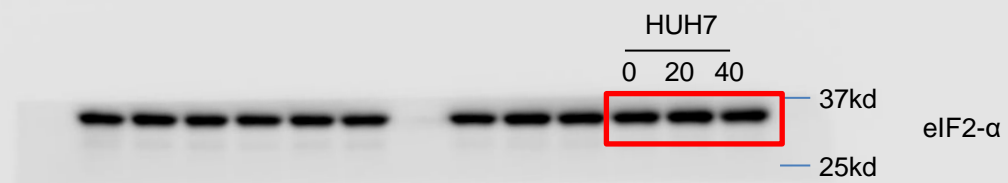

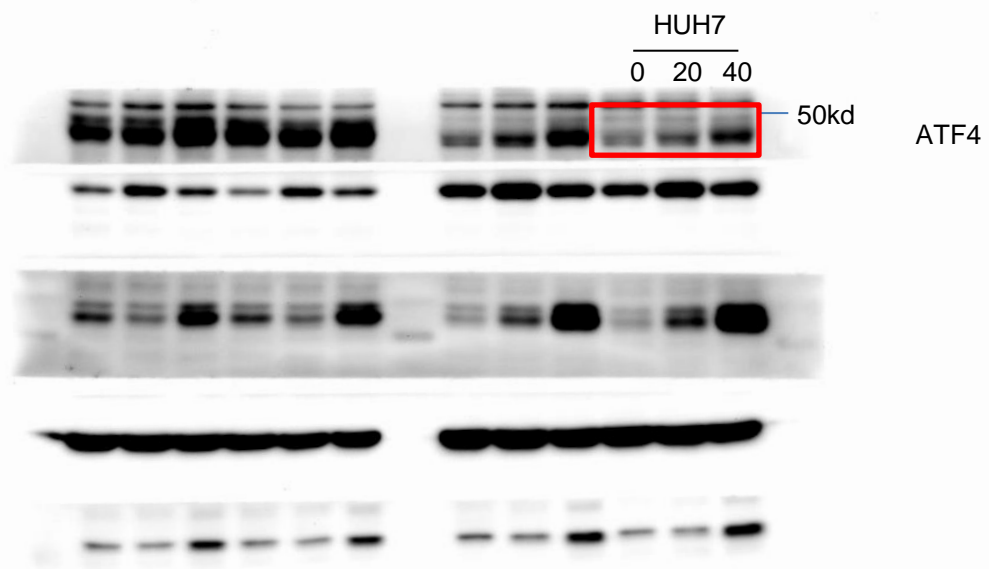

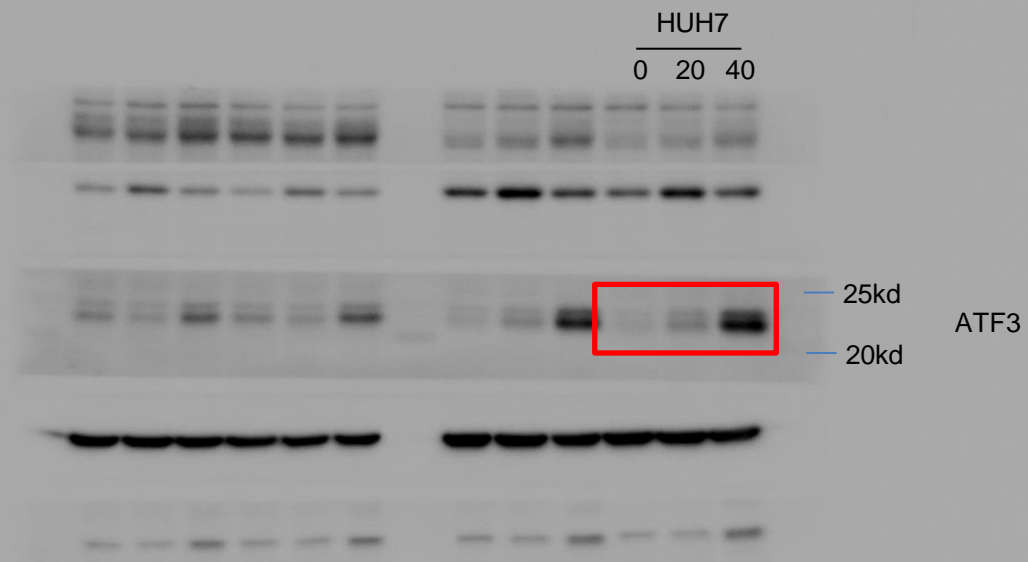

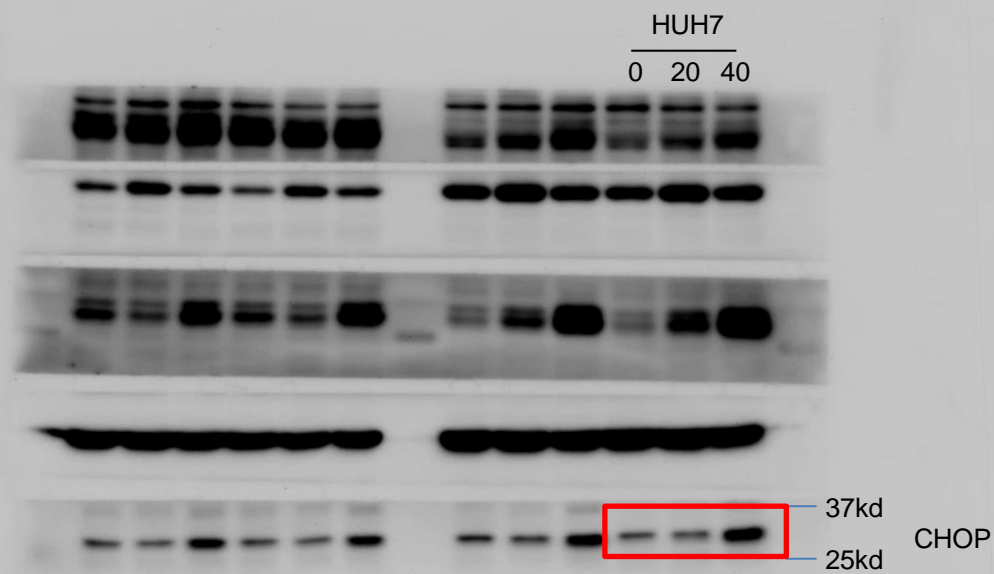

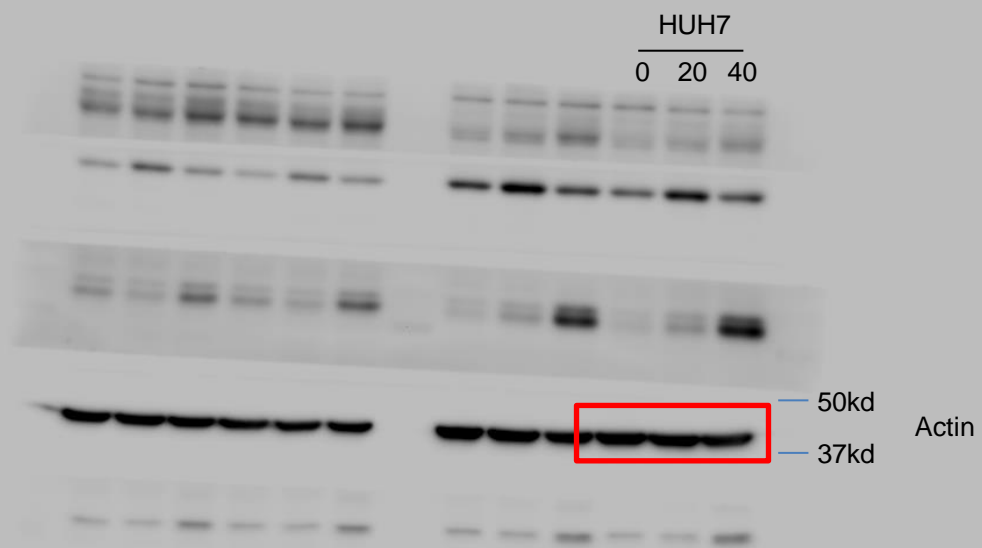

Supplement: Supplementary file 3 [file Data_Sheet_1.zip › PDF-WB-RAW-DATA/WB-Figure 5A-huh7.pdf]

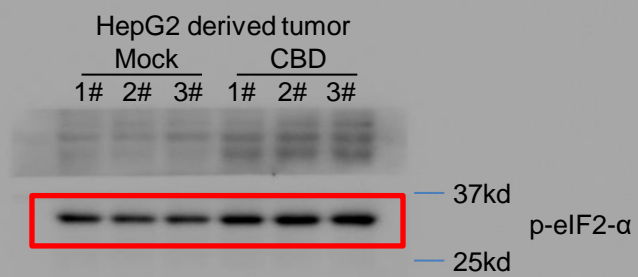

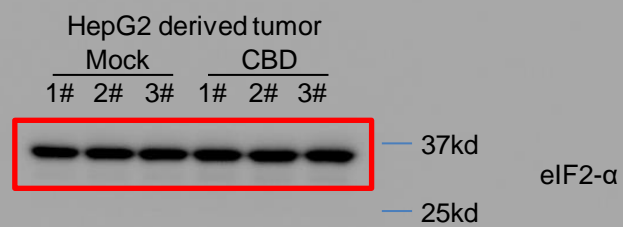

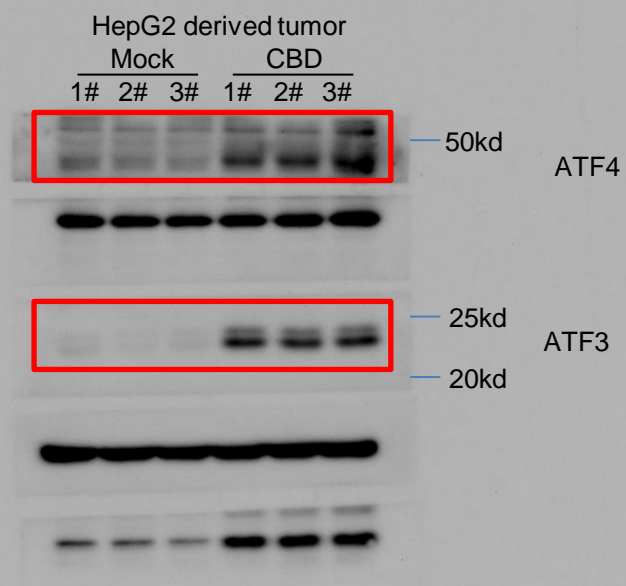

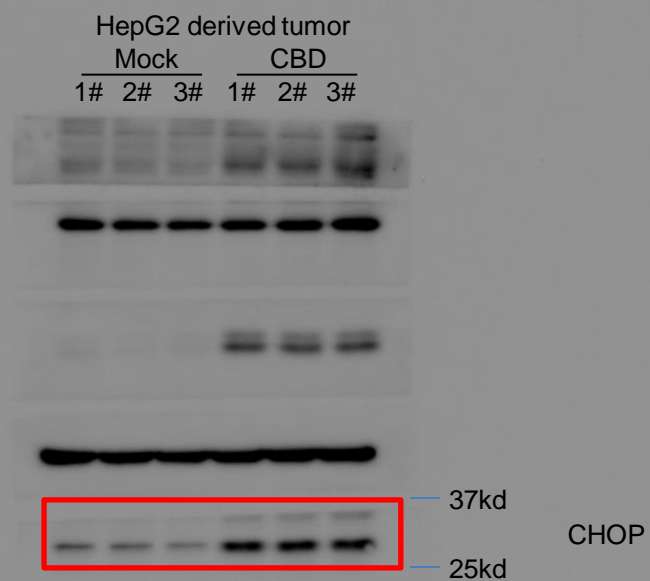

1# 2# 3# 1# 2# 3#

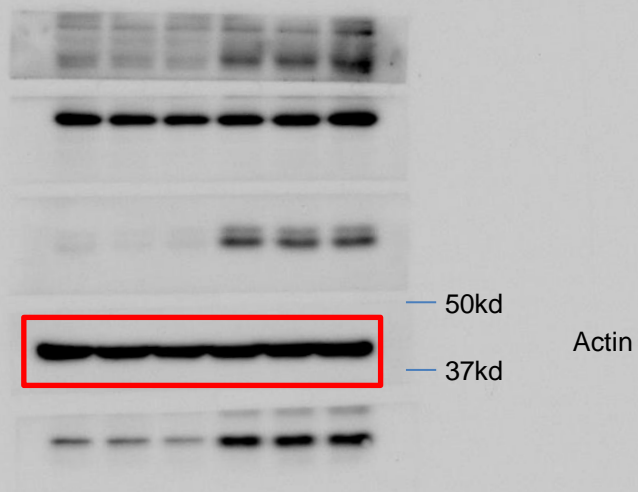

Supplement: Supplementary file 3 [file Data_Sheet_1.zip › PDF-WB-RAW-DATA/WB-Figure 5B.pdf]

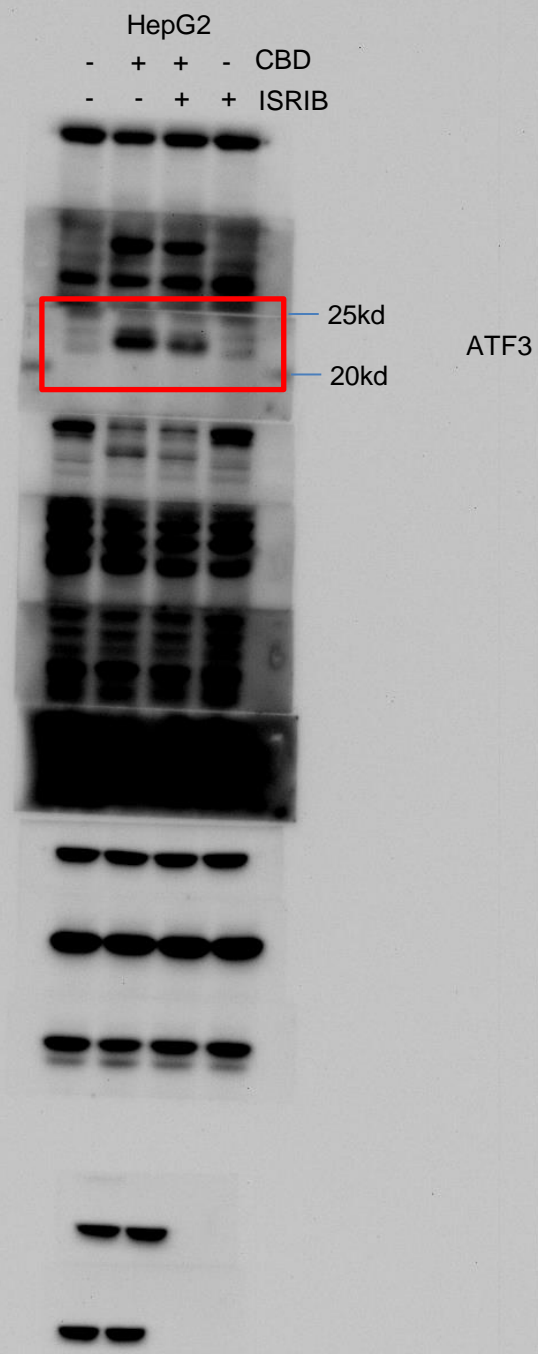

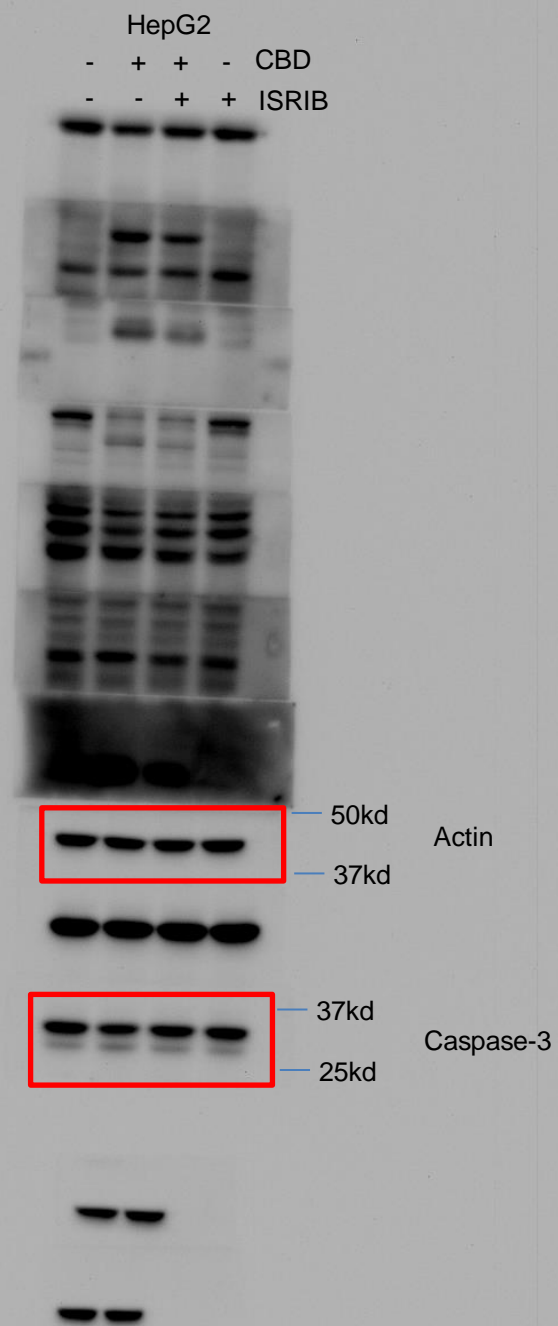

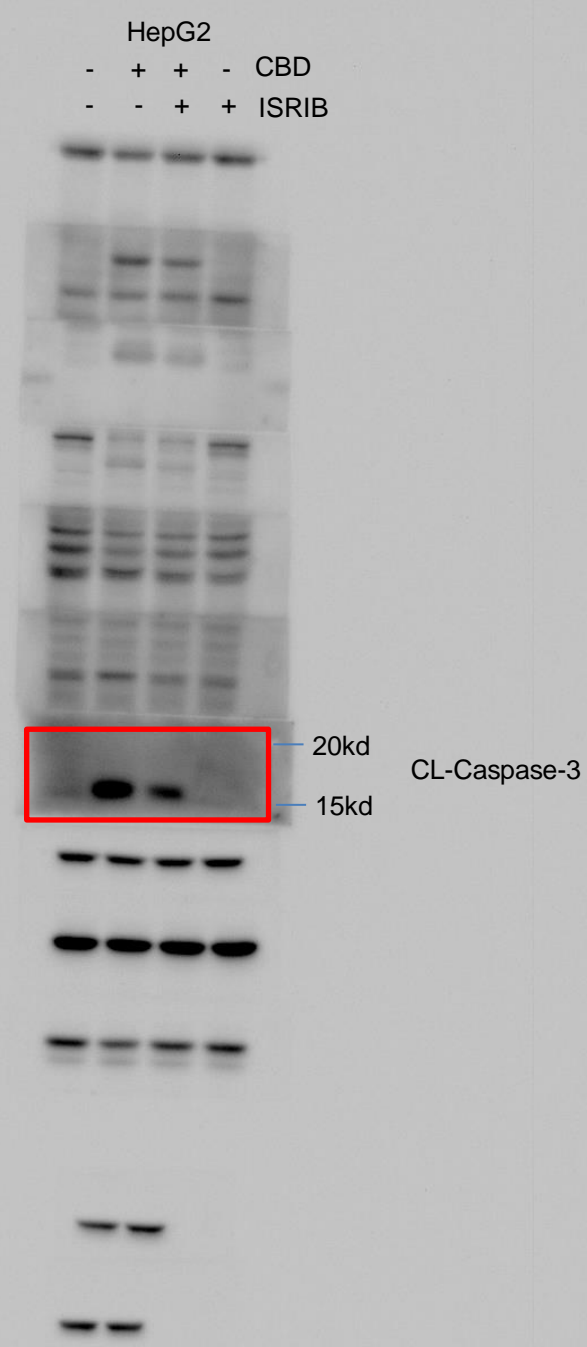

HepG2

- + + - CBD  
- - + + ISRIB

50kd GSDME-L

37kd GSDME-S  
25kd

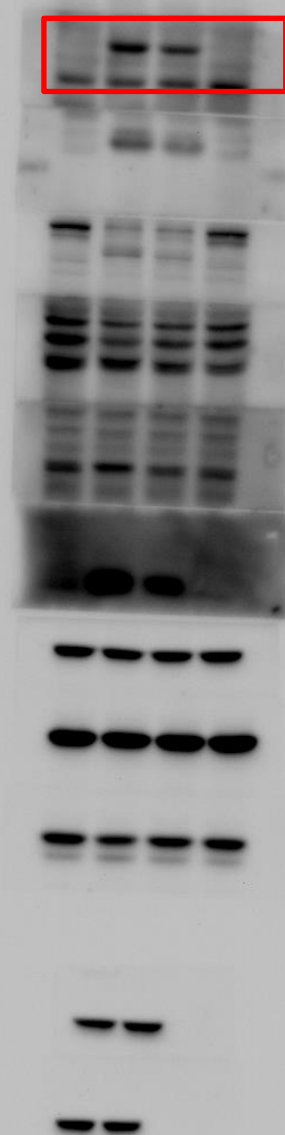

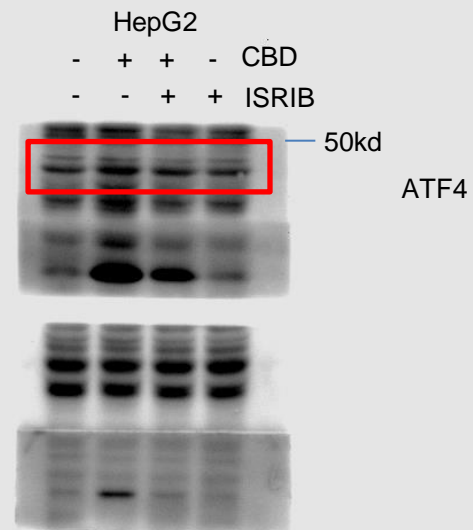

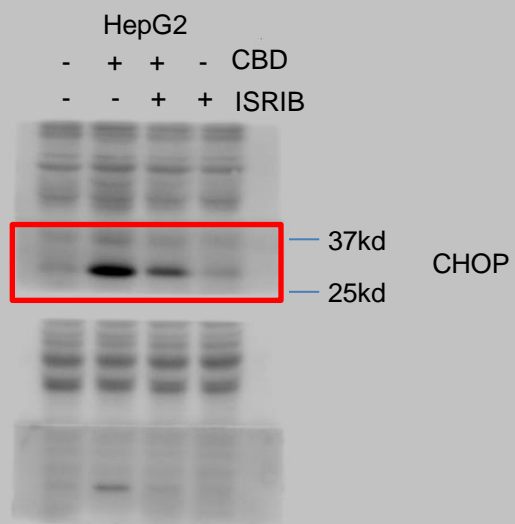

Supplement: Supplementary file 3 [file Data_Sheet_1.zip › PDF-WB-RAW-DATA/WB-Figure 5F-HEPG2.pdf]

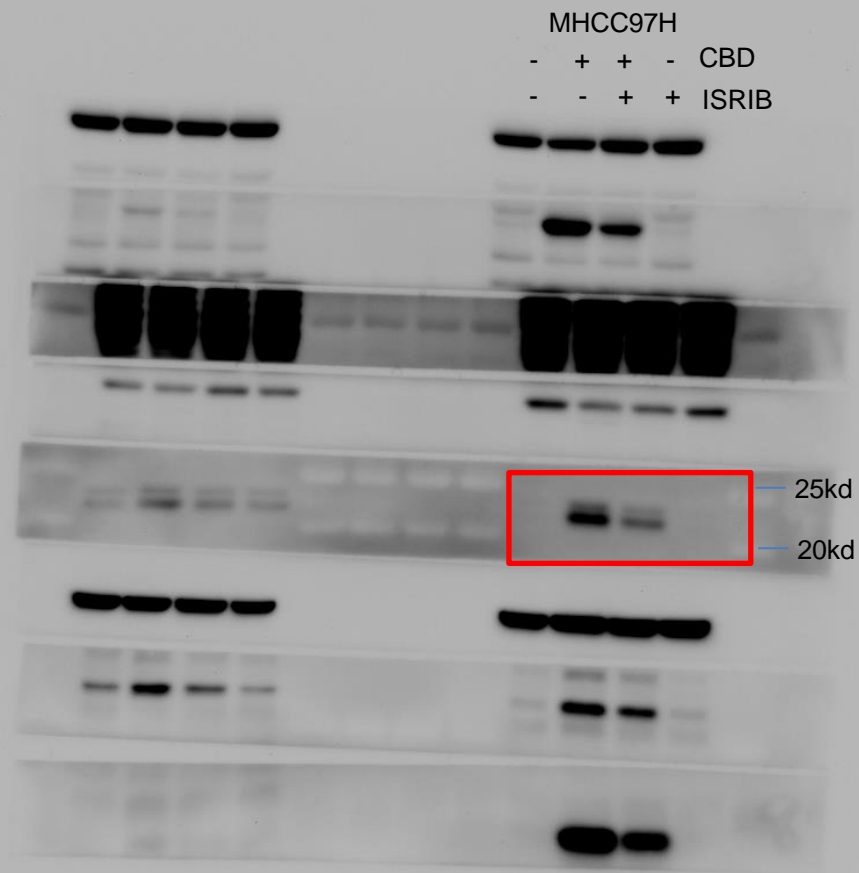

ATF3

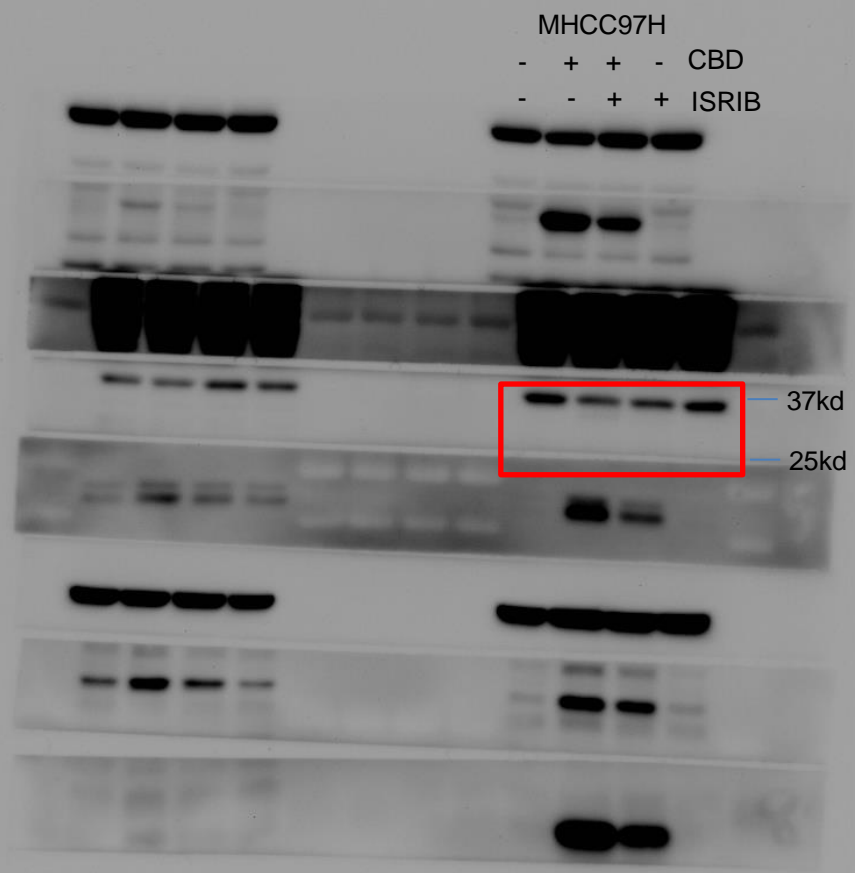

Caspase-3

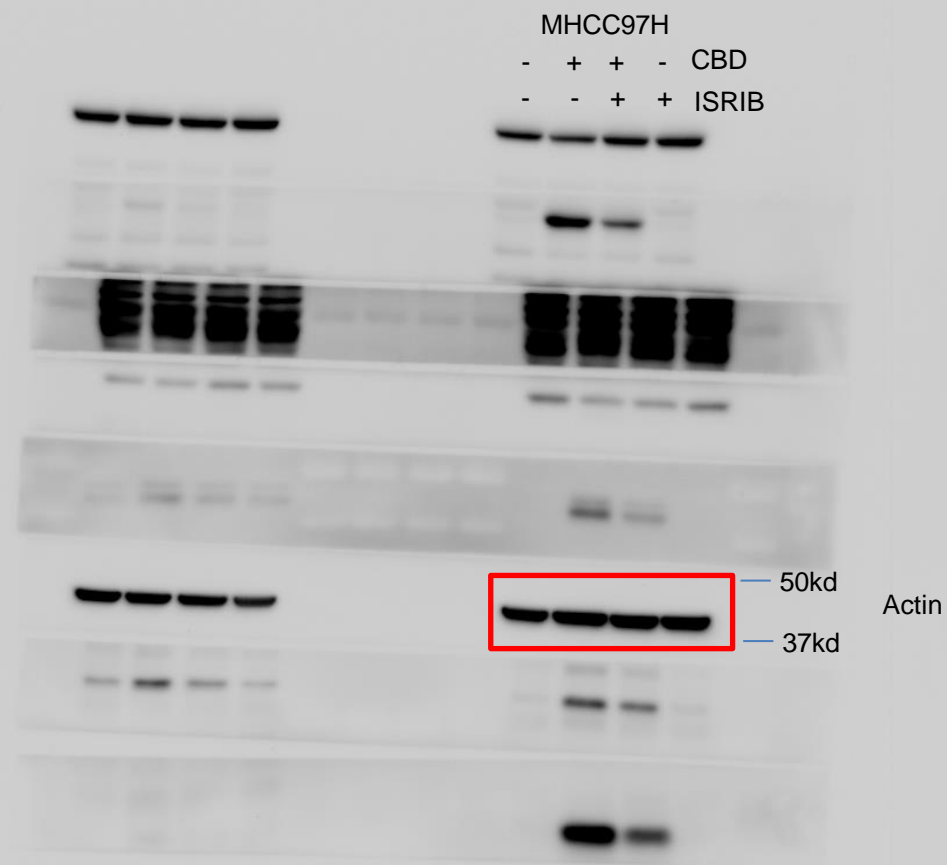

MHCC97H

- + + - CBD  
- - + + ISRIB

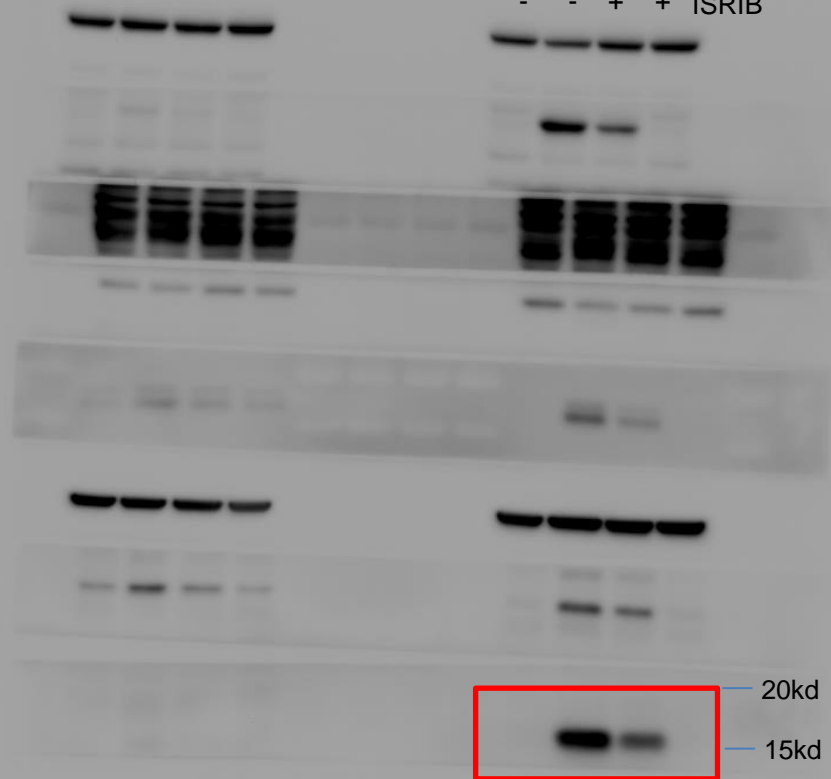

20kd

CL-Caspase-3

15kd

MHCC97H

- + + - CBD  
- - + + ISRIB

50kd GSDME-L

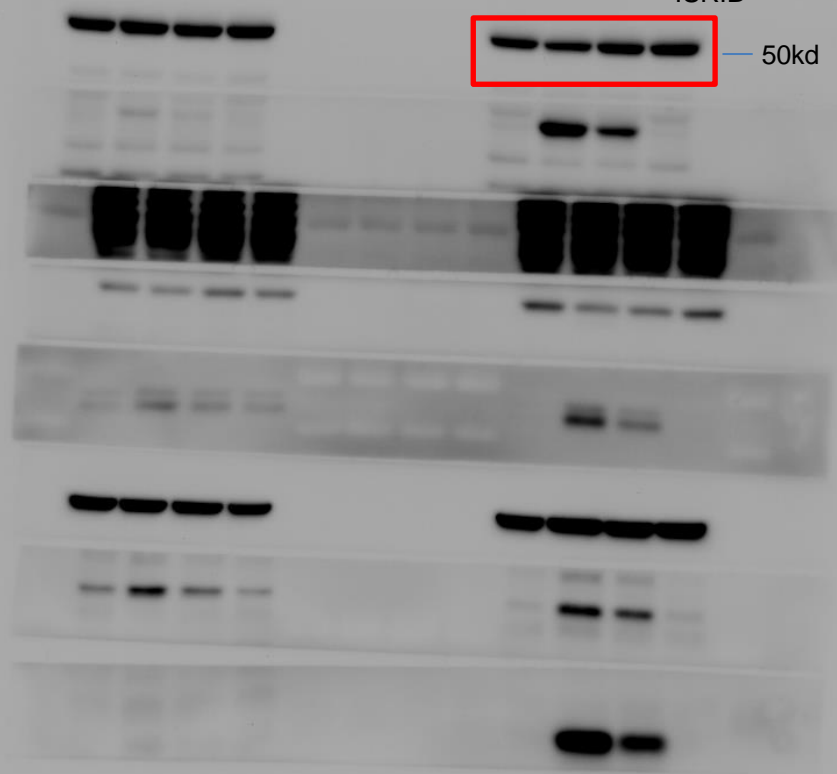

MHCC97H

- + + - CBD  
- - + + ISRIB

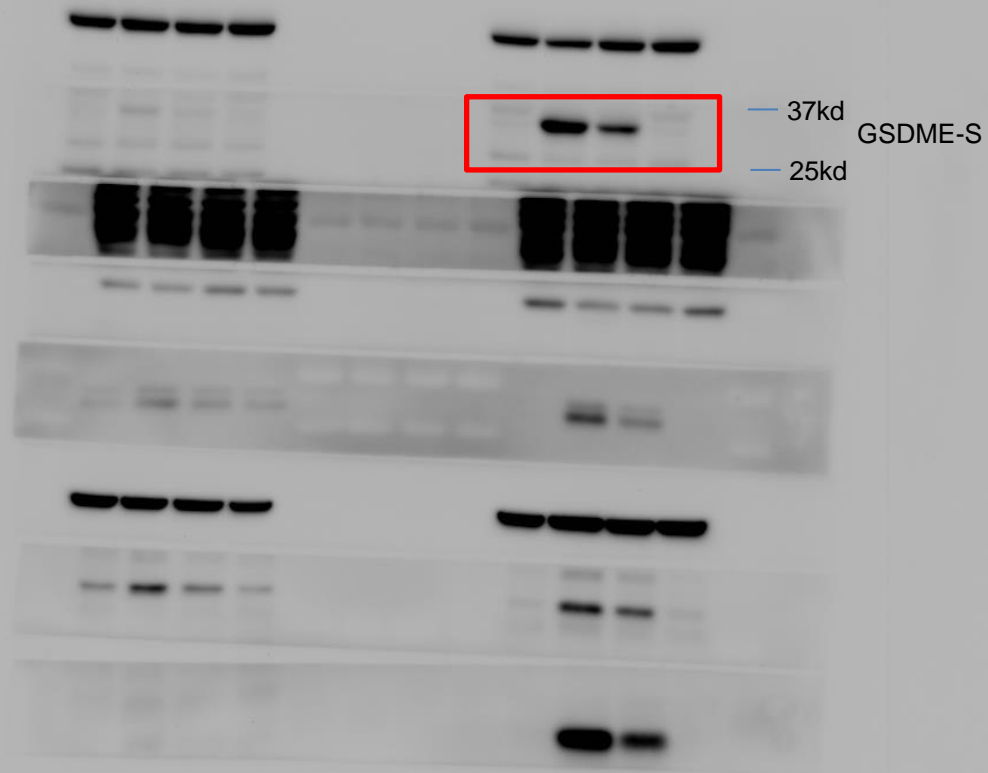

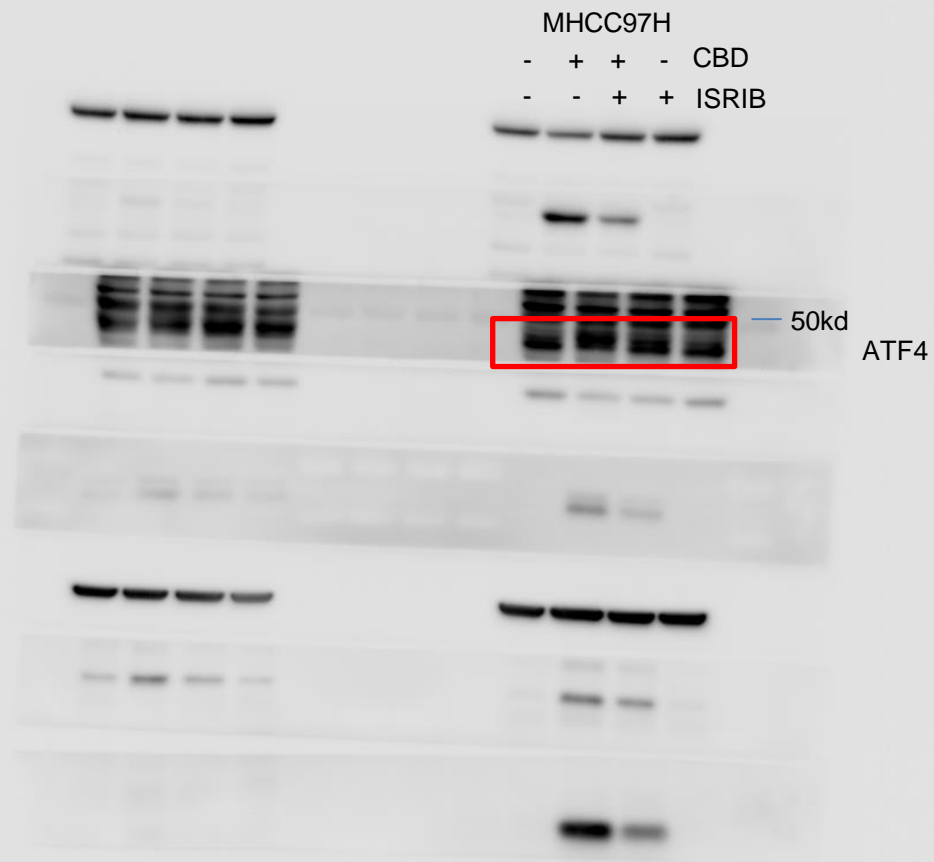

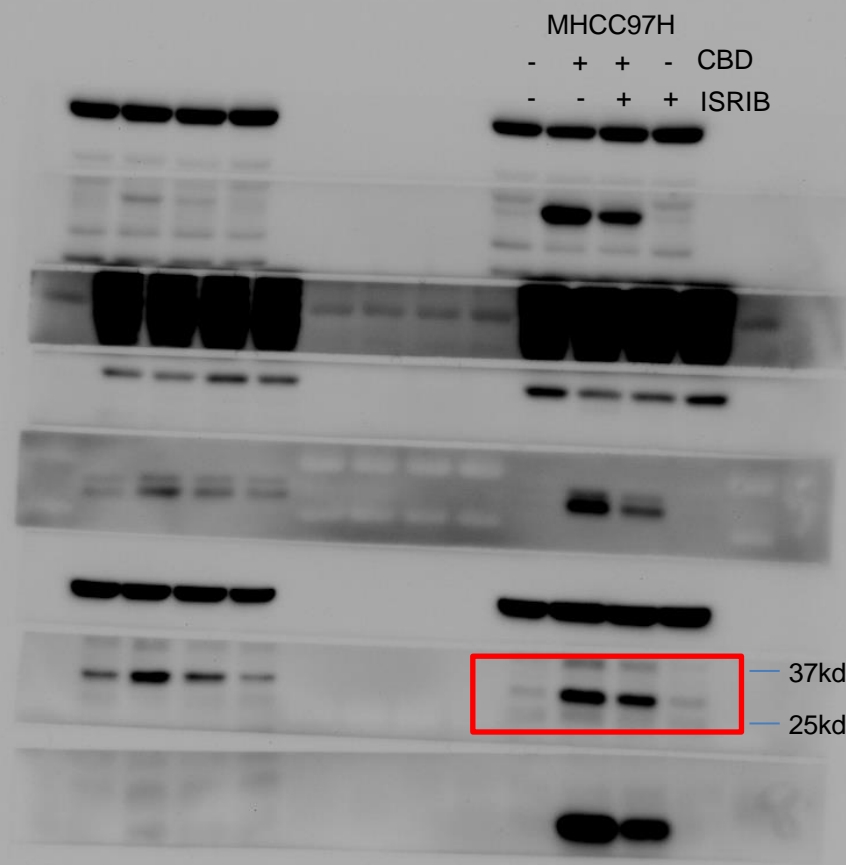

Supplement: Supplementary file 3 [file Data_Sheet_1.zip › PDF-WB-RAW-DATA/WB-Figure 5F-MHCC97H.pdf]

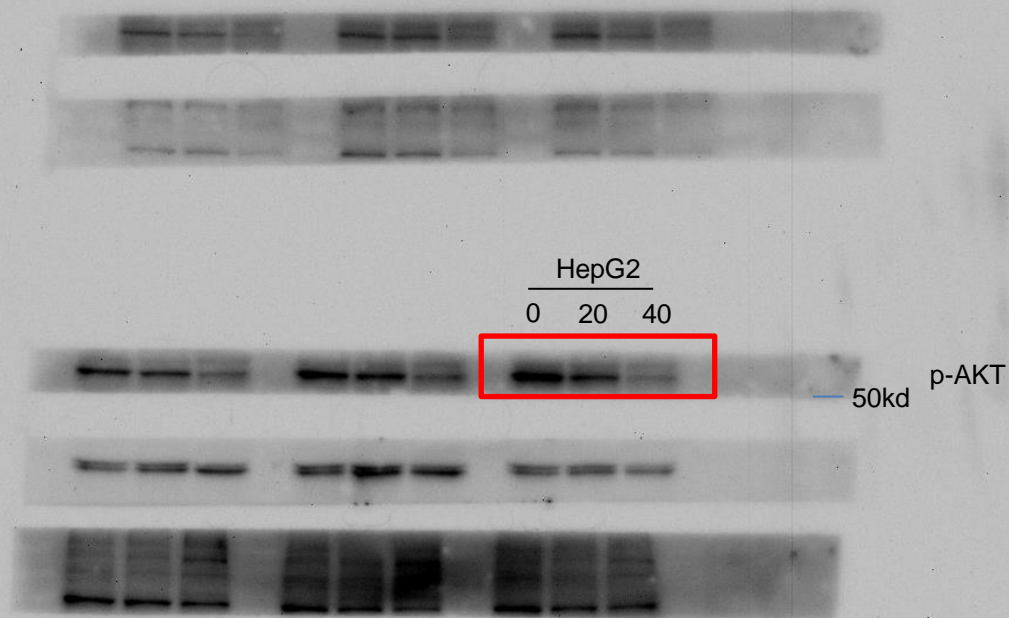

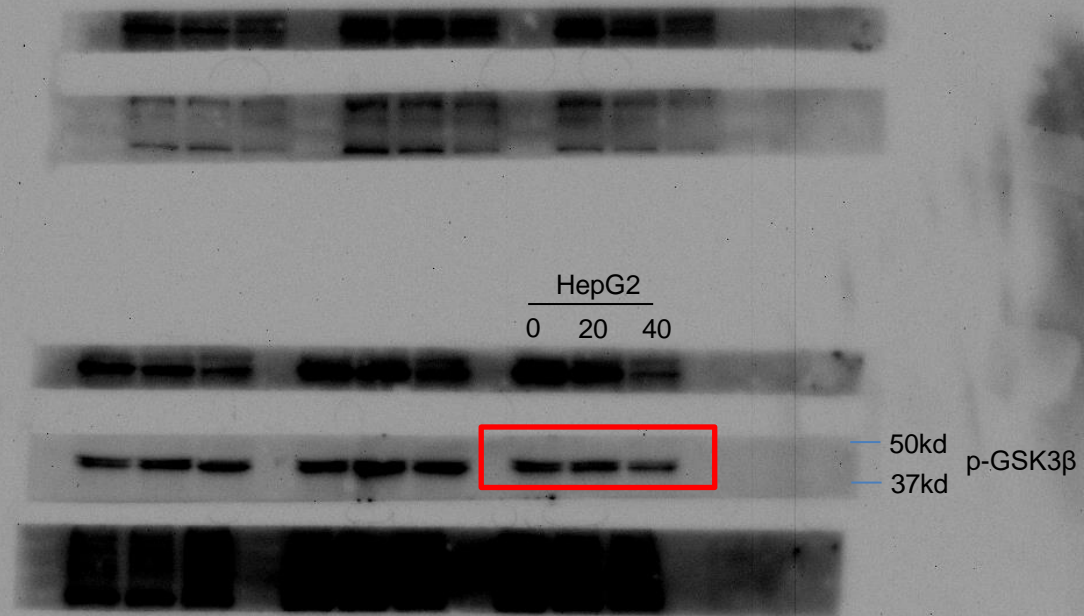

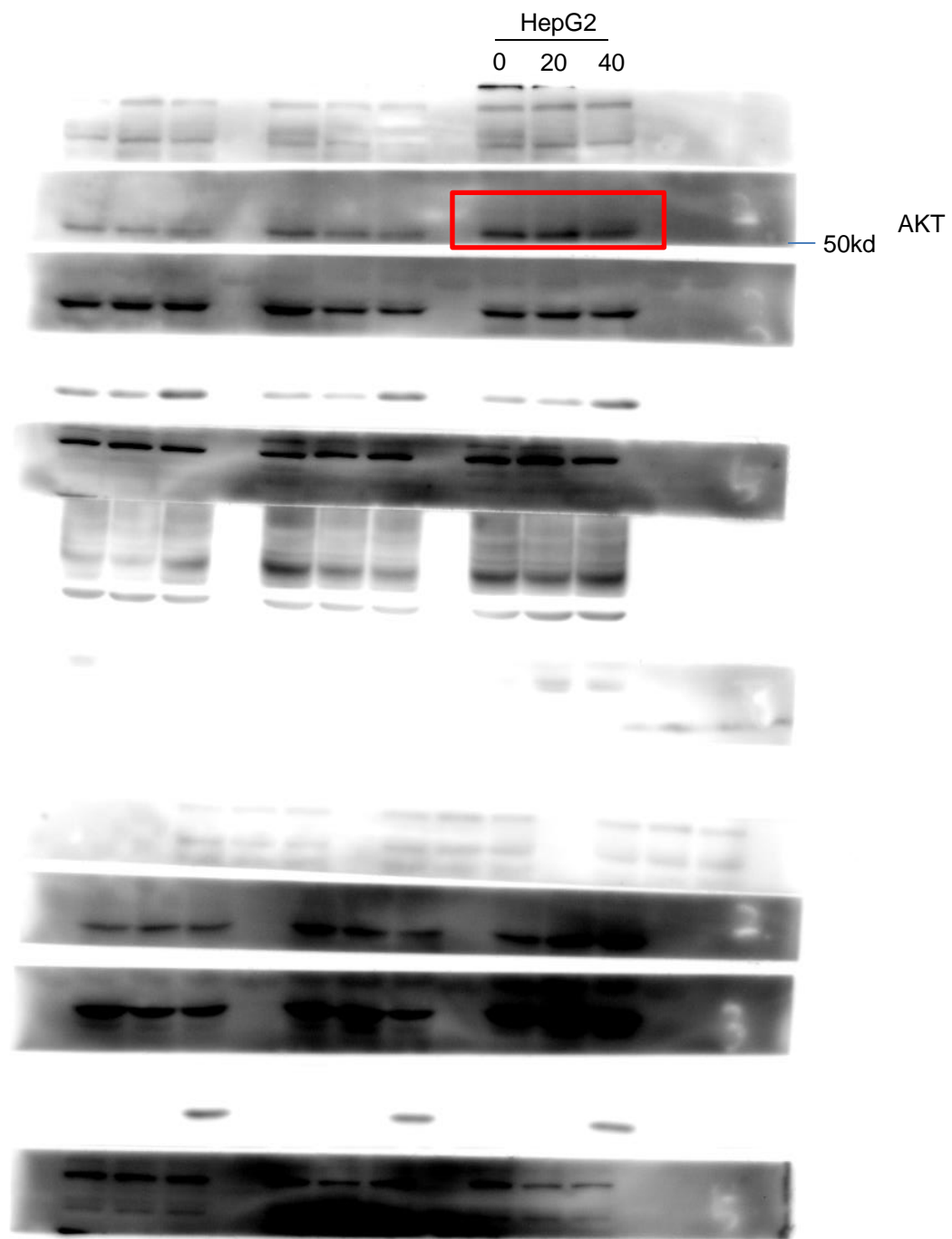

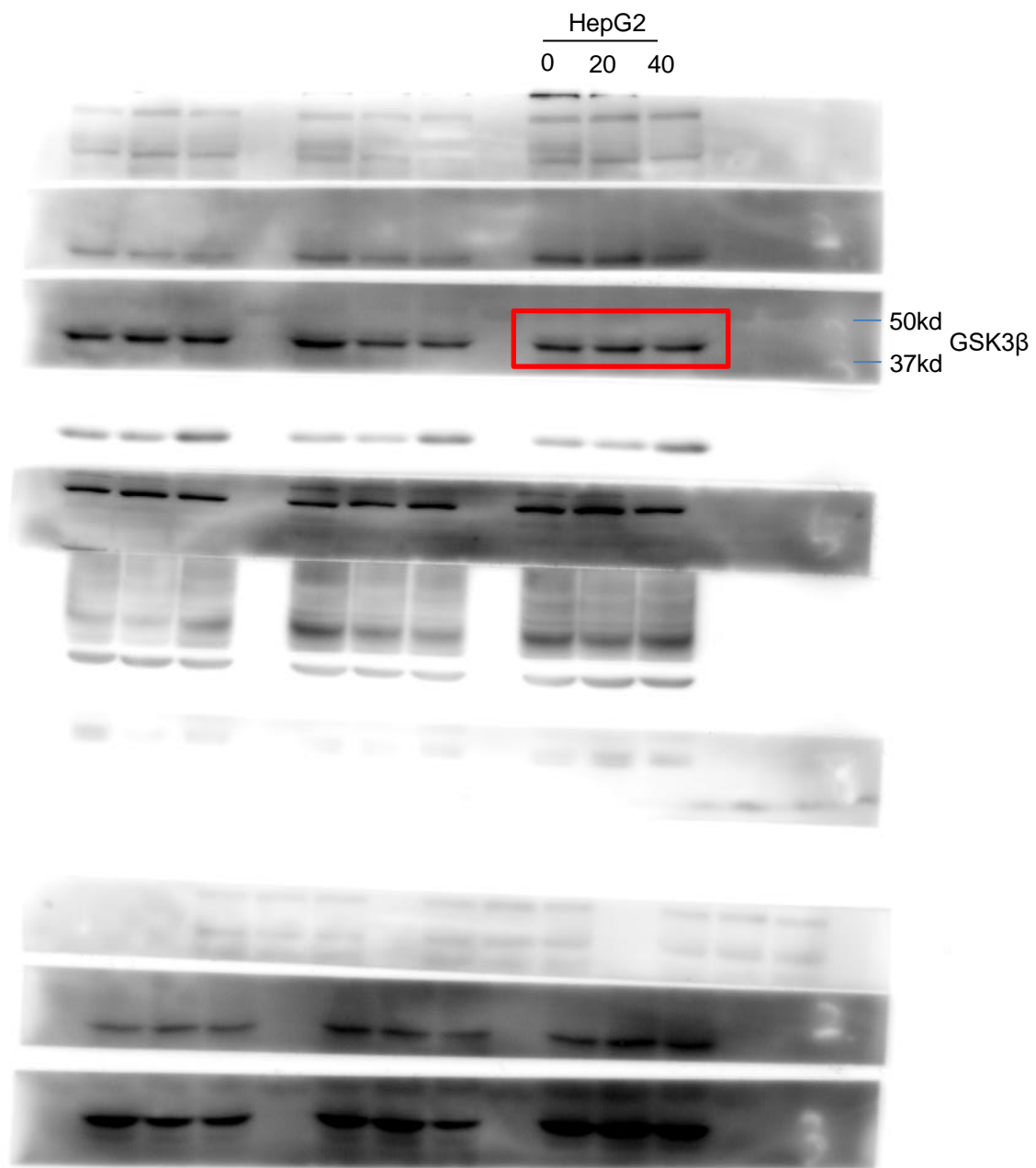

HepG2  
0 20 40

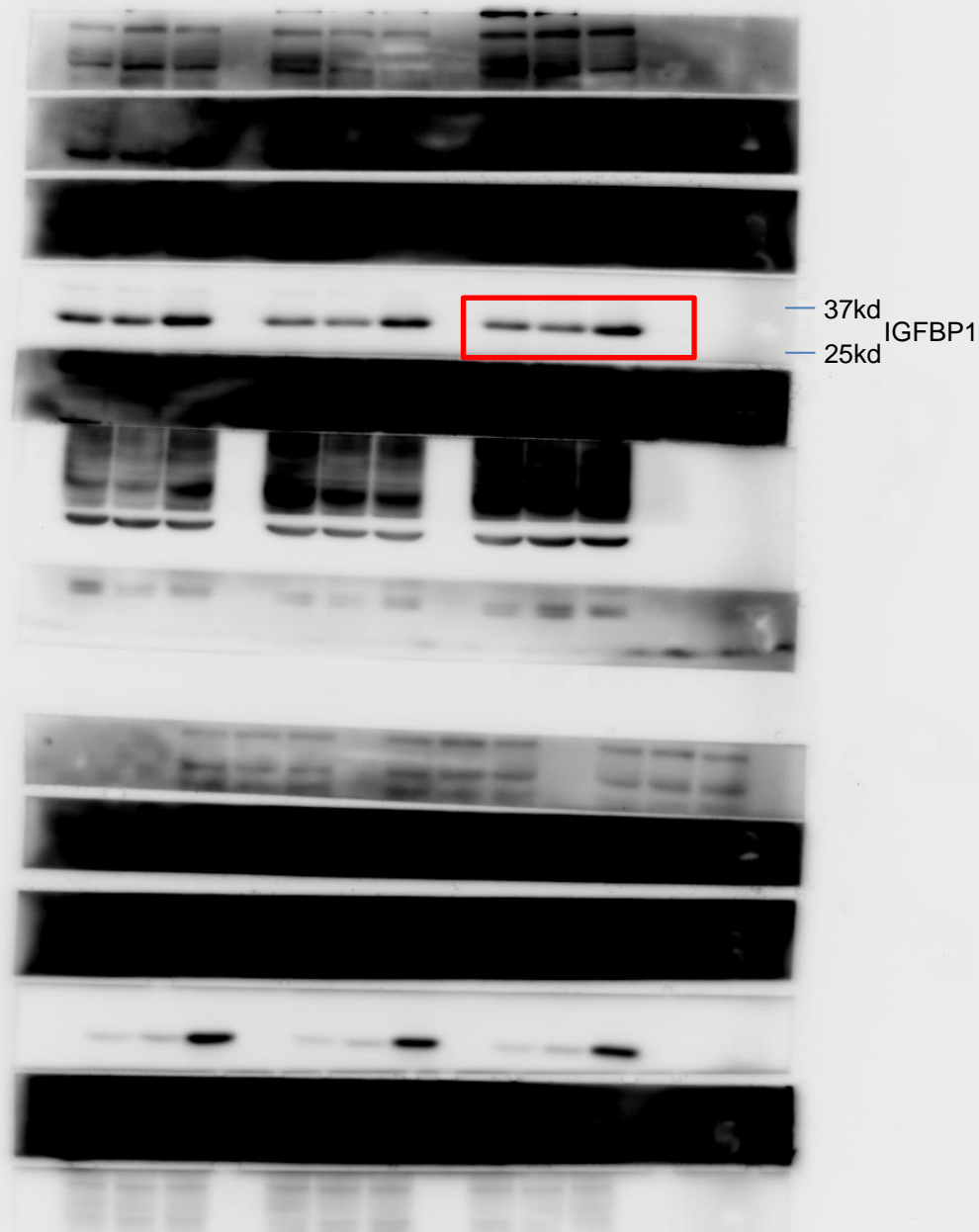

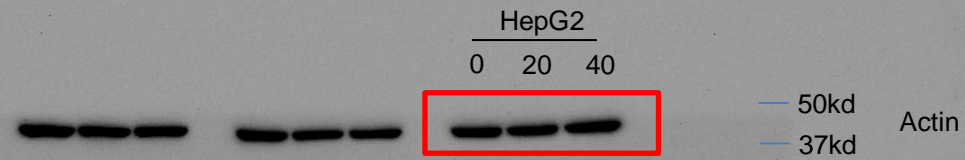

Supplement: Supplementary file 3 [file Data_Sheet_1.zip › PDF-WB-RAW-DATA/WB-Figure 6C-HepG2.pdf]

MHCC97H

0 20 40

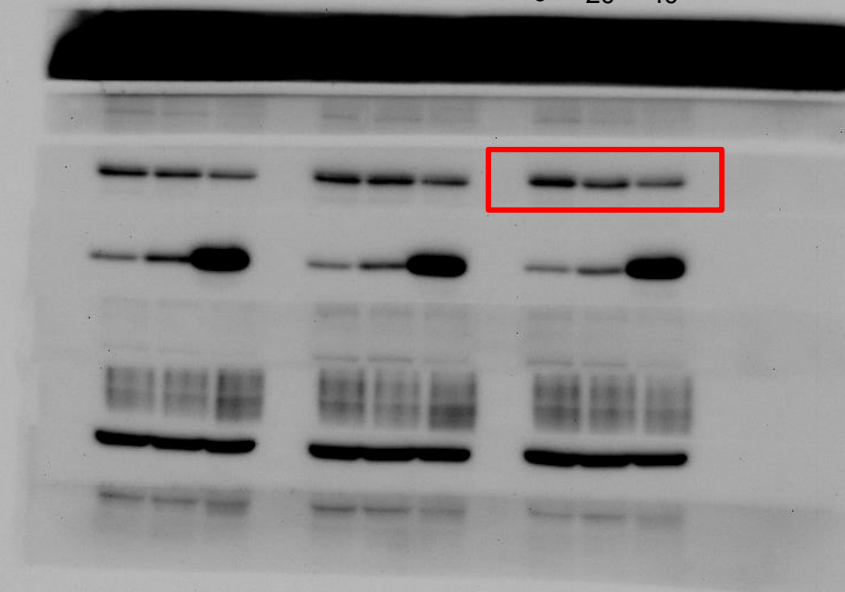

50kd  
37kd p-GSK3β

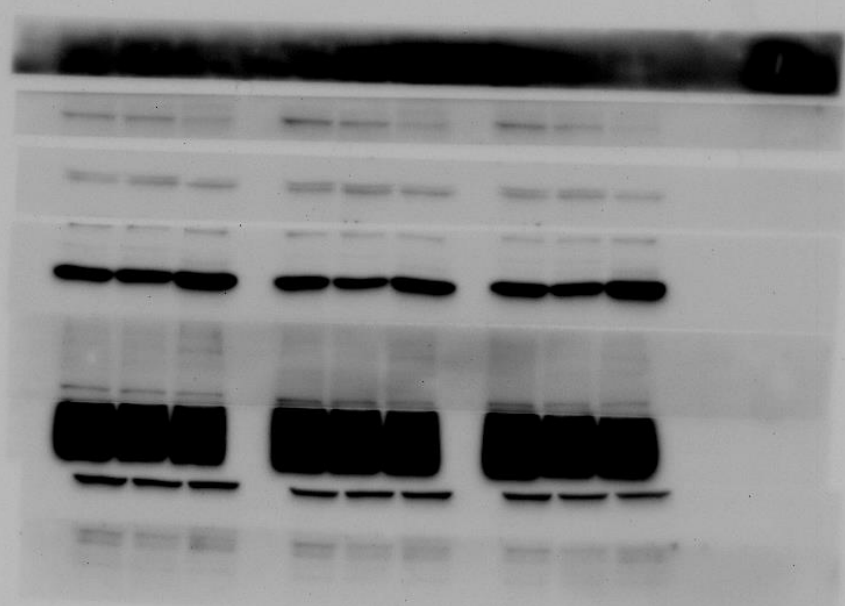

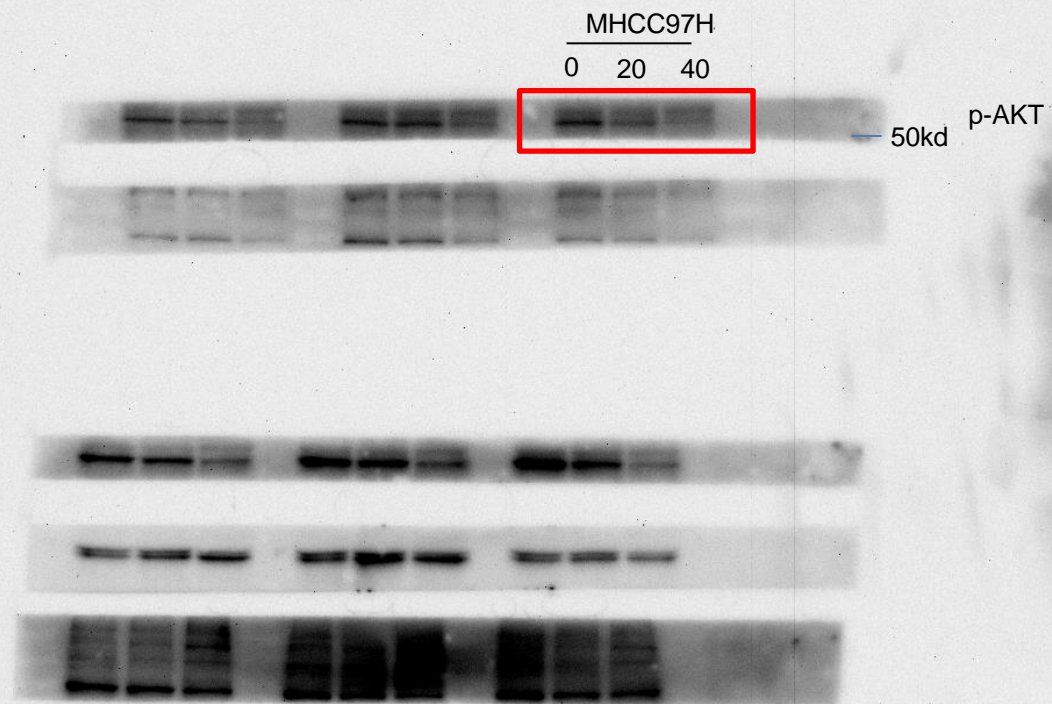

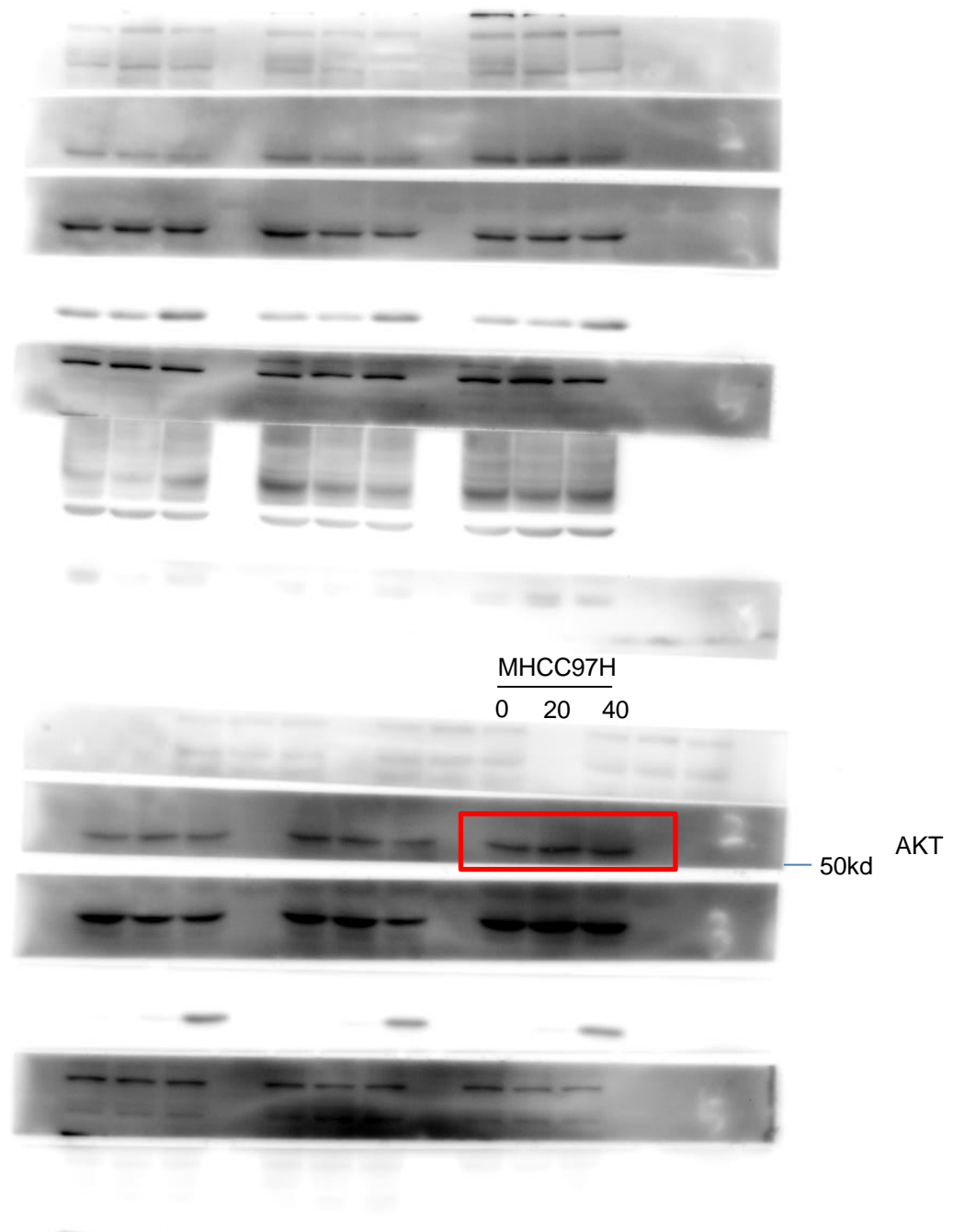

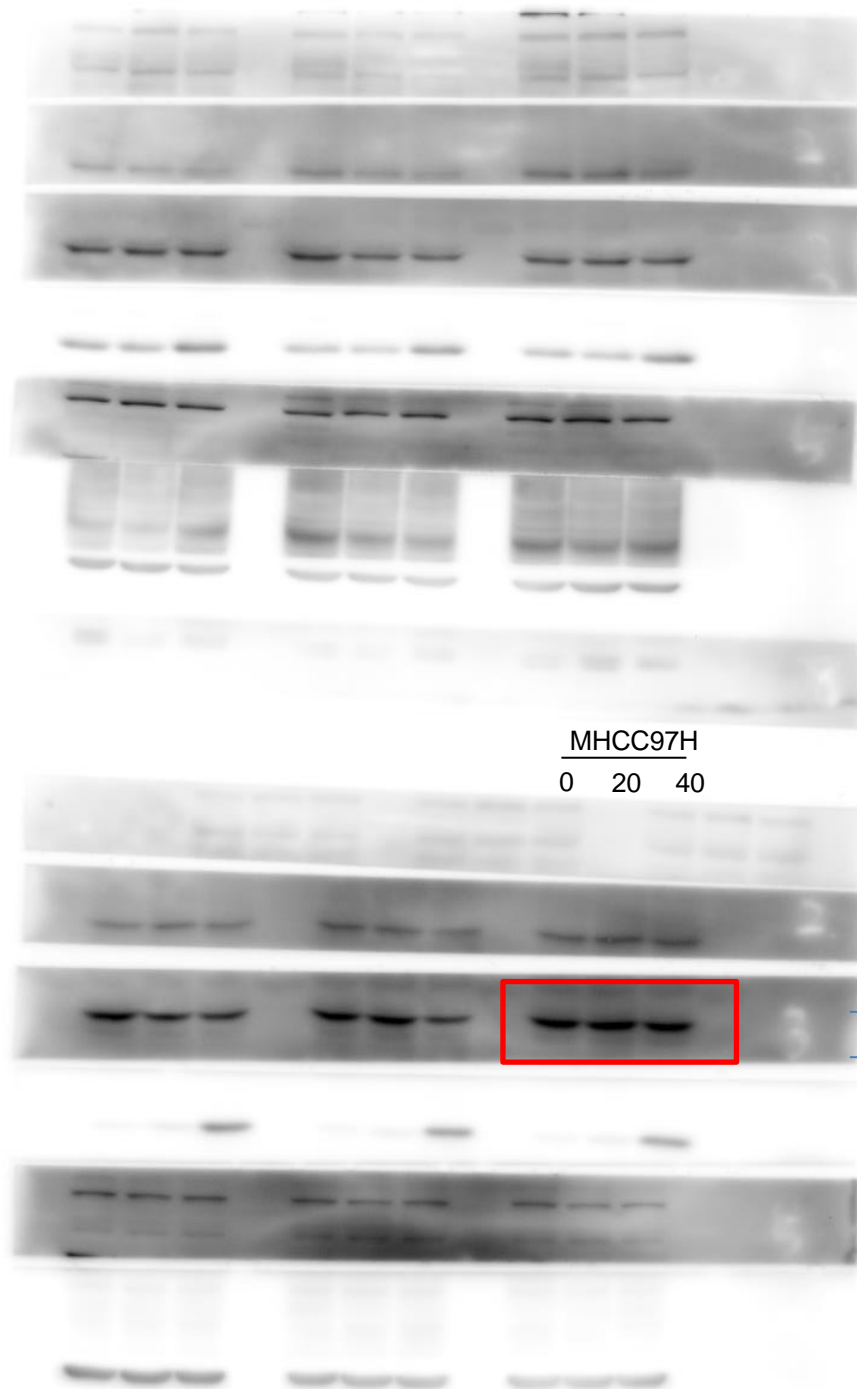

MHCC97H  
0 20 40

50kd  
37kd GSK3β

MHCC97H

0 20 40

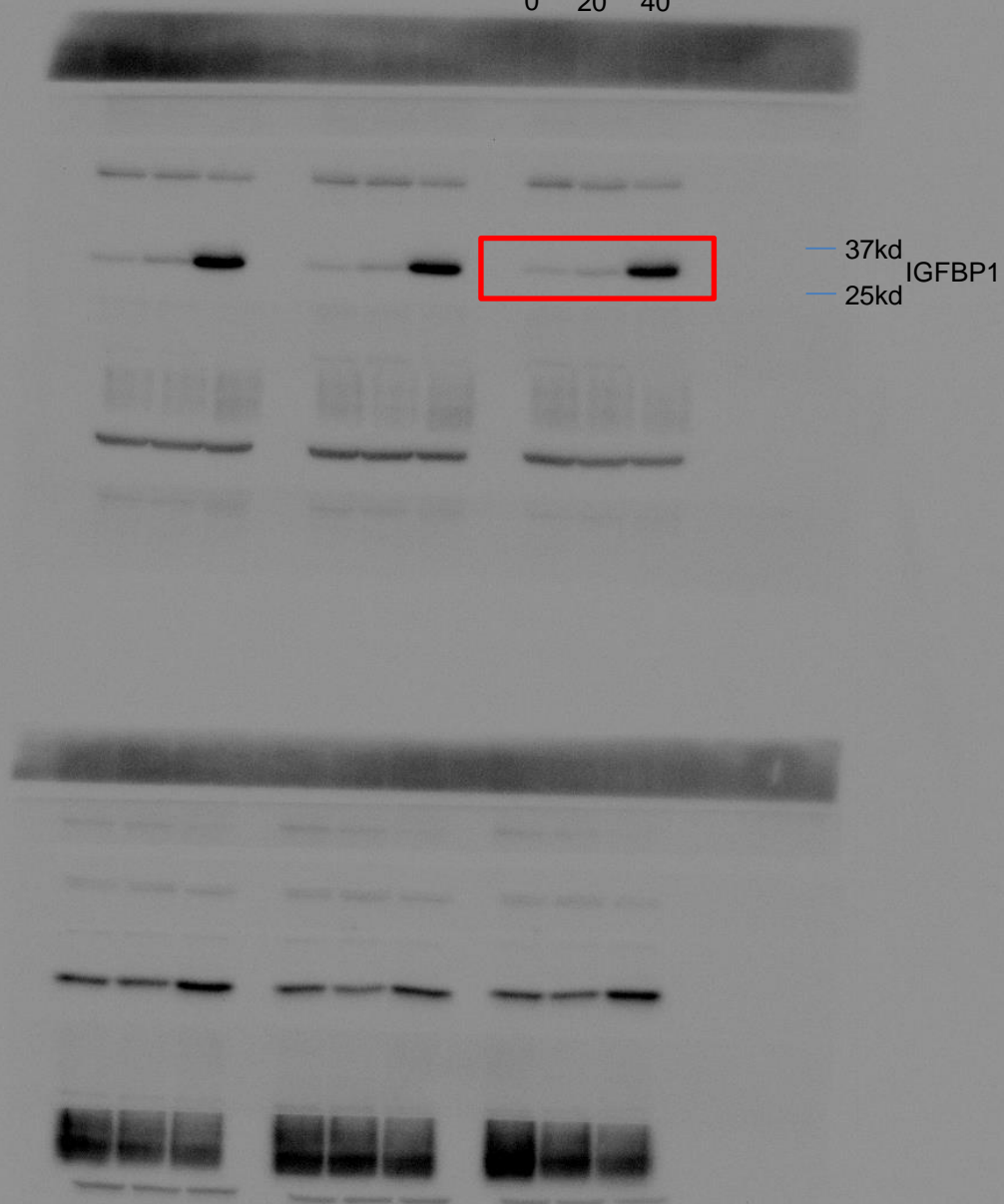

MHCC97H

0 20 40

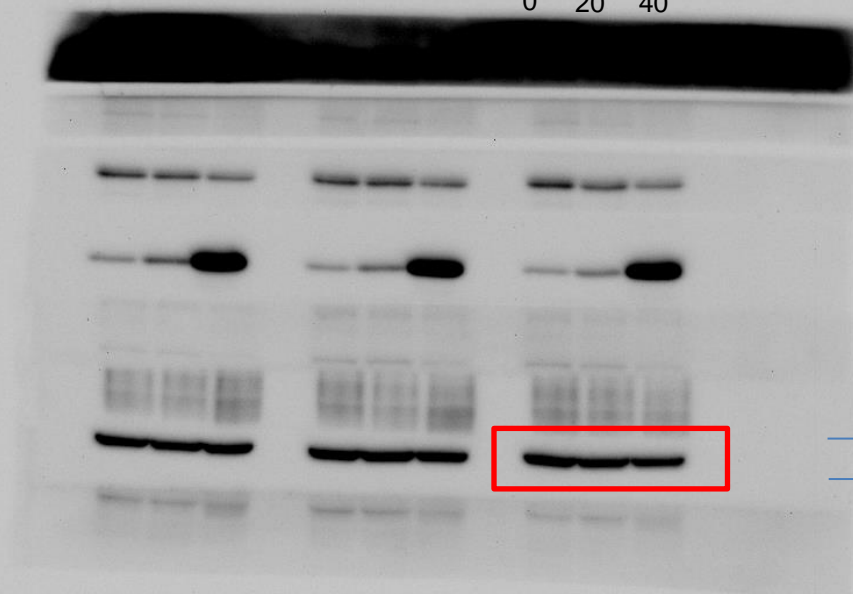

50kd  
37kd  
Actin

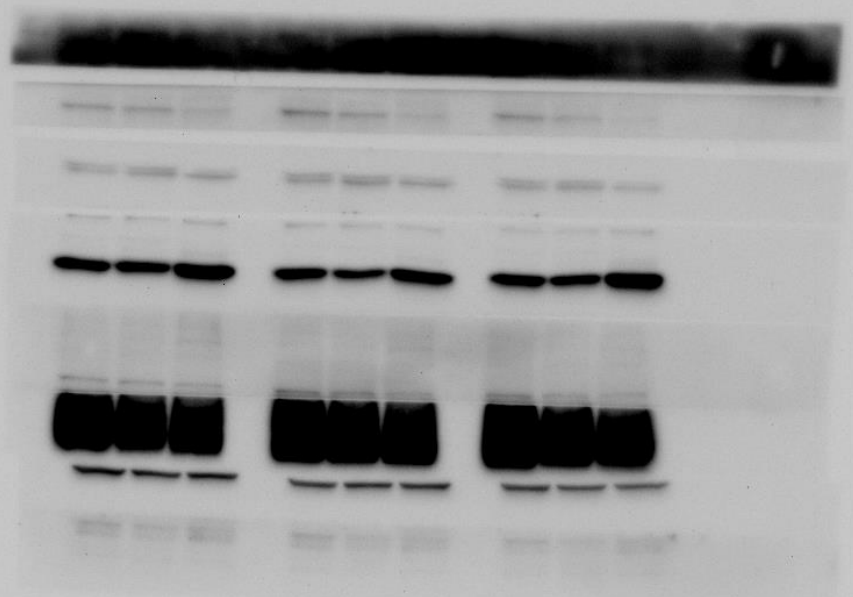

Supplement: Supplementary file 3 [file Data_Sheet_1.zip › PDF-WB-RAW-DATA/WB-Figure 6C-MHCC97H.pdf]

| HepG2 derived tumor |    |    |     |    |    |
|---------------------|----|----|-----|----|----|
| Mock                |    |    | CBD |    |    |
| 1#                  | 2# | 3# | 1#  | 2# | 3# |

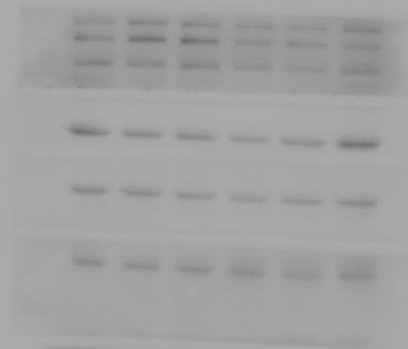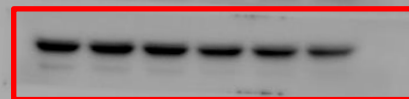

50kd  
37kd

p-GSK3β

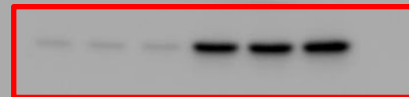

37kd  
25kd

IGFBP1

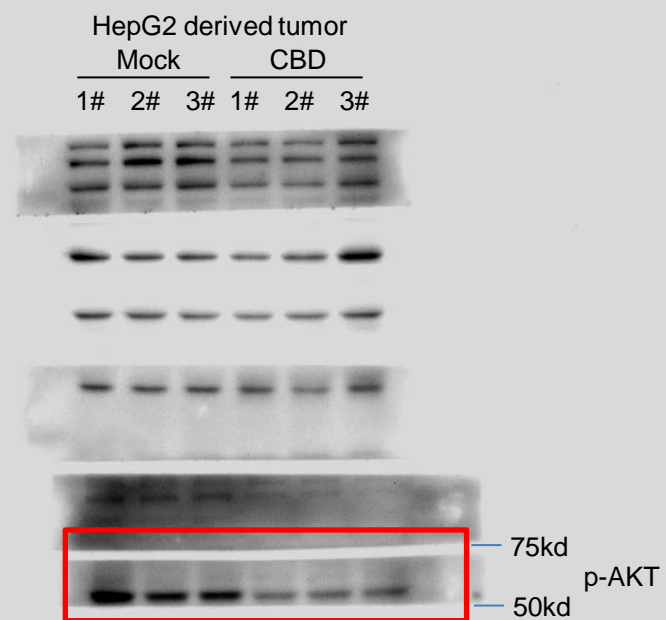

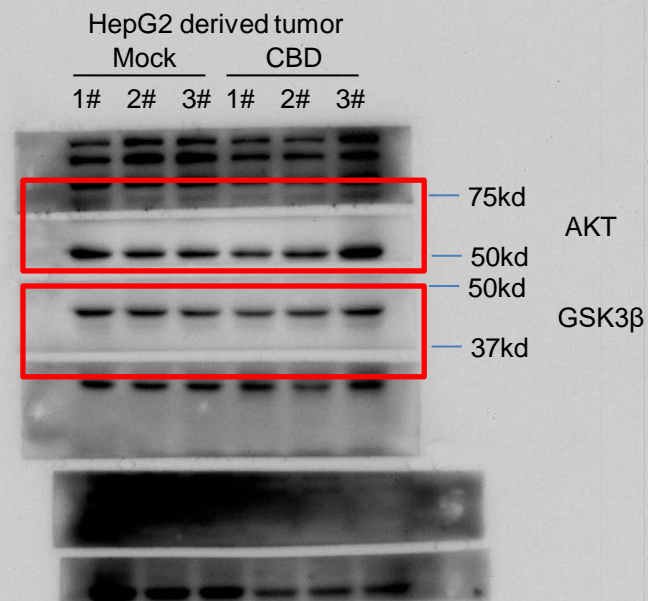

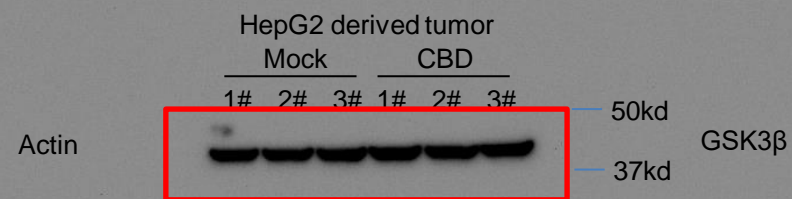

Supplement: Supplementary file 3 [file Data_Sheet_1.zip › PDF-WB-RAW-DATA/WB-Figure 6D.pdf]

HepG2

|   |   |   |   |          |
|---|---|---|---|----------|
| - | + | + | - | CBD      |
| - | - | + | + | shIGFBP1 |

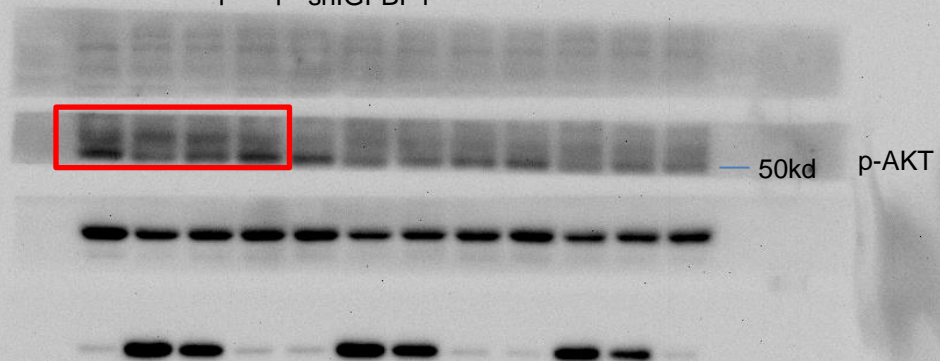

HepG2

|   |   |   |   |          |
|---|---|---|---|----------|
| - | + | + | - | CBD      |
| - | - | + | + | shIGFBP1 |

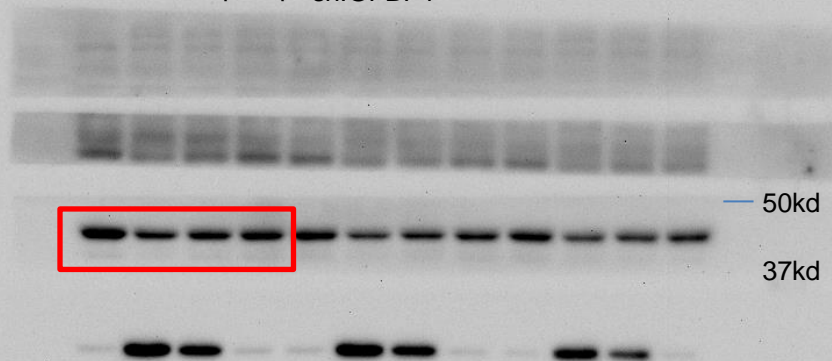

50kd

p-GSK3β

37kd

HepG2

|   |   |   |   |          |
|---|---|---|---|----------|
| - | + | + | - | CBD      |
| - | - | + | + | shIGFBP1 |

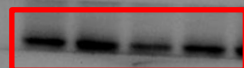

50kd

AKT

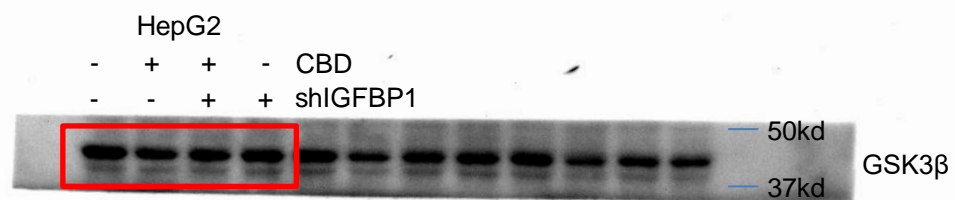

HepG2

|   |   |   |   |          |
|---|---|---|---|----------|
| - | + | + | - | CBD      |
| - | - | + | + | shIGFBP1 |

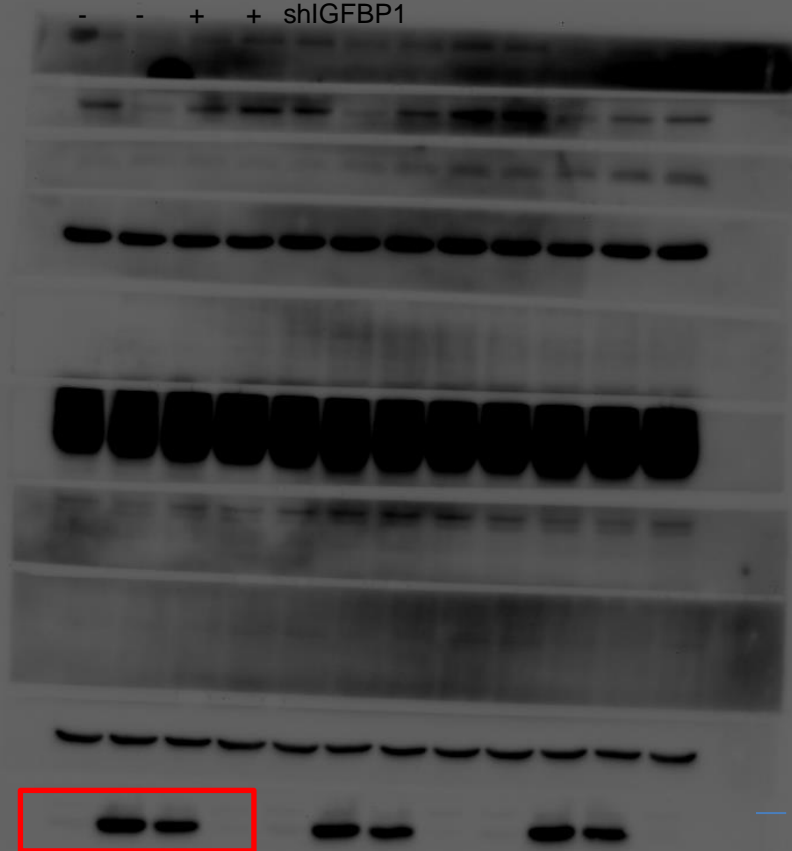

— 37kd

— 25kd

IGFBP1

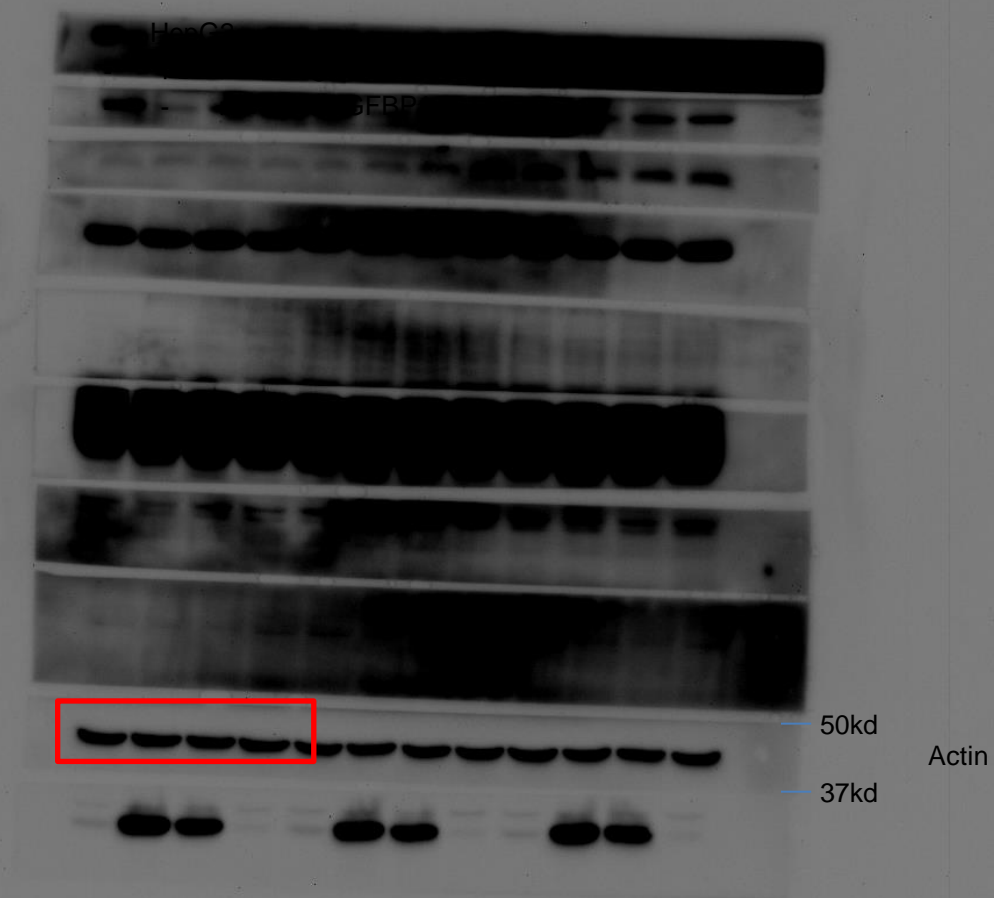

Supplement: Supplementary file 3 [file Data_Sheet_1.zip › PDF-WB-RAW-DATA/WB-Figure 6G.pdf]

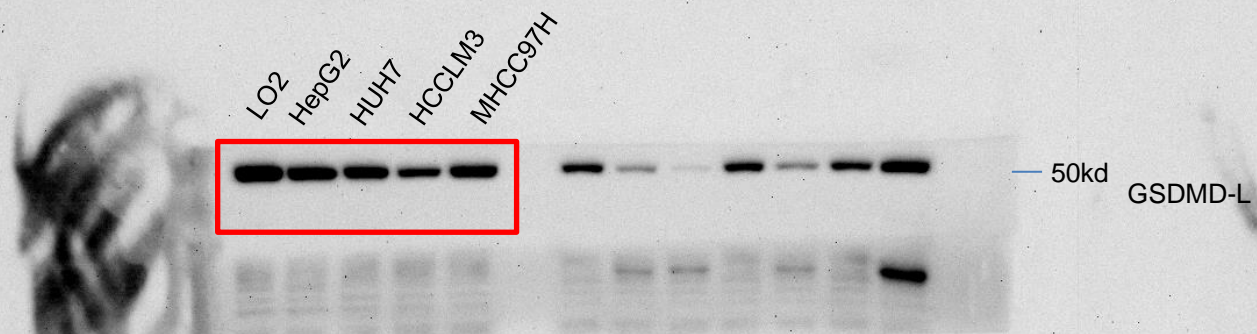

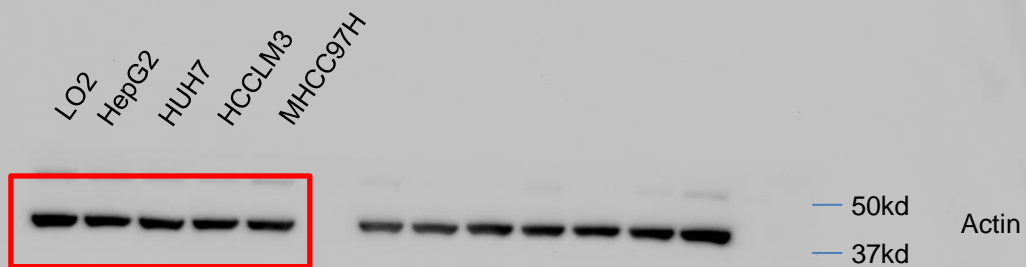

Supplement: Supplementary file 3 [file Data_Sheet_1.zip › PDF-WB-RAW-DATA/WB-Figure S2D-HCC cell line.pdf]

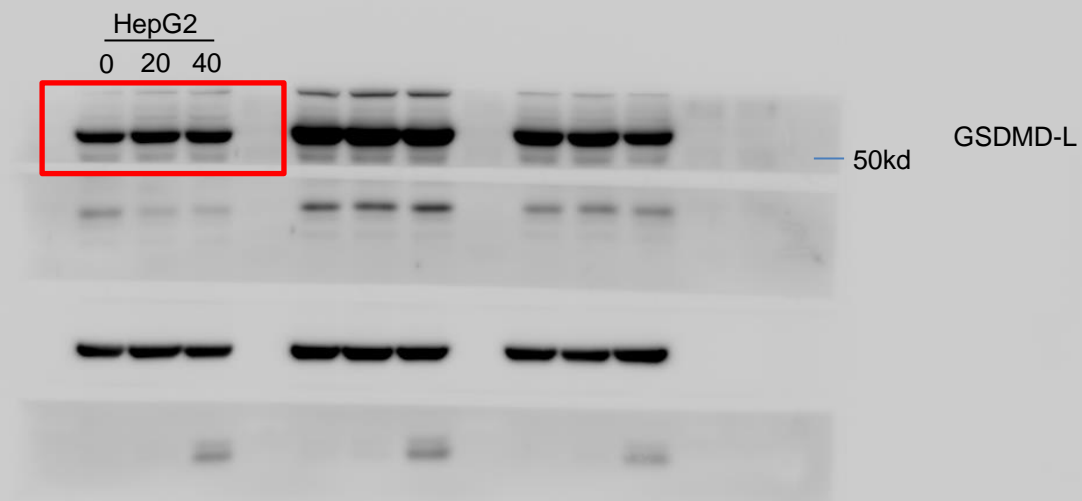

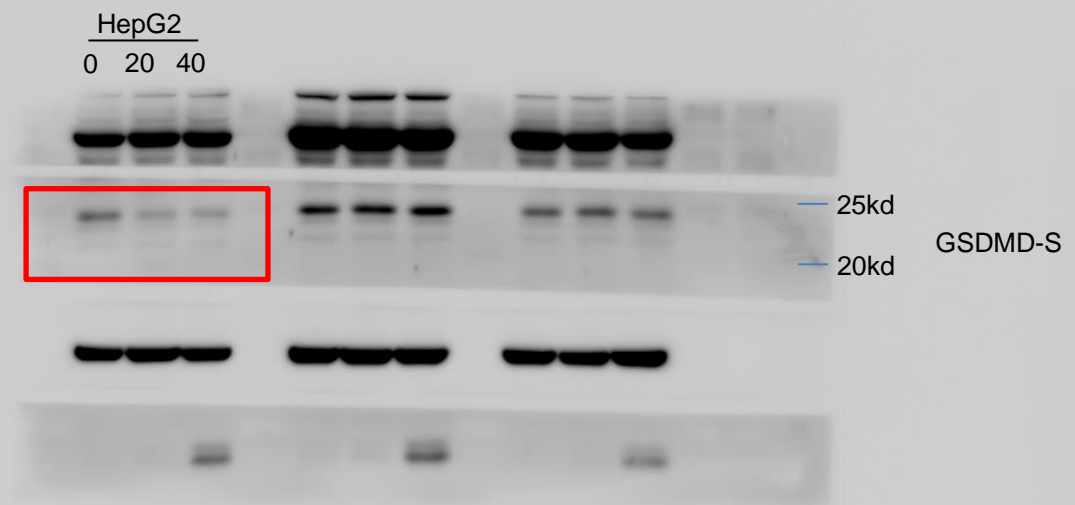

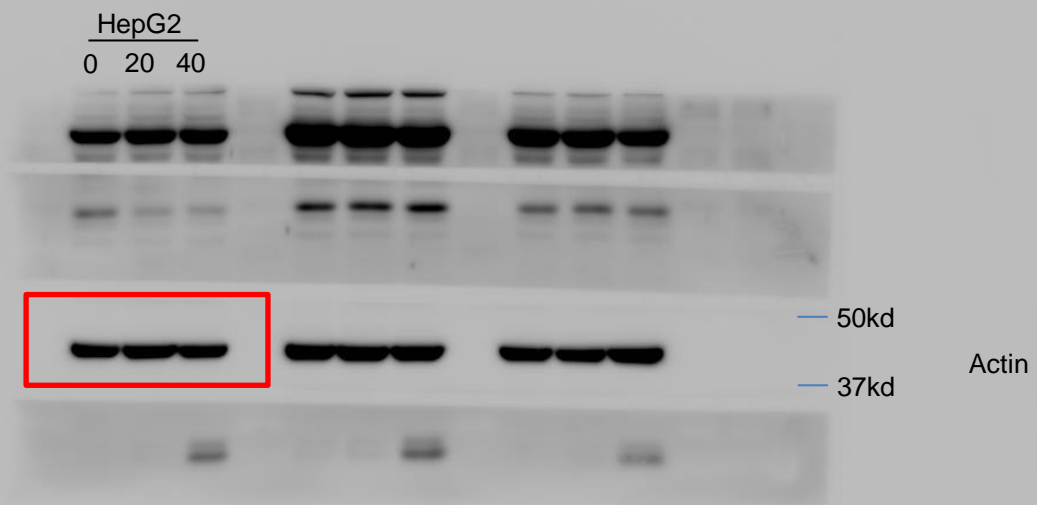

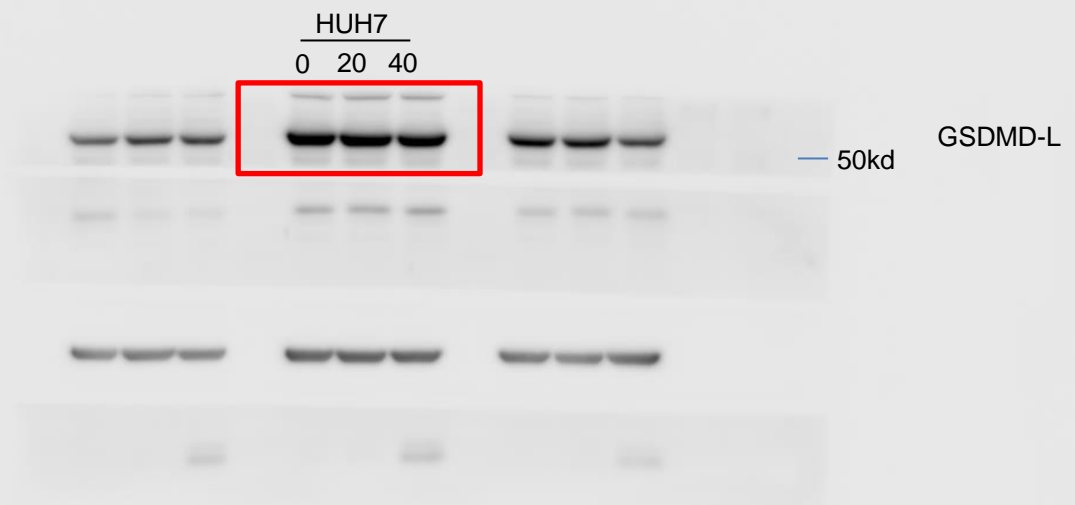

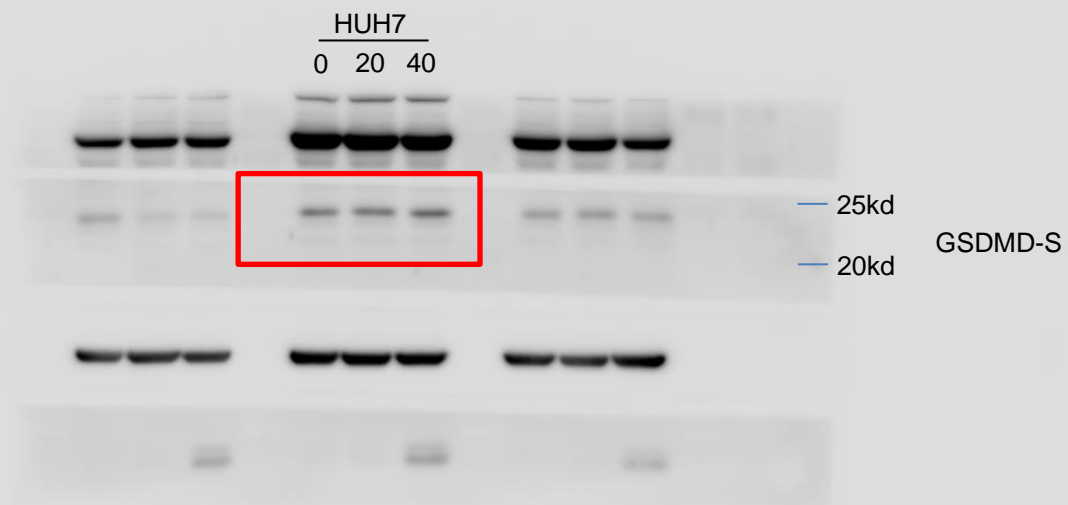

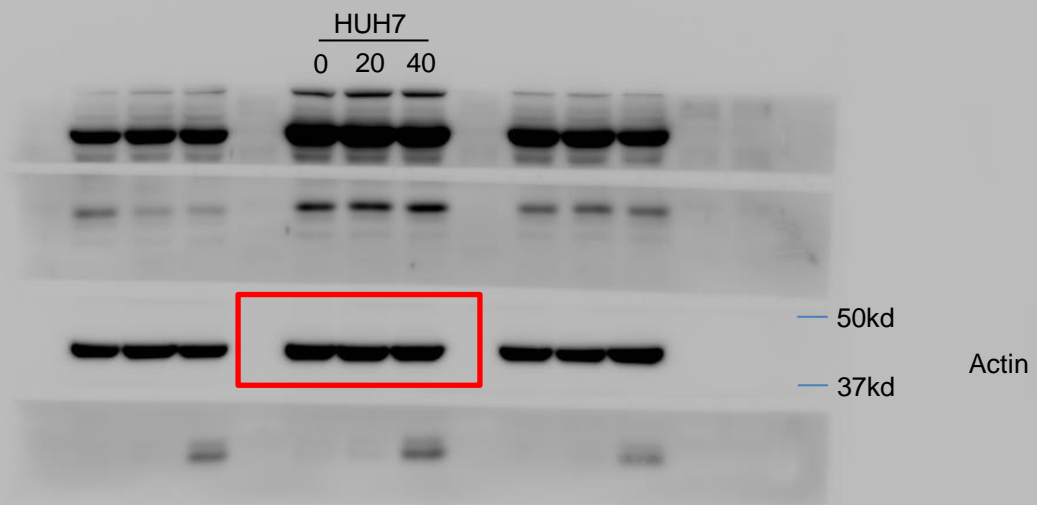

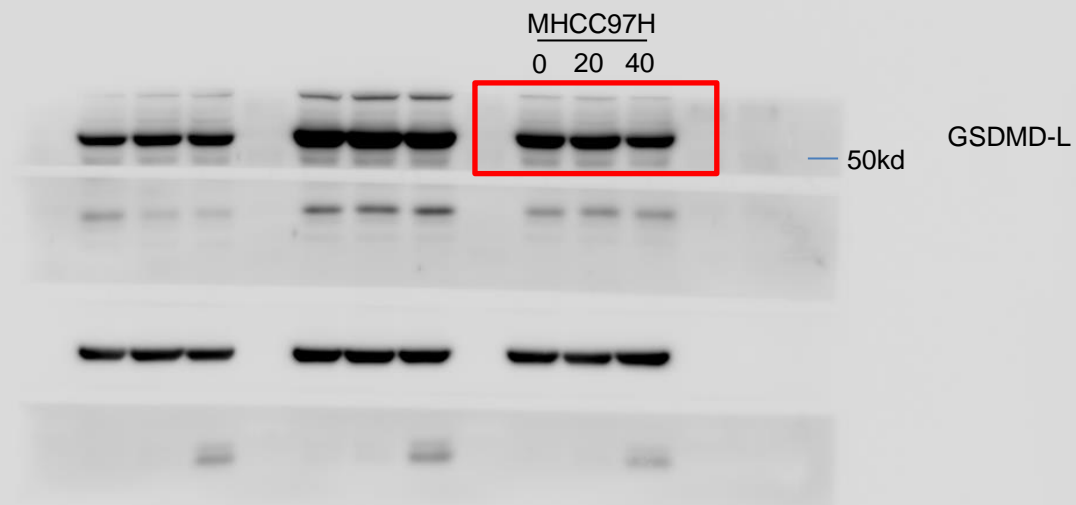

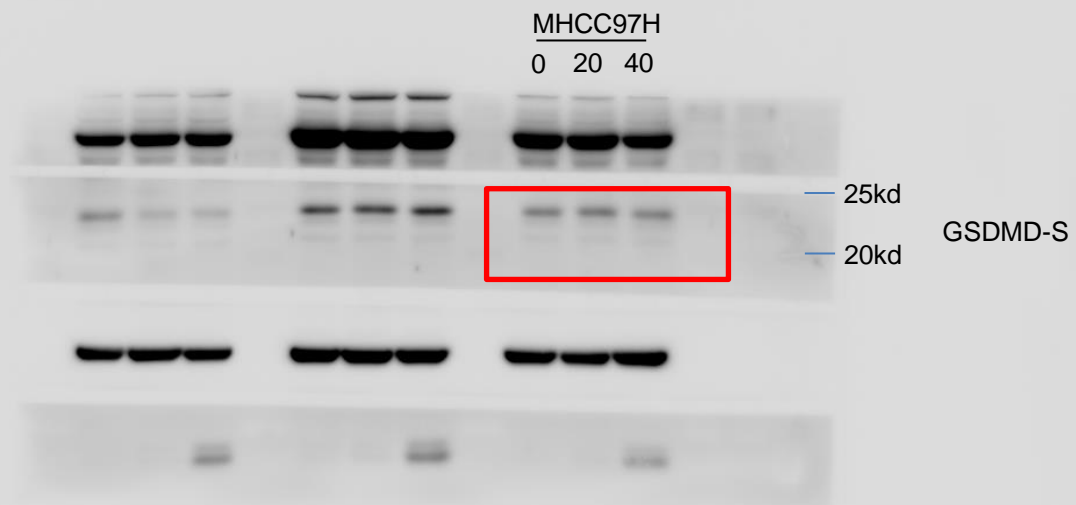

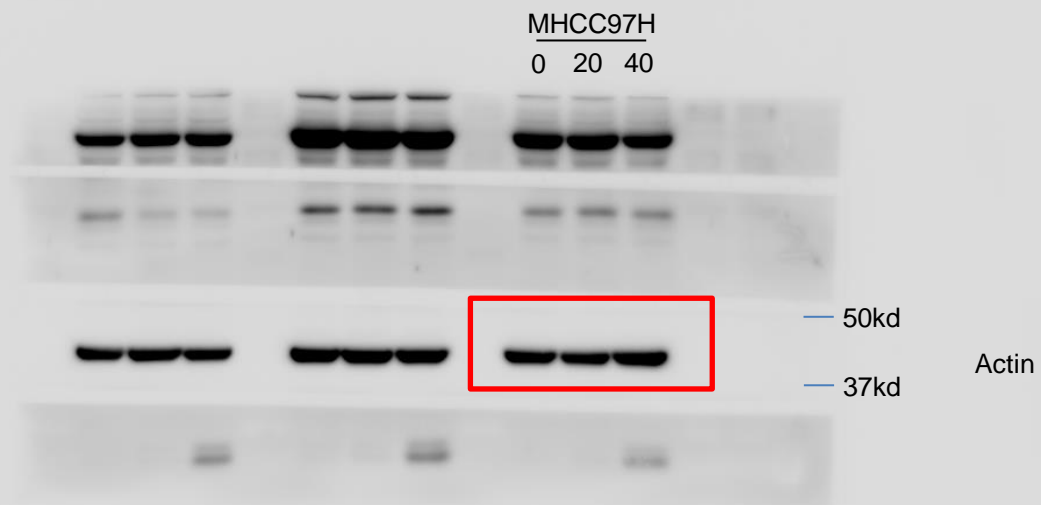

Supplement: Supplementary file 3 [file Data_Sheet_1.zip › PDF-WB-RAW-DATA/WB-Figure S2E-HEPG2-HUH7-MHCC97H.pdf]

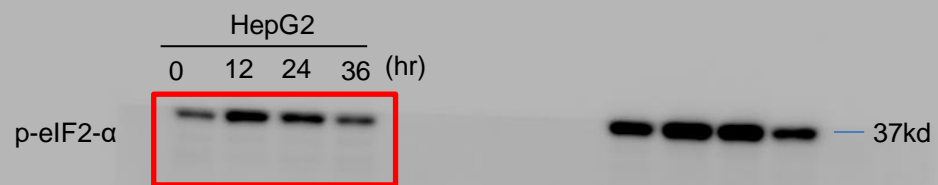

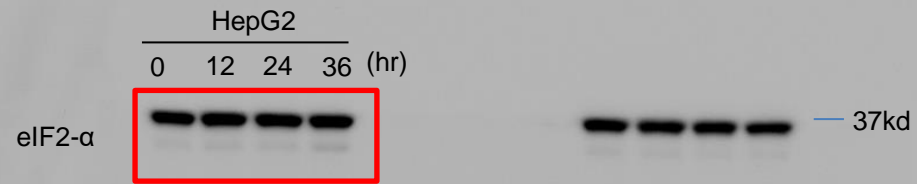

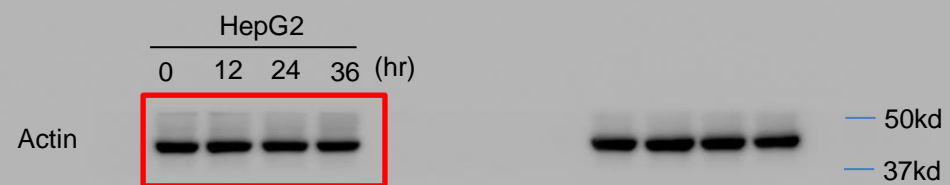

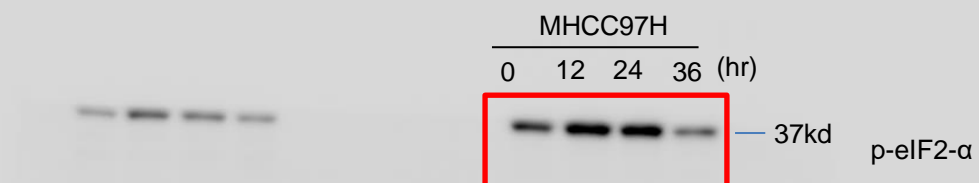

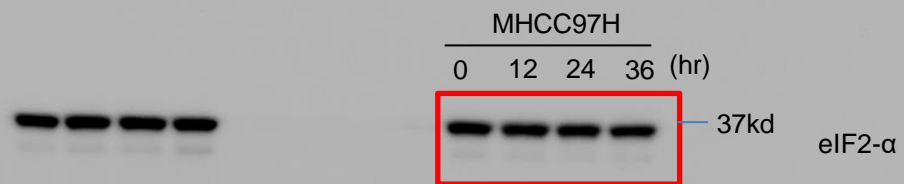

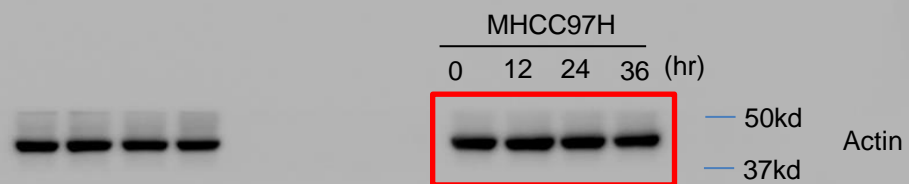

Supplement: Supplementary file 3 [file Data_Sheet_1.zip › PDF-WB-RAW-DATA/WB-Figure S4B.pdf]

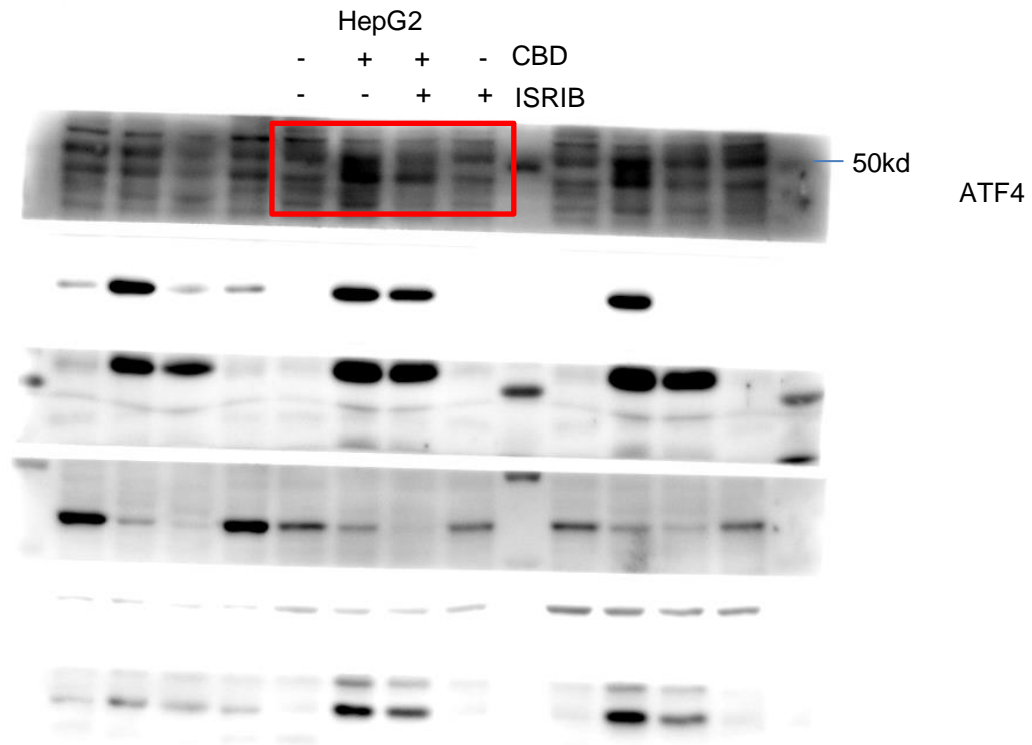

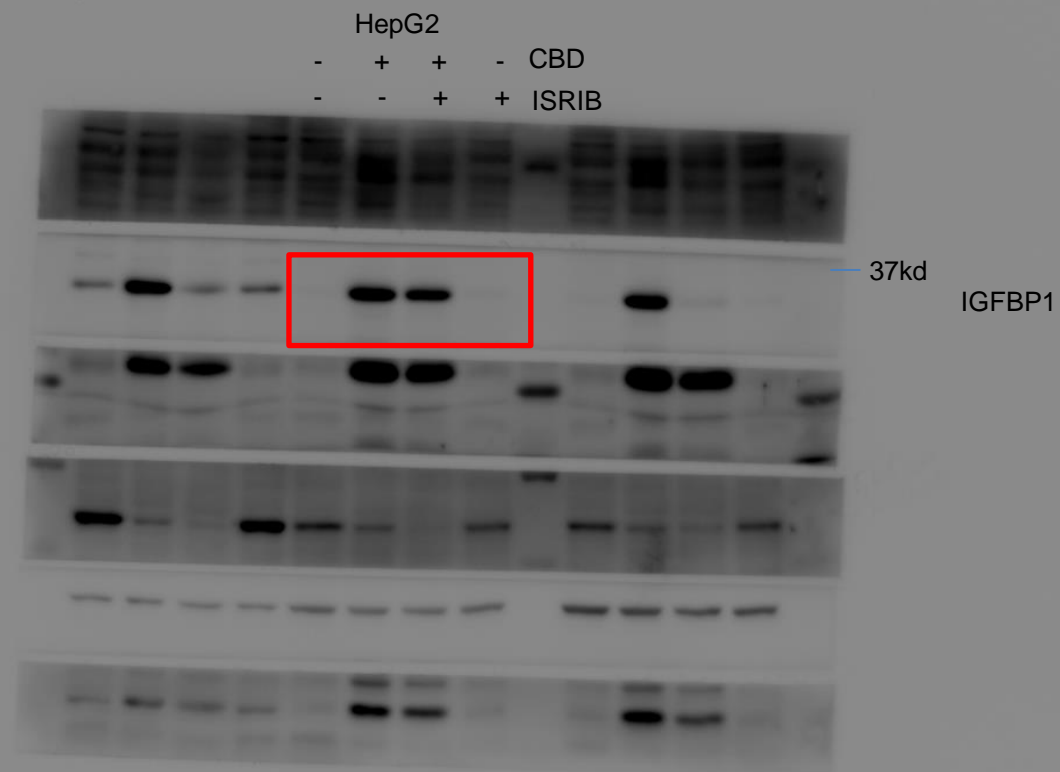

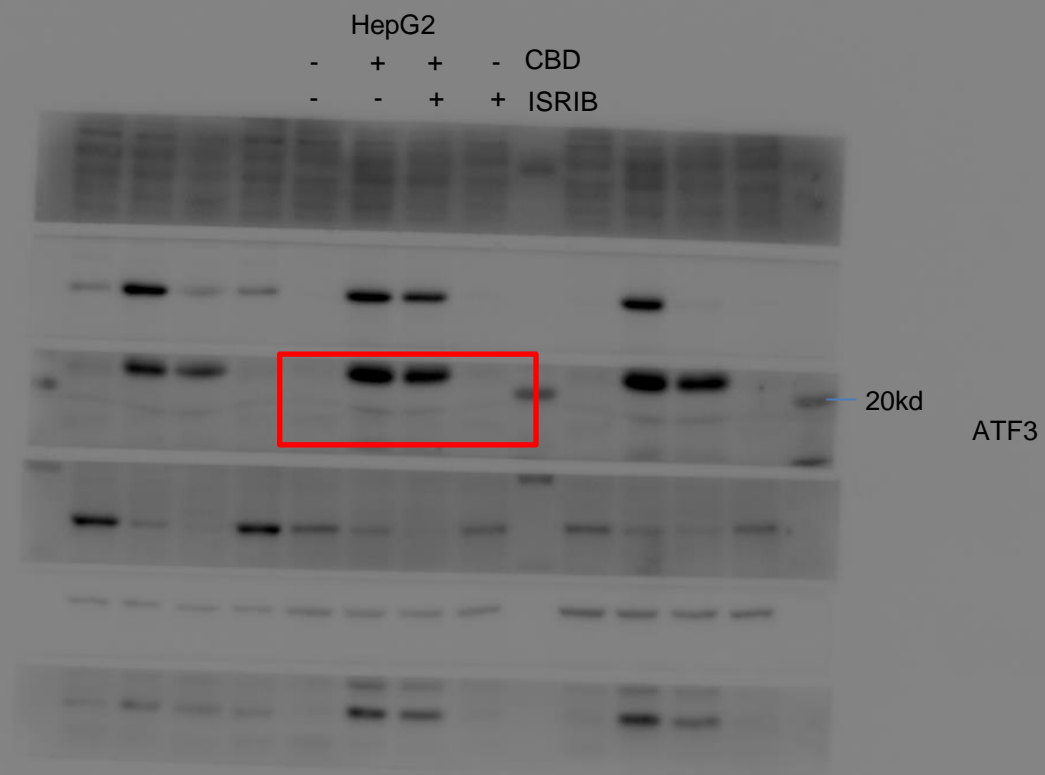

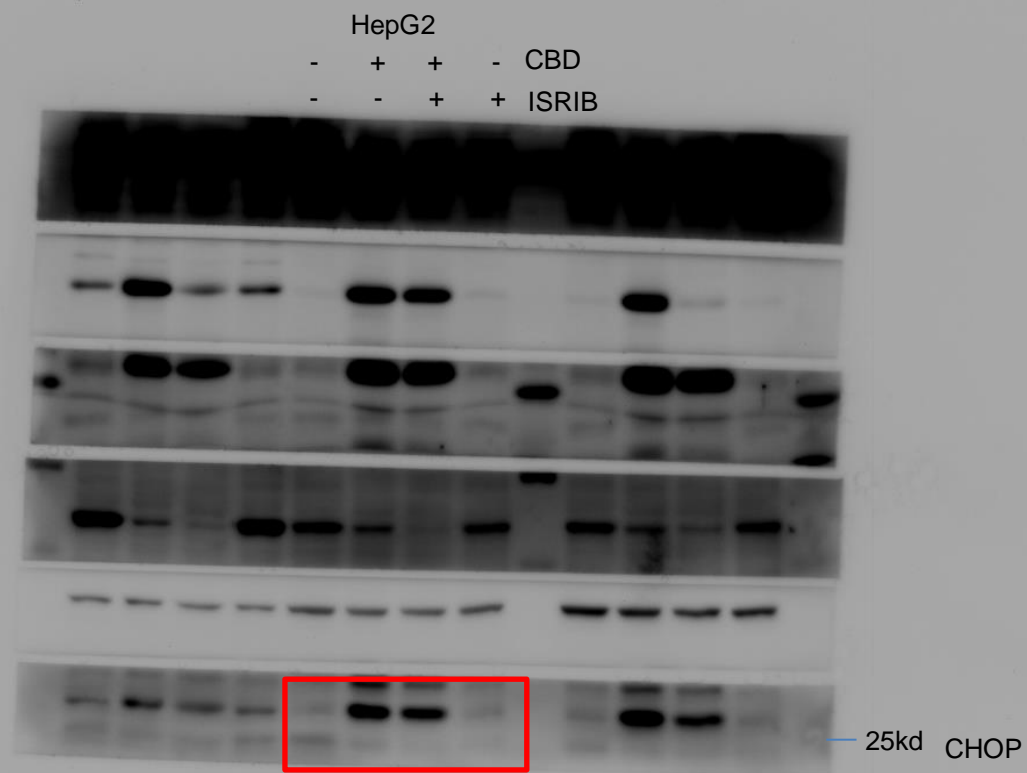

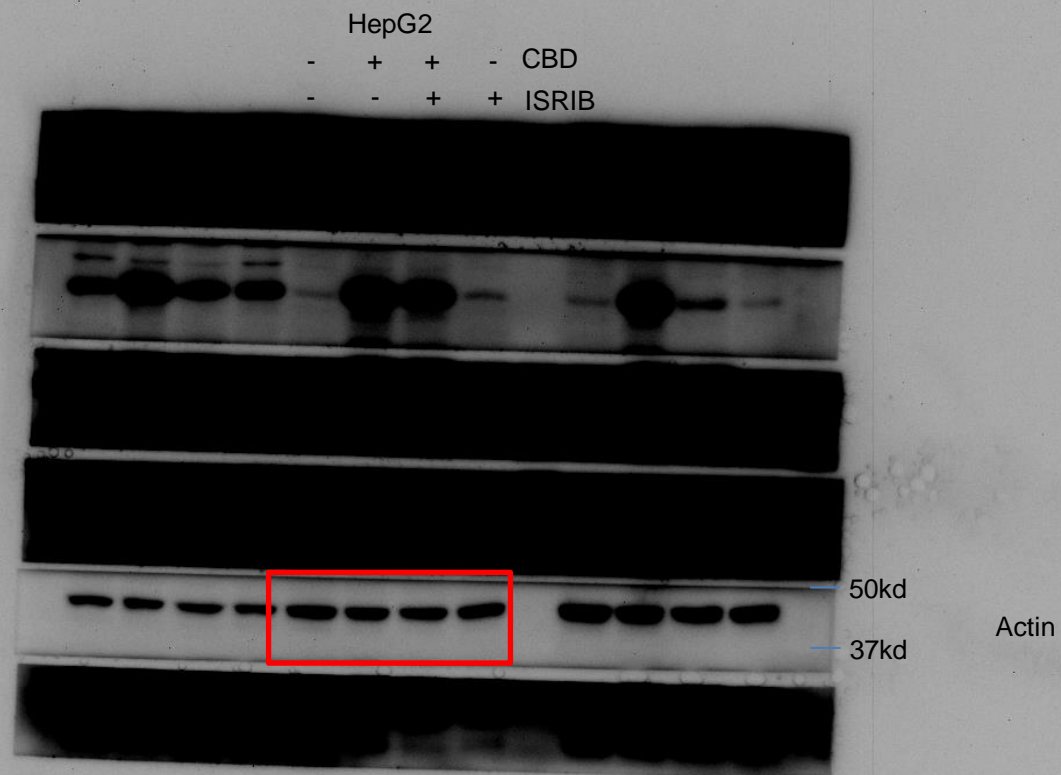

MHCC97H

|   |   |   |   |       |
|---|---|---|---|-------|
| - | + | + | - | CBD   |
| - | - | + | + | ISRIB |

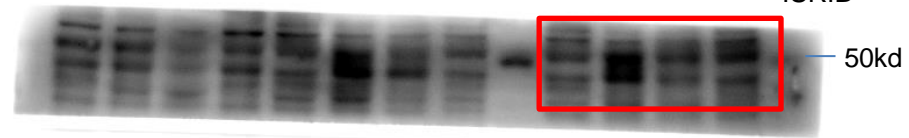

ATF4

MHCC97H

|   |   |   |   |       |
|---|---|---|---|-------|
| - | + | + | - | CBD   |
| - | - | + | + | ISRIB |

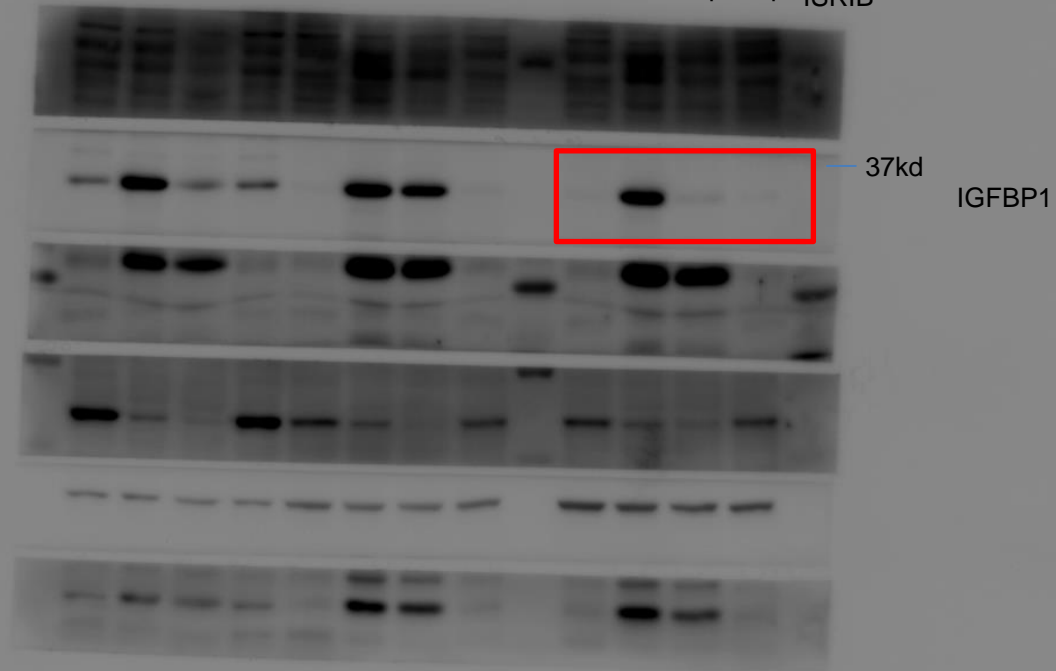

MHCC97H

|   |   |   |   |       |
|---|---|---|---|-------|
| - | + | + | - | CBD   |
| - | - | + | + | ISRIB |

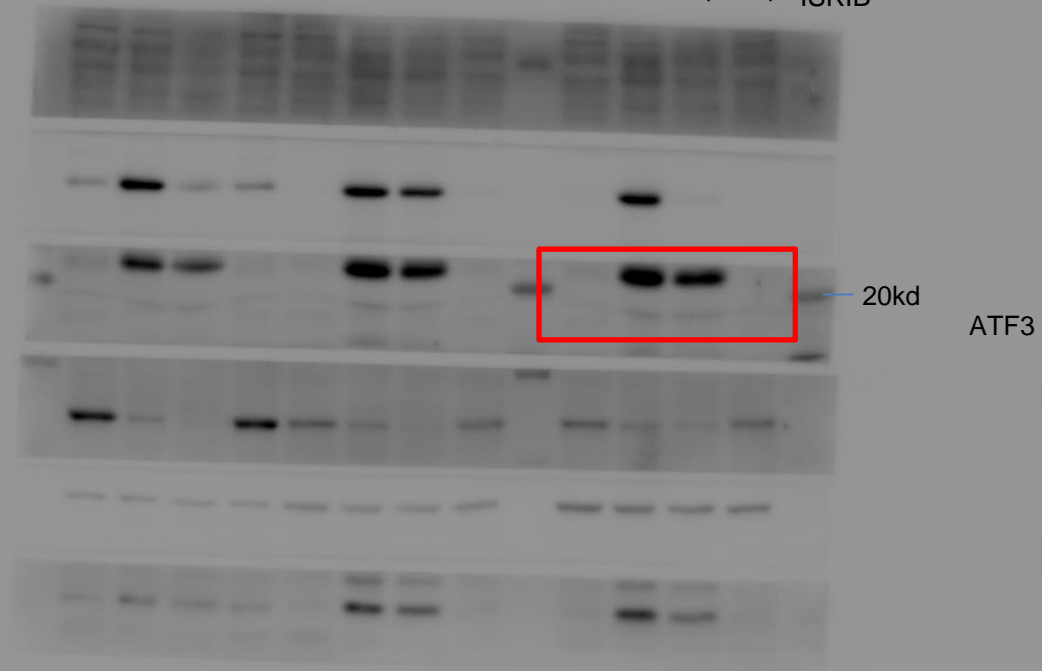

MHCC97H

|   |   |   |   |       |
|---|---|---|---|-------|
| - | + | + | - | CBD   |
| - | - | + | + | ISRIB |

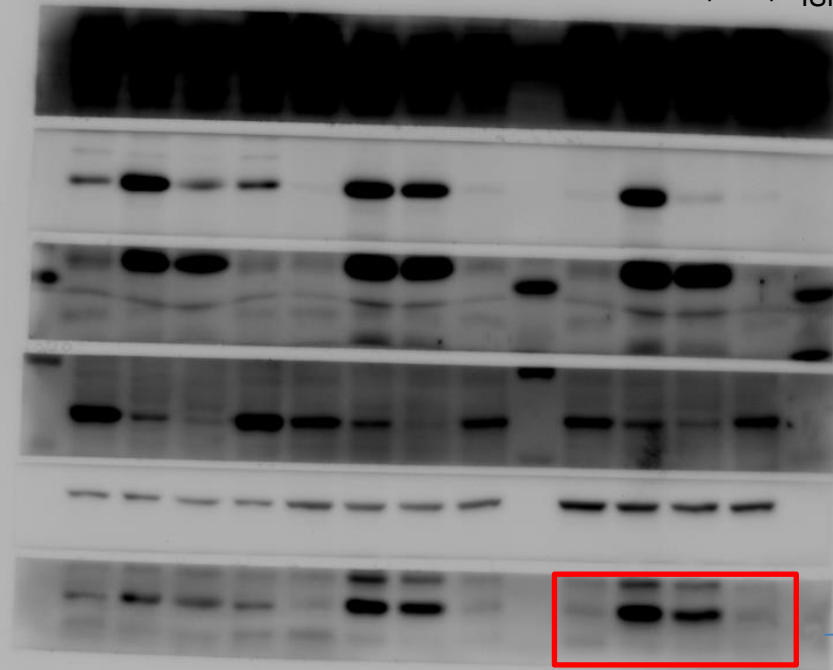

— 25kd CHOP

MHCC97H

|   |   |   |   |       |
|---|---|---|---|-------|
| - | + | + | - | CBD   |
| - | - | + | + | ISRIB |

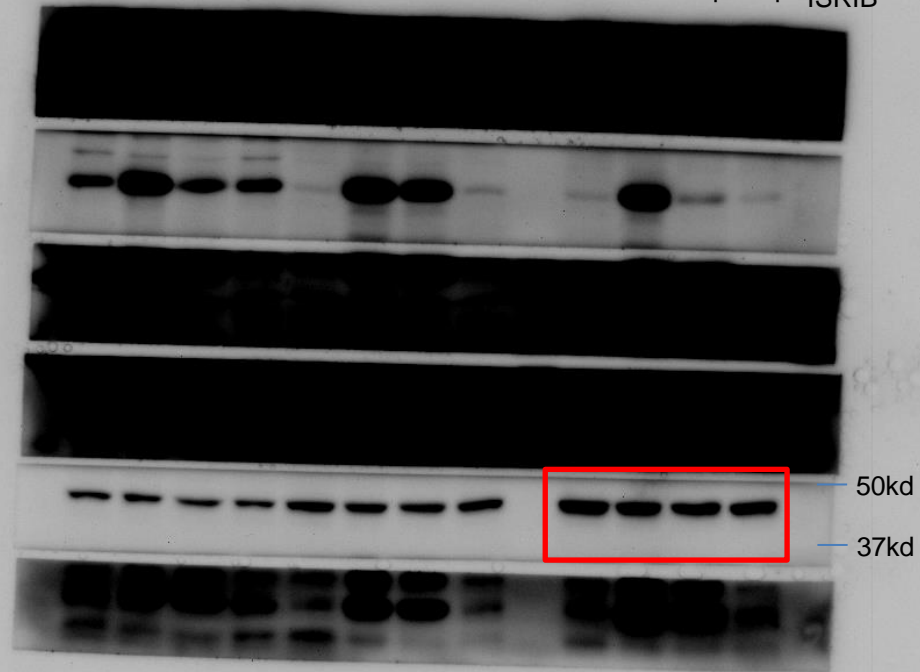

Actin

Supplement: Supplementary file 3 [file Data_Sheet_1.zip › PDF-WB-RAW-DATA/WB-Figure S5A.pdf]
